# Intron retention is a hallmark and spliceosome represents a therapeutic vulnerability in aggressive prostate cancer

**DOI:** 10.1038/s41467-020-15815-7

**Published:** 2020-04-29

**Authors:** Dingxiao Zhang, Qiang Hu, Xiaozhuo Liu, Yibing Ji, Hsueh-Ping Chao, Yan Liu, Amanda Tracz, Jason Kirk, Silvia Buonamici, Ping Zhu, Jianmin Wang, Song Liu, Dean G. Tang

**Affiliations:** 1 Department of Pharmacology and Therapeutics, Roswell Park Comprehensive Cancer Center, Elm and Carlton Streets, Buffalo, New York 14263 USA; 2 College of Biology, Hunan University, Changsha, 410082 China; 3 Key Laboratory of Agricultural Animal Genetics, Breeding and Reproduction of the Ministry of Education, College of Animal Science and Technology, Huazhong Agricultural University, Wuhan, 430070 China; 4 Department of Biostatistics and Bioinformatics, Roswell Park Comprehensive Cancer Center, Buffalo, 14263 New York USA; 5 Department of Epigenetics and Molecular Carcinogenesis, University of Texas MD Anderson Cancer Center, Smithville, 78957 Texas USA; 6 H3 Biomedicine, Inc., 300 Technology Square, Cambridge, Massachusetts 02139 USA

**Keywords:** Cancer, Prostate cancer, Gene regulatory networks, Cancer genomics, Gene regulation

## Abstract

The role of dysregulation of mRNA alternative splicing (AS) in the development and progression of solid tumors remains to be defined. Here we describe the first comprehensive AS landscape in the spectrum of human prostate cancer (PCa) evolution. We find that the severity of splicing dysregulation correlates with disease progression and establish intron retention as a hallmark of PCa stemness and aggressiveness. Systematic interrogation of 274 splicing-regulatory genes (SRGs) uncovers prevalent genomic copy number variations (CNVs), leading to mis-expression of ~68% of SRGs during PCa development and progression. Consequently, many SRGs are prognostic. Surprisingly, androgen receptor controls a splicing program distinct from its transcriptional regulation. The spliceosome modulator, E7107, reverses cancer aggressiveness and inhibits castration-resistant PCa (CRPC) in xenograft and autochthonous PCa models. Altogether, our studies establish aberrant AS landscape caused by dysregulated SRGs as a hallmark of PCa aggressiveness and the spliceosome as a therapeutic vulnerability for CRPC.

## Abstract (web summary)

Dysregulation of mRNA alternative splicing is prevalent in cancers. Here, the authors characterized the landscape of aberrant alternative splicing during the development of prostate cancer, progression and therapeutic resistance and show that splicing modulator, E7107, reduces growth in castration-resistant prostate cancer.

## Introduction

Prostate cancer (PCa) still causes a significant mortality among men worldwide^1^. The prostate is an exocrine gland containing androgen receptor negative (AR^−^) basal and AR^+^ luminal epithelial cells, together with rare neuroendocrine (NE) cells^2,3^. PCa predominantly displays a luminal phenotype and histologically presents as adenocarcinomas (Ads) largely devoid of basal cells^3^. Most primary PCa (pri-PCa) are diagnosed as low to intermediate grade (i.e., Gleason grade ≤ 7), relatively indolent, and treated by radical prostatectomy and/or radiation with a good prognosis. Locally advanced (Gleason grade 9/10) and metastatic PCa are generally treated with androgen deprivation therapy (ADT) using luteinizing hormone releasing hormone agonists/antagonists, which block testicular androgen synthesis. Tumors that have failed this first-line therapy are termed castration-resistant PCa (CRPC) and are further treated with anti-androgens such as enzalutamide (Enza) that interfere with AR functions. Enza only extends CRPC patients’ lives by 4–5 months before tumor recurrence. Although the majority of CRPC and Enza-resistant tumors histologically present as Ad (i.e., CRPC-Ad), a significant fraction (up to 25%) of them evolve to an aggressive, AR^−^ indifferent disease with NE features called CRPC-NE^4^. In general, all CRPC are relatively undifferentiated and, molecularly, basal/stem-like^3,5^, highlighting lineage plasticity in facilitating treatment resistance and progression^6^. Most metastatic CRPC (mCRPC), including both CRPC-Ad and CRPC-NE subtypes, remains lethal.

Dysregulation in pre-mRNA alternative splicing (AS) is emerging as a hallmark of cancer^7^. Nearly all multi-exon human genes undergo AS, a tightly regulated process that dramatically expands diversity of the transcriptome and proteome encoded by the genome^7,8^. As an essential process for removing non-coding introns and ligating flanking exons to produce mature mRNA in eukaryotic cells, AS is performed by a dynamic and flexible macromolecular machine, the spliceosome. In addition to the core subunits that constitute five small nuclear ribonucleoprotein particles, the spliceosome contains many other auxiliary splicing-regulatory proteins (SRPs) including families of the serine- and arginine-rich (SR) proteins, heterogeneous nuclear ribonucleoproteins (hnRNP), and other modulatory factors^8^. In this study, we refer to the genes encoding the spliceosome core subunits and SRPs, broadly, as splicing-regulatory genes (SRGs) (Supplementary Data 1). Aberrant AS is prevalent in human cancers^9^ and many cancer-specific splicing events contribute to disease development and progression^7,8^. Since the initial discovery of frequent point mutations in the core spliceosome subunits in myelodysplastic syndromes and, later, in hematological malignancies^7^, splicing dysregulation has been appreciated as a major contributor to cancer phenotypes. In parallel, therapeutic targeting of mis-splicing by small molecules presents an innovative approach for treating hematological malignancies bearing core subunit mutations^8^ and solid tumors driven by MYC^10^. Nevertheless, despite increasing elucidation of global and cancer-associated splicing features by RNA sequencing (RNA-seq) analyses of primary tumors and normal tissues^9^, the underlying molecular mechanisms and functional relevance of splicing misregulation in cancer, especially in solid tumors, remain largely undefined.

Importantly, significantly fewer recurrent mutations in the core spliceosome genes have been detected to date in solid tumors^11^, suggesting a fundamental and mechanistic difference in splicing misregulation in hematological vs. solid cancers. Recently, global analyses of aberrant AS landscape across many human cancer types, including PCa, have been reported using RNA-seq data in The Cancer Genome Atlas (TCGA)^9,12^, but these studies generally overlooked PCa and only analyzed pri-PCa, leaving behind life-threatening mCRPC. Furthermore, regarding the functional consequence of splicing dysregulation in PCa, previous studies have mainly focused on a few well-known genes typified by AR and CD44^13^, and the potential biological impact and clinical relevance of global splicing abnormalities in PCa remains unclear. Here we focus on PCa and provide a comprehensive characterization of the global AS landscape during disease development and progression and upon treatment failure. We report that intron retention (IR) represents the most salient and consistent feature across the spectrum of PCa entities and positively correlates with PCa stemness and aggressiveness. We also systematically analyze the dysregulated SRGs and correlate altered SRGs with aberrant AS patterns and outcomes in PCa, and examine the deregulated pathways affected by aberrant splicing events. Finally, we demonstrate that splicing misregulation can be exploited therapeutically for treating CRPC.

## Results

### Splicing dysregulation correlates with PCa progression

**Fig. 1:**
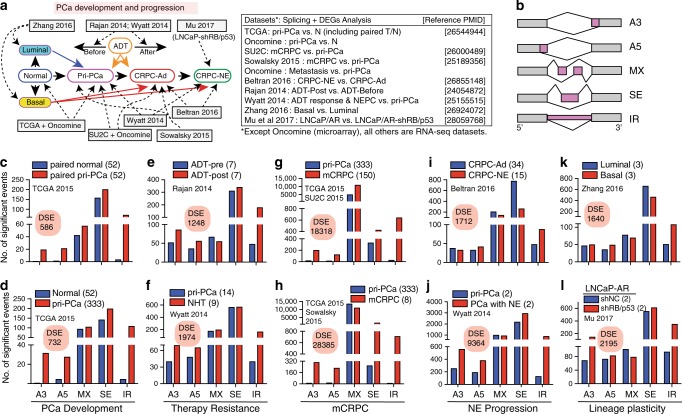
Severity of splicing dysregulation correlates with PCa evolution. **a** Schematic illustrating the spectrum of PCa development, therapy resistance and metastatic progression, and related datasets (Oncomine and RNA-seq with reference PMID provided) used for pairwise comparisons. The spectrum of PCa progression is indicated by Normal → Pri-PCa → CRPC-Ad → CRPC-NE, and the relationship of each dataset to specific PCa progression stages is indicated by various arrows. For example, we have previously shown that normal prostate luminal and basal cell profiles molecularly resemble pri-PCa (blue arrow) and aggressive subtypes (i.e., CRPC-Ad and CRPC-NE, red arrow), respectively^5^. On the other hand, prostate tumors before and after ADT are related to pri-PCa and CRPC-Ad (dark yellow), respectively^5^. **b** Five main types of AS patterns analyzed in the present study. A3, alternative 3′ splice sites; A5, alternative 5′ splice sites; MX, mutually exclusive exons; SE, exon skipping; IR, intron retention. **c**–**l** Alterations in AS landscape during PCa development and progression. Two related datasets are interrogated and compared for each PCa stage. Shown are splicing patterns and the number of DSEs decoded by rMATS. DSEs, differentially spliced events. See Supplementary Data 3 for details.

To determine the global AS dysregulation in PCa development and progression, we employed two AS mapping algorithms, rMATS^14^ and SUPPA^15^, to annotate RNA-seq datasets (Supplementary Data 2) encompassing pri-PCa and normal (N) prostate tissues^16^, advanced PCa treated with ADT^17,18^, CRPC-Ad^19,20^, and CRPC-NE^4,18^ (Fig. 1a). We defined “progression” generally as stages beyond pri-PCa and as disease entities that were more aggressive in a comparative manner. Five main AS patterns, including alternative 3′ and 5′ splice sites (A3 and A5, respectively), mutually exclusive exons (MX), exon skipping (SE) and IR, were examined (Fig. 1b). Splicing events with a cutoff of ΔPSI > 0.1 and false discovery rate (FDR) < 0.1 (for rMATS) or *p* < 0.05 (for SUPPA) were considered statistically significant.

Comparative analyses of either bulk or paired tumors and normal tissues indicated that pri-PCa possessed more AS events (~1.9-fold by rMATS; ~1.7-fold by SUPPA) with preferential increase in A3, A5, and IR (Fig. 1c, d and Supplementary Data 3). PCa post ADT (Fig. 1e) or subjected to neo-adjuvant hormone therapy (NHT; Fig. 1f) also displayed increased differentially spliced events (DSEs), suggesting a treatment-induced reshaping of global AS pattern that might have contributed to therapy resistance. Strikingly, mCRPC exhibited an exponential increase in DSEs, with noticeable increase in A3, A5, SE, and IR (Fig. 1g, h). Within CRPC, CRPC-NE harbored a distinct splicing landscape relative to CRPC-Ad, although a notably smaller number of DSEs were observed than that in the CRPC-Ad vs. pri-PCa comparison (1712 vs. 18,318; Fig. 1i). Interestingly, when comparing pri-PCa vs. pri-PCa with NE differentiation post NHT^18^, we identified 9364 DSEs (Fig. 1j). Notably, rMATS-based AS mapping with an FDR < 0.05 generated virtually identical results to those using FDR < 0.1 (Supplementary Fig. 1a-c). Also, mapping with SUPPA revealed overall similar dysregulated AS patterns and progressive increase in DSEs in the spectrum of PCa evolution (Supplementary Fig. 1d, e) albeit SUPPA by nature tended to detect more splicing events (see Methods). Together, these results suggest that PCa development is accompanied by increased AS events, and that castration resistance and, in particular, metastasis are characterized by further significant increases in AS events.

As lineage plasticity facilitates therapeutic resistance and tumor progression^6,21^, we determined the human prostate epithelial lineage-specific AS patterns as basal cells represent the main pool of prostate stem cells (SCs) and molecularly resemble aggressive PCa subtypes^5^. Results revealed distinct AS profiles for basal vs. luminal cells, with more IR found in basal cells (Fig. 1k). To determine whether basal-specific splicing profile also resembles that in aggressive PCa, we performed comparative gene set enrichment analysis (GSEA) and found that PCa with aggressive phenotypes (mCRPC and CRPC-NE) generally possessed a global basal-like AS profile (Supplementary Fig. 1f). Experimentally, silencing of tumor suppressor (TS) genes *TP53* and *RB1* in LNCaP/AR cells enables a lineage switch from AR^+^ luminal cells to AR^−^ basal-like cells^21^. Consistently, a large number of DSEs were observed in LNCaP/AR cells with *RB1/TP53* knockdown (Fig. 1l), suggesting that plasticity driven by loss of *RB1/TP53* is accompanied by a global shift in the AS landscape. Remarkably, GSEA indicated that the AS signatures of LNCaP/AR cells deficient in RB1/TP53 were significantly enriched in mCRPC compared with pri-PCa (Supplementary Fig. 1g). These results suggest that inherent lineage differences in normal prostate epithelial cells and induced lineage plasticity in PCa cells are also accompanied by dysregulated AS patterns that correlate with increased aggressiveness.

### AS dysregulation impacts PCa biology

We explored the potential impact of AS dysregulation on PCa biology by overlapping the splicing-affected genes (SAGs) and differentially expressed genes (DEGs) (Supplementary Fig. 2a), and by Gene Ontology (GO) analysis of SAGs identified in each PCa stage (Supplementary Fig. 2b-d). Results indicated that the majority of AS events minimally changed the bulk gene expression and SAGs were enriched in many cancer-associated functional categories with both convergence and specificity identified in a context-dependent manner (Supplementary Note 1). By using an isoform-specific alignment algorithm^22^, we established distinct splice isoform signatures representative of different PCa stages (Supplementary Fig. 3a). Many PCa relevant genes (e.g., *AR*, *CD44*^23^, *MEAF6*^24^, and others) displayed isoform switch during cancer development and progression (Supplementary Fig. 3b-e and Supplementary Note 1). To further assess the involvement of these clinically relevant isoforms in regulating PCa biology, we overlapped the top 400 differentially expressed isoforms (DEIs) upregulated in CRPC-Ad (vs. pri-PCa) with the retained exon events (i.e., SE with ΔPSI > 0.1) identified in experimental CRPC systems (LNCaP-AI/AD and LAPC9-AI/AD pairs, see below)^25^, aiming to find clinically relevant isoforms that were caused by aberrant splicing. One common gene *SYT7*, which has multiple isoforms with 4 variable exons (Supplementary Fig. 4a, upper), was chosen for interrogation. Both PC3 and DU145 expressed multiple *SYT7* transcripts, and small interfering RNAs (siRNAs) that specifically targeted the alternative exons efficiently knocked down the endogenous mRNAs harboring the indicated exons (Supplementary Fig. 4a, bottom). Colony formation (Supplementary Fig. 4b) and proliferation (MTT (3-(4,5-dimethyl-2-thiazolyl)- 2,5-diphenyl-2H-tetrazolium bromide)) assays (Supplementary Fig. 4c) revealed inhibitory effects with knockdown of either alternative exon 1 or 3. Isoform-specific siRNA treatments also diminished or abolished sphere formation (especially large spheres) in PC3 and DU145 cells (Supplementary Fig. 4d, e), suggesting a potential role of *SYT7* isoforms in regulating PCa stemness. These analyses indicate that splicing abnormalities impact PCa biology, partially, via switching the isoform expression of key cancer-related genes.

### Elevated IR is a hallmark of PCa aggressiveness and stemness

**Fig. 2:**
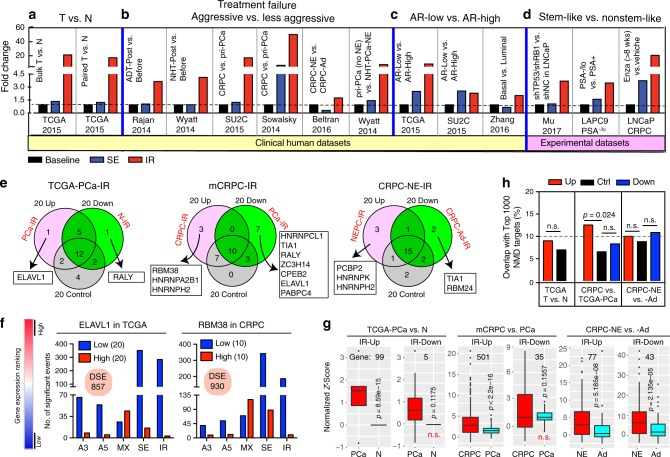
IR represents a consistent hallmark of PCa stemness and progression. **a**–**d** Changes in IR and SE across the 14 comparisons detected by rMATS. Both clinical (**a**–**c**) and experimental (**d**) RNA-seq datasets were used. The data were presented as fold changes in a comparative manner and baseline refers to 1. **e** RBP motif analysis of retained introns specific to the PCa stages indicated. A total of 95 RBPs are examined and shown are the top 20 genes ranked by a binding score that takes into account both binding frequency and binding strength for each RBP. **f** DSEs associated with high or low expression level of ELAVL1 and RBM38 in pri-PCa and CRPC-Ad, respectively. **g** Pairwise comparison of expression of the genes showing significant IR events during PCa progression. Expression variability is quantified for each gene as a *Z*-score relative to the mean expression in normal prostate samples. Genes exhibiting both up- and downregulated IR events are removed, and the resultant gene number is indicated. Within the plots, the center lines represent median values, box edges are 75th and 25th percentiles, and whiskers denote the maximum and minimum values, respectively. Significance was calculated by two-tailed paired Student’s *t*-test (NS, not significant). **h** Overlap of significant IR-bearing genes with a high-confidence set of 1000 human NMD targets. Significance was calculated by two-tailed *χ*^2^-test (NS, not significant). DSEs, differentially spliced events; RBP, RNA-binding protein; NMD, nonsense-mediated mRNA decay. Source data are provided as a Source Data file.

We consistently observed increased IR across the spectrum of PCa evolution, whereas the SE represented the most abundant splicing type (Fig. 1c–l). We focused our subsequent studies on IR for it is the least studied AS type^26^. We observed a >18-fold increase in IR in pri-PCa vs. N (Fig. 2a), consistent with a previous report^26^. PCa progression is tightly associated with ADT failure and cellular plasticity towards stemness^6,21,25^. In six different contexts, we consistently observed a preferential upregulation of IR in association with therapy-resistant, aggressive, and metastatic PCa (Fig. 2b). Similar IR upregulation was observed in prostate tumors and epithelial cells displaying low vs. high AR activity (Fig. 2c; see below). Interestingly, increased IR was also found in cancer SC (CSC)-enriched PSA^−/lo^ cell population isolated from LAPC9 xenografts^27^, basal-like LNCaP cells depleted of *TP53* and *RB1*^21^, and LNCaP-CRPC cells that survived long-term Enza treatment^28^ (Fig. 2d). Of note, SUPPA produced similar results (Supplementary Data 3). These analyses link the upregulated IR with PCa stemness.

We additionally re-analyzed three recently published datasets that examined differentiation of different SC systems and also observed a positive correlation between IR and normal stemness. Hence, in genetically matched hESCs–fibroblasts–iPS–fibroblasts system^29^, ESCs lost IR during fibroblast differentiation while fibroblasts regained IR when they were reprogrammed to iPS cells (Supplementary Fig. 5a), in line with the earlier report^30^. During spermatogenesis, spermatocytes displayed higher levels of IR than differentiated spermatids (Supplementary Fig. 5b) and these IR events were enriched in genes associated with gamete function^31^. Finally, IR was found to be prevalent in stem-like, resting CD4^+^ T cells vs. functionally activated (differentiated) counterparts (Supplementary Fig. 5c), as reported previously^32^. Significantly, overlapping of IR-affected genes identified in CRPC-Ad (vs. pri-PCa) with those in basal (vs. luminal), hESCs (vs. eFibroblasts), and resting (vs. activated) CD4^+^ T cells revealed a steep increase in the number of shared genes (Supplementary Fig. 5d-f). In addition, IR exhibited a PCa stage-specific pattern (Supplementary Fig. 5g). These analyses, together with the functions of many shared IR-affected genes (Supplementary Note 1), further support a functional role of IR in conferring PCa stemness and aggressiveness.

We next investigated the splicing code of IR^33^ in an attempt to understand the molecular basis of preferential IR in aggressive PCa. Retained introns in normal tissues generally have weak 5′ and 3′ splice sites^33^. Surprisingly, the splice site strength analysis did not reveal weak 5′ or 3′ sites in retained introns in pri-PCa, CRPC-Ad, and CRPC-NE—in fact, CRPC-Ad showed stronger splice sites than pri-PCa (Supplementary Fig. 6a). Sequence feature analysis indicated that, compared with constitutive introns, IR in pri-PCa preferred introns with less GC content (GC%) and longer sequence length, whereas CRPC-Ad specifically retained introns that were generally shorter without a difference in GC% (Supplementary Fig. 6b). No feature variation was observed in the retained introns in CRPC-NE vs. CRPC-Ad (Supplementary Fig. 6b). Together, these results suggest that the prevalence of IR in PCa is not associated with weak splice sites and may largely be *trans*-regulated.

To determine the potential *trans* factors (i.e., SRGs) that may preferentially regulate IR, we performed motif search for 95 RNA-binding proteins (RBPs) with known consensus motifs^34,35^ on differentially splicing introns compared with constitutive introns. Based on an RBP-binding score for each factor, we chose top 20 genes for further analysis. As shown in Fig. 2e, we identified a few genes that may preferentially regulate IR for each specific comparison. Nonetheless, the majority of RBPs were shared by introns regardless of the IR status, suggesting that the spliceosome functions as a group rather than that one particular factor preferentially regulates one AS type. In support, we decoded AS events associated with gene expression abundance by fractionating a cohort into two extremes (Fig. 2f). As expected, although the expression of ELAVL1 in pri-PCa and RBM38 in CRPC-Ad cohorts, respectively, both dramatically impacted IR, other splicing types were affected as well (Fig. 2f). Interestingly, ELAVL1 was not dysregulated in pri-PCa vs. N (fold change (FC) = 1.1). The discrepancy between a potential IR-inhibiting function of ELAVL1 and a marked increase in IR implied an involvement of other SRGs in preferential (or balanced) regulation of IR in pri-PCa. On the other hand, an IR-inhibiting function of RBM38 was consistent with its downregulation (FC = 2.3) and with increased IR in CRPC-Ad vs. pri-PCa (Fig. 2f). A TS role has been reported for RBM38^36^.

Subsequently, we interrogated potential biological impact of the upregulated IR on PCa biology. IR in normal conditions usually causes nonsense-mediated RNA decay (NMD) to downregulate gene expression^37^. We compared the bulk RNA levels of IR-affected genes using two different mathematical methods and found that, surprisingly, these genes generally exhibited higher expression than their constitutively spliced counterparts (Fig. 2g and Supplementary Fig. 6c). To further strengthen our finding, we overlapped the IR-affected genes with a high-confidence set of human NMD targets^38^ and found that only ~10% of genes in all groups were potentially targeted by NMD, although the genes with upregulated IR in CRPC tended to have slightly higher percentage (Fig. 2h; two-tailed *χ*^2^-test). These results indicate that IR in PCa minimally causes NMD-mediated downregulation and these IR-bearing genes are thus likely functional. In support, GO analysis of IR-affected genes revealed that, in addition to commonly observed category of “splicing and RNA metabolism,” several distinct categories were enriched in aggressive PCa (Supplementary Fig. 6d-f). For example, GO terms “stress response,” “DNA repair,” and “cancer-related signaling” (e.g., ERBB, NOTCH, and WNT) were unique to CRPC-Ad (Supplementary Fig. 6e), whereas “hormone transport” and “SC & development” were strongly associated with androgen-insensitive and CSC-enriched CRPC-NE (Supplementary Fig. 6f). Moreover, density plot analysis showed that, although the average ΔPSI value for upregulated IR in pri-PCa, CRPC-Ad, and CRPC-NE were 0.143, 0.18, and 0.15, respectively, the majority of these events could be validated by reads coverage (Supplementary Fig. 6g). This was consistent with reports that the ΔPSI values for cancer-related IRs are moderately low^9^. Sequence length and feature analysis indicated that many of the retained introns might have peptide-coding potential (Supplementary Fig. 6h and Supplementary Note 1), implicating functional relevance of the IR in PCa.

### AR regulates a splicing program distinct from transcription

**Fig. 3:**
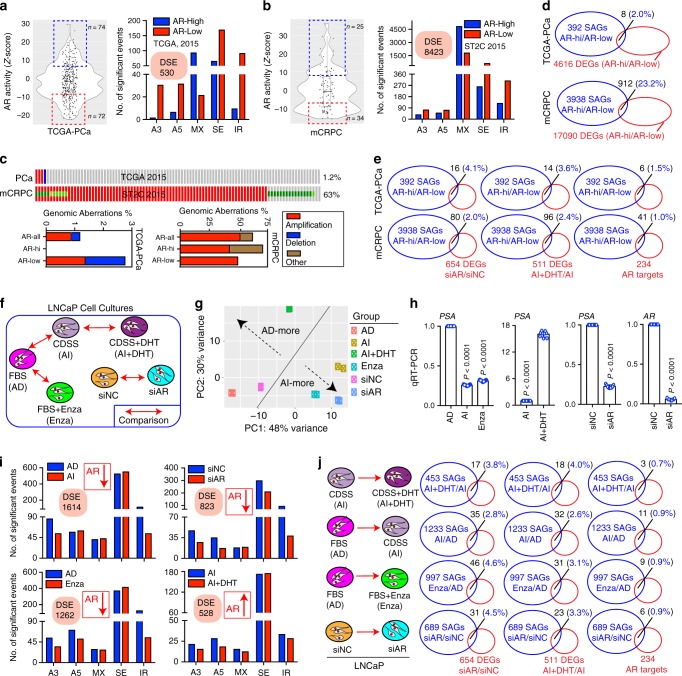
AR activity impacts AS landscape distinctively from its transcriptional regulation. **a**, **b** DSEs associated with high and low AR activity (cutoff, *Z*-score > 7 (blue dashed box) or < −7 (red dashed box)) in pri-PCa (**a**) and CRPC-Ad (**b**), respectively. AR activity (see Methods) was used to fractionate patient cohorts followed by splicing analysis by rMATS. Within the violin plots, the center lines represent median values, box edges are 75th and 25th percentiles, and whiskers denote the maximum and minimum values, respectively. **c** Genomic alterations do not contribute to the diversity of AR activities across PCa populations. Shown are frequency and types of AR mutations observed in TCGA and CRPC cohorts. AR activities of samples were grouped as in **a**. **d**, **e** Overlap between SAGs and DEGs (**d**), and between SAGs and three sets of AR-regulated genes (**e**) in indicated contexts. The number in parentheses denotes percentage of overlapped genes  proportional to all SAGs. Circles are not drawn to scale. **f**–**h** Experimental design (**f**), principal component analysis (PCA) showing proper clustering of samples (**g**), and qPCR validation of intended modulations of AR signaling in LNCaP cells (**h**). In **h**, the error bars represent the mean ± SD (*n* = 9). The *P*-value was calculated using two-tailed unpaired Student’s *t*-test. **i** The AR-regulated AS program in PCa cells. Shown are the DSEs associated with high (up arrow) or low (down arrow) AR activity in LNCaP cells detected by rMATS. **j** Overlap between SAGs and three sets of AR-regulated genes in indicated contexts. The number in parentheses denotes percentage of overlapped genes  proportional to all SAGs. Circles are not drawn to scale. A3, alternative 3′ splice sites; A5, alternative 5′ splice sites; DEGs, differentially expressed genes; DSEs, differentially spliced events; IR, intron retention; MX, mutually exclusive exons; SAGs, splicing-affected genes; SE, exon skipping.

AR is obligatory for pri-PCa growth and continues to be expressed and functionally important in CRPC^39^. ADT promotes a stem-like phenotype in PCa^3^ and relapsed tumors often exhibit enhanced SC properties^6,25,27^. We set out to determine whether AR may drive splicing dysregulation seen in PCa evolution. We first established an AR activity score based on the *Z*-scores calculated from the expression of 20 AR transcriptional targets^16^. The TCGA cohort bearing “uninterrupted” intrinsic AR heterogeneity^16^ and CRPC-Ad cohort bearing “twisted” AR activity as a result of treatments^19^ were then fractionated into high and low AR-activity groups, followed by splicing analyses. Not surprisingly, primary tumors with low vs. high AR activities displayed a significant difference in AS landscape (Fig. 3a) and this difference was amplified in CRPC (Fig. 3b), implicating AR signaling in modulating global AS. Of note, we observed no association between AR genomic alterations and its potential splicing-modulating activity (Fig. 3c), as AR is rarely altered in pri-PCa but frequently amplified in mCRPC^19^. To assess the impact of AR-associated splicing on AR-regulated gene expression, we compared the SAGs with DEGs identified in the AR-low vs. AR-high comparisons. Surprisingly, only 2% of SAGs overlapped with the DEGs in pri-PCa, although this overlap was increased to 23% in CRPC-Ad (probably due to an enlarged repertoire of AR-regulated events in CRPC) (Fig. 3d). Thus, AR activity-associated AS events exerted a limited impact on AR transcriptional targets, leading us to hypothesize that AR controls a splicing program distinct from its transcriptional regulation. In support, when we extended the comparison with three other well-defined AR-target gene sets^40^ (and two in this study, see below), we observed generally <4% overlaps across all comparisons (Fig. 3e).

To experimentally validate our hypothesis, we treated AR^+^ LNCaP cultures with various regimens to modulate AR activity (Fig. 3f). Cells cultured in regular fetal bovine serum (FBS)-containing medium represent an androgen-dependent (AD) state. Cells grown for 4 days in medium containing charcoal/dextran-stripped serum (CDSS) or treated with Enza (10 μM) were considered androgen-independent (AI). We also utilized siRNA to silence endogenous AR. Finally, cells primed with CDSS for 3 days were treated with 10 nM dihydrotestosterone (DHT) for 8 h to restore AR signaling. Deep RNA-seq was performed in biological duplicates on abovementioned LNCaP cultures (Fig. 3f and Supplementary Fig. 7a). Principal component analysis indicated that samples were properly clustered (Fig. 3g) and AR signaling was effectively modulated as intended, evidenced by levels of AR and prostate-specific antigen (PSA), and by GSEA of AR gene signature (Supplementary Fig. 7b) and by quantitative reverse-transcription PCR (qRT-PCR) analysis (Fig. 3h). Pairwise comparisons uncovered significant differences in DSEs in cells exhibiting high vs. low AR activities (Fig. 3i and Supplementary Fig. 7c). Also, re-analysis of a recent RNA-seq dataset (GSE71797)^41^ confirmed that in response to R1881 (24~48 h), activated AR signaling reshaped the AS landscape in three AR^+^ PCa cell models (i.e., LNCaP, VCaP, and 22Rv1) (Supplementary Fig. 7d). Similarly, by categorizing the DEGs identified in cells depleted of AR (siAR vs. siNC) or treated with DHT as AR-target sets, we found that, strikingly, these two sets, together with a previously reported AR signature^40^, minimally overlapped with the SAGs (<5%) defined in all different contexts (Fig. 3j and Supplementary Fig. 7e). Collectively, we conclude that AR regulates a set of AS-bearing genes distinct from its transcriptional targets, with or without the presence of androgen.

We also investigated whether AR might specifically regulate IR, as tumors and basal cells with low canonical AR activity were associated with increased levels of IR (Fig. 2c). Surprisingly, our work in LNCaP system revealed that a decrease in AR activity resulted in no increase, but a decrease, in IR while stimulation of AR-mediated transcription failed to appreciably repress IR (Supplementary Fig. 7f). To further test this experimentally, we utilized a quantitative reporter system^42^ in which a chimeric β-globin/immunoglobulin intron was inserted into the firefly luciferase gene (Supplementary Fig. 7g). Dual-luciferase assays indicated that consistent with previous reports^42^, splicing conferred an advantage to gene expression in that equal amounts of transfected plasmids generated higher signals from intron-containing than intronless luciferases (Supplementary Fig. 7h, left). However, luciferases with or without intron generated a similar pattern of signal changes across conditions with dampened or enhanced AR signaling (Supplementary Fig. 7h, right). We further replaced the β-globin intron with the intron 3 of AR-target gene *PSA* (Supplementary Fig. 7i) in which an IR event of *PSA* intron 3 has been reported in CRPC^20^ and observed similar pattern as that of β-globin intron (Supplementary Fig. 7j). These results suggest that AR does not specifically regulate IR in PCa cells.

### Genomic alterations in SRGs impact AS and PCa aggressiveness

**Fig. 4:**
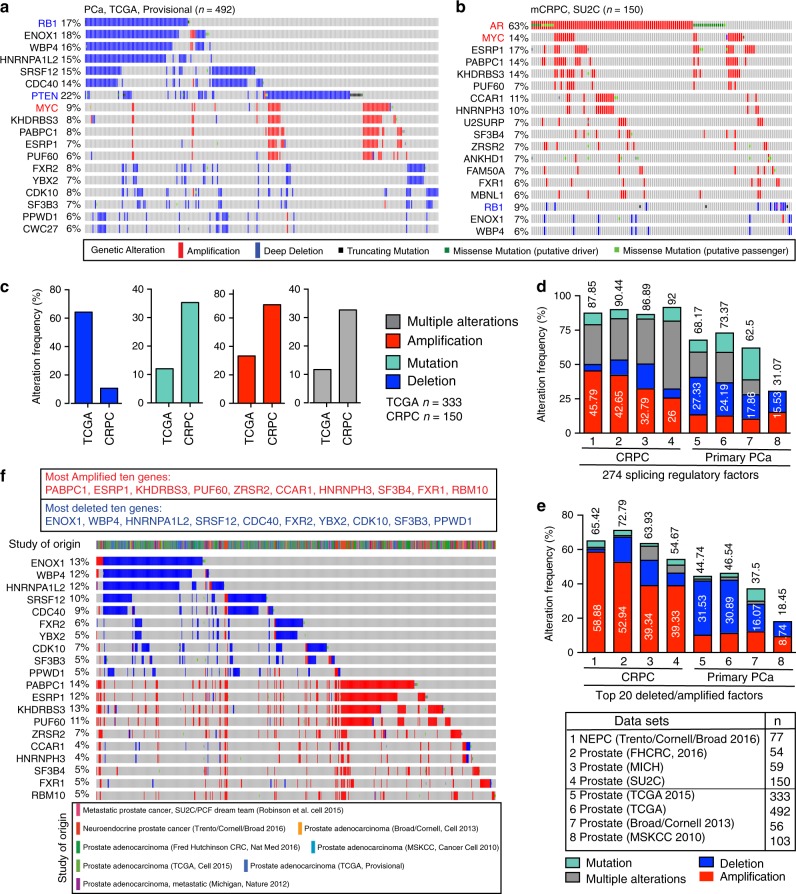
SRGs are frequently deleted and amplified in pri-PCa and CRPC, respectively. **a**–**c** A comprehensive survey of genomic alterations in 274 SRGs in available clinical cohorts in cBioportal. The top 15 mutated SRGs are shown in the representative pri-PCa (**a**) and metastatic CRPC-Ad (**b**) cohorts. Frequently deleted *RB1* and *PTEN* (colored in blue), and amplified *MYC* and *AR* (colored in red) genes are included as reference genes. Each bar represents the alteration status of an individual gene for a single patient and the percentage of alterations for each gene in the indicated cohort is provided. Shown in **c** are bar graphs summarizing the cumulative genomic alterations of SRGs in the largest and representative pri-PCa (TCGA) and CRPC (SU2C) cohorts. **d**, **e** Bar plots illustrating the cumulative aberration frequencies of all 274 SRGs combined (**d**) and the top 20 mutated SRGs (10 most amplified and 10 most deleted) (**e**) across all cohorts, with numbers above and within the bars representing the total frequency and the frequency of amplification or deletion of indicated genes, respectively. **f** Integrated mutational landscape of top 20 mutated SRGs in PCa showing mutual exclusivity, in large part, between deletions and amplifications of SRGs. See Supplementary Data 1 for details.

Recent genomic sequencing has revealed global mutational landscapes of PCa during development and progression^4,16,19,39,43–47^, almost all of which focused their analysis on known PCa-related genes and pathways (e.g., AR, PTEN/PI3K, TP53, RB1, DNA repair, ETS fusion), whereas alterations in SRGs were overlooked due to a low mutation frequency at individual gene level. Moreover, point mutations in spliceosome core genes have been recognized as a key driver in hematological cancers^8^. We explored the molecular mechanisms underpinning the AS dysregulation in PCa by compiling and curating a catalog of 274 SRGs (Supplementary Data 1) and systematically surveying their mutational landscape (Fig. 4 and Supplementary Figs. 8–10). We interrogated 11 available large-scale clinical datasets in cBioportal^48^ and excluded 3 from further analysis due to limited information available (Supplementary Data 1). The remaining eight were categorized as pri-PCa and CRPC datasets. Figure 4a, b showed the mutational landscape of top 15 altered SRGs in representative pri-PCa and CRPC datasets (also see Supplementary Figs. 8 and 9), respectively. Several interesting patterns emerged. First, genomic deletions of SRGs in pri-PCa and amplifications in CRPC represented the most prevalent alterations (Figs. 4c, d). Second, the frequently deleted and amplified genes often co-occurred with the deletion of TS genes and amplification of oncogenes, respectively (Supplementary Data 1). For example, *ENOX1*, *WBP4*, *HNRNPA1L2*, and *RB1* were colocalized and co-deleted on Chr13q (*p* = 5.16E − 42, one-sided Fisher’s exact test; Supplementary Fig. 10a). On the other hand, *KHDRBS3*, *PABPC1*, *ESRP1*, and *PUF60* were co-amplified with *MYC* on 8q. Third, most SRGs were mutated at low frequency, as only 20 (7.3%) and 29 (10.6%) of the 274 SRGs were mutated at a rate of ≥5% in TCGA-PCa and SU2C-CRPC cohorts, respectively. Consequently, the mutation burden in sum is predominantly contributed by the top 20 altered genes (Fig. 4e). Fourth, chromosomal distribution of mutated SRGs (≥5%) showed that, except for the top altered genes, the majority of SRGs were localized outside the previously reported hotspots^16,47^ (Supplementary Fig. 10b), in line with their low mutation rates. In aggregate, our data indicate that, albeit a low alteration frequency at individual gene level, SRGs collectively represent a frequently mutated pathway in PCa, as ~31–68% and 87–94% of patients with pri-PCa and CRPC, respectively, harbor at least one mutation of one SRG (Supplementary Data 1).

Evolutionarily, deletion and amplification of selective SRGs might represent early and late events, respectively, in PCa pathogenesis (Fig. 4c). Group analysis of top altered SRGs showed that deletion of SRGs did not, whereas amplification of SRGs did, associate with increased Gleason grade (Supplementary Fig. 8e), highlighting a potential survival advantage of clones harboring SRG amplifications over deletions during PCa progression. This notion is further supported by a recent study showing that focal genomic amplifications represent a rapid adaptation to selection pressure and a driving force in mCRPC^49^. We also observed an overall increase (e.g., 8% to 14% for *KHDRBS3*, 7–17% for *ESRP1*) and decrease (e.g., 18–7% for *ENOX1*, 16–6% for *WBP4*) in the frequencies of amplified and deleted genes, respectively, in CRPC vs. pri-PCa (Fig. 4a, b). Interestingly, SRG deletions and amplifications seemed to be mutually exclusive (Fig. 4f).

**Fig. 5:**
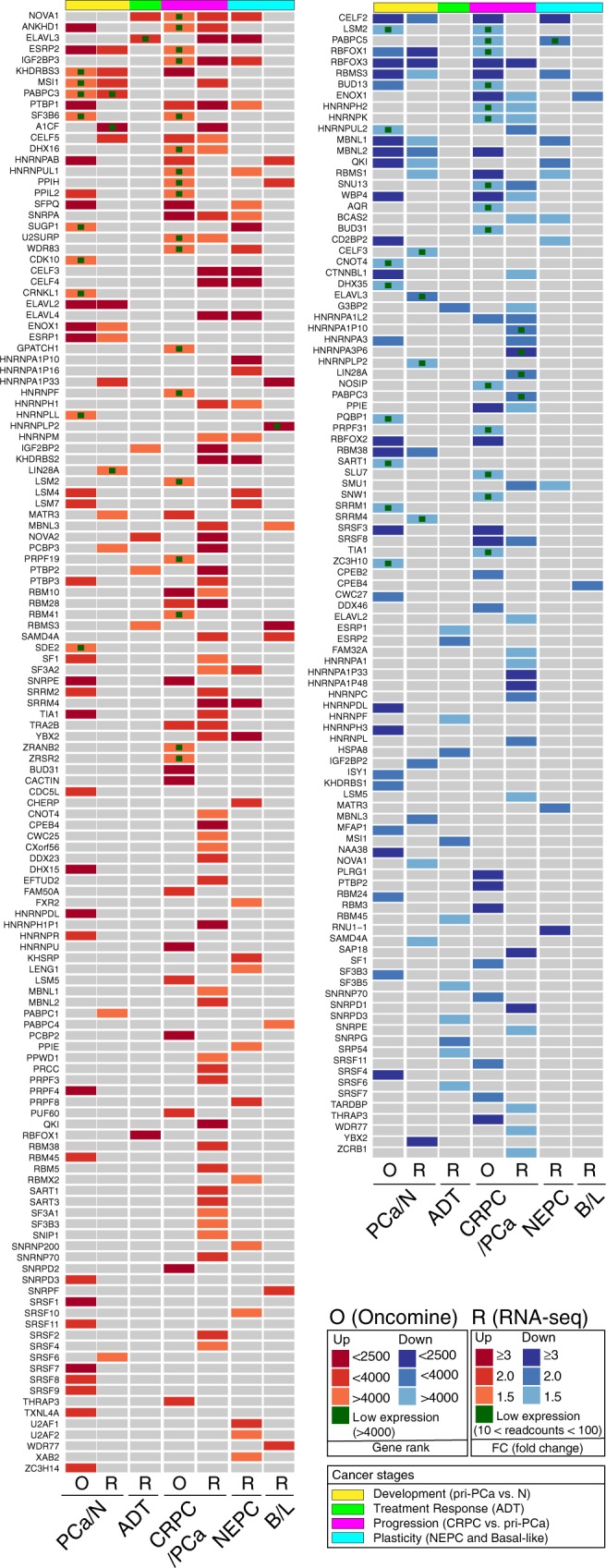
Mis-expression of SRGs in PCa. Integrated heatmap of differentially expressed SRGs identified in Oncomine (*p* < 0.05) and RNA-seq (fold change (FC) ≥ 1.5 and FDR < 0.1 calculated by DESeq2 v.1.18.1). In Oncomine (O), the medium-rank of <2500, <4000, and >4000 for a gene denotes high, moderate, and low levels of expression, respectively. The rank for a gene is the median rank for that gene across each of the analyses. The *p*-value for a gene is its *p*-value for the median-ranked analysis. For visualization, DEGs revealed by RNA-seq (R) data are categorized into three groups according to FC differences (FC ≥ 3, ≥2, and ≥1.5). Based on pairwise comparisons, the stages of PCa are defined as tumor development (pri-PCa vs. normal tissues), ADT treatment response (ADT-after vs. -before), CRPC progression (CRPC-Ad vs. pri-PCa), and plasticity (CRPC-NE vs. CRPC-Ad). Also see Supplementary Fig. 11 and 12, and Supplementary Data 4.

We reasoned that CNVs in SRGs might lead to their differential mRNA expression, which in turn might be tied to splicing misregulation in PCa. Indeed, gene expression analysis for top altered SRGs in both pri-PCa and CRPC indicated that deletion and amplification generally correlated with loss and gain of mRNA expression, respectively (Supplementary Fig. 11). Oncomine Concept analysis revealed 72 and 74 dysregulated SRGs in pri-PCa and CRPC, respectively (Supplementary Fig. 12a). In RNA-seq datasets, 33, 89, and 45 SRGs were deregulated in pri-PCa (vs. N), CRPC-Ad (vs. pri-PCa), and CRPC-NEPC (vs. CRPC-Ad), respectively (Supplementary Fig. 12b). Furthermore, an RNA-seq examining the response of advanced PCa to ADT^17^ revealed 19 DEGs and an exclusive overexpression of 7 genes was identified in basal vs. luminal cells (Supplementary Fig. 12b). Notably, many of the top amplified and deleted SRGs were also found to be, correspondingly, overexpressed and downregulated in PCa at the population level (Supplementary Fig. 12c). An integrated summary (Fig. 5 and Supplementary Data 4) revealed that, in total, 186 out of 274 (67.9%) SRGs were mis-expressed at different stages of PCa, with more dysregulated SRGs found in CRPC, implicating a potential dependency of aggressive PCa on spliceosome activity.

### SRGs are prognostic

**Fig. 6:**
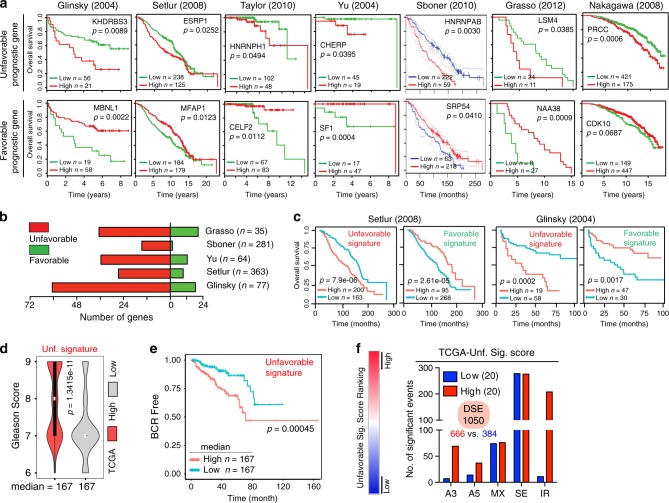
SRGs are prognostic and associated with splicing dysregulation. **a** Kaplan–Meier plots for representative unfavorable and favorable genes associated with patient overall survival in seven different cohorts. **b** Numbers of genes showing favorable and unfavorable prognostic effects in five distinct cohorts. Patient numbers for each cohort are shown in parentheses. **c** Meta-analysis showing a higher level of unfavorable signature (13 genes: *SRSF1*, *KHDRBS3*, *ESRP1*, *HNRNPH1*, *U2SURP*, *LSM5*, *TIA1*, *CHERP*, *HNRNPR*, *HNRNPH2*, *HNRNPH3*, *HNRNPAB*, and *KHDRBS1*, with each showing consistent unfavorable prognosis in ≥3 datasets) and a lower level of favorable signature (13 genes: *MFAP1*, *SF3A2*, *GPATCH1*, *XAB2*, *CELF2*, *SF3A1*, *SAP18*, *SRP54*, *PPIL2*, *SF1*, *MATR3*, *ELAVL4*, and *CDK10* with each showing consistent favorable prognosis in ≥2 datasets) correlating with reduced overall patient survival, respectively. Data were based on the Setlur and Glinsky studies. **d**, **e** Unfavorable signature is associated with higher Gleason score (**d**) and a higher level of unfavorable signature positively correlates with disease recurrence in TCGA cohort (**e**). In **d**, the center white dots represent median values, box edges are 75th and 25th percentiles, and whiskers denote the maximum and minimum values, respectively. Shown in **e** is biochemistry recurrence (BCR)-free survival. **f** DSEs associated with high or low expression level of unfavorable signature in primary PCa cohort. The *p*-value was calculated using two-tailed unpaired Student’s *t*-test (**d**) and log-rank test (**a**, **c**, **e**). See Supplementary Data 5 for details.

To further explore the clinical relevance of SRGs, we assessed the prognostic values of dysregulated SRGs in patient’s outcome. We systematically surveyed the 186 misregulated SRGs in 7 Oncomine datasets containing patient survival information and identified two types of “prognostic” SRGs: unfavorable genes whose higher expression correlated with poor patient survival and favorable genes whose higher expression correlated with better patient survival (Fig. 6a). In general, we observed a consistency between overexpressed SRGs and unfavorable prognostic genes, but not for downregulated genes and favorable prognostic genes (Supplementary Data 5). Interestingly, although different datasets revealed varying numbers of prognostic genes, more SRGs were classified as unfavorable genes (Fig. 6b). Together with the mutational landscape (Fig. 4) and deregulated expression patterns of SRGs (Fig. 5) that cooperatively indicated a potential dependency of CRPC on spliceosome activity, this would strongly suggest that SRGs mostly play oncogenic roles in PCa progression. Importantly, most of the identified prognostic genes have not previously been linked to PCa patient survival. Towards a better use of these prognostic SRGs in heterogeneous PCa, we established two gene signatures based on the consistency of the survival results seen in the seven datasets, corresponding to unfavorable signature and favorable signature (see Methods). We found that patients whose cancer gene expression enriched for the unfavorable or favorable signatures had a worse or a better survival outcome, respectively (Fig. 6c), suggesting a utility of SRGs as prognostic biomarkers. To further study the underlying link between prognostic SRGs and splicing dysregulation, we investigated the impact of unfavorable signature on disease aggressiveness and splicing in the TCGA cohort. As expected, the unfavorable signature score positively and negatively associated with the tumor grade and disease recurrence, respectively (Fig. 6d, e). Importantly, primary tumors expressing highly or lowly the unfavorable signature exhibited distinct splicing landscapes, with total DSEs (1.73-fold) and IR (18.91-fold) being specifically upregulated in the high group (Fig. 6f).

### SRG downregulation inhibits PCa cell aggressiveness and alters AS

**Fig. 7:**
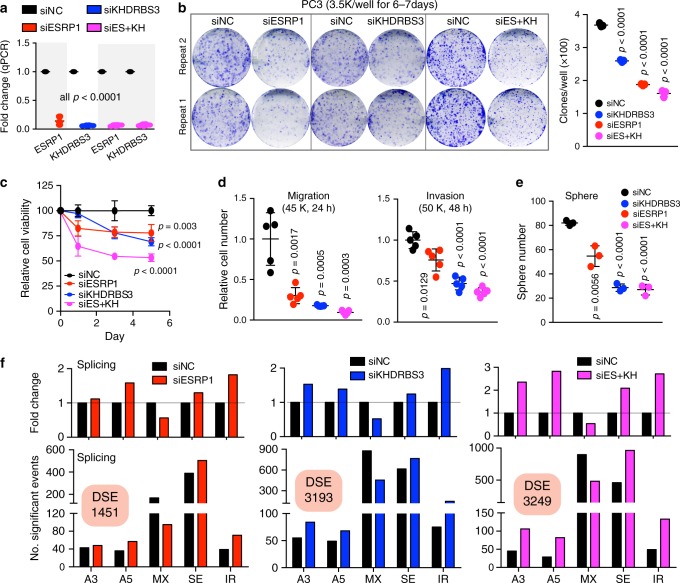
Modulating SRG expression impacts AS and aggressiveness of PCa cells. **a** qPCR analysis showing the knockdown efficiency of *ESRP1* and/or *KHDRBS3*. PC3 cells were treated with non-targeting siRNAs (siNC) or with siRNAs targeting *ESRP1* and/or *KHDRBS3* (20 nM; 72 h). Error bars represent the mean ± SD (*n* = 3). **b** Knocking down *ESRP1* or/and *KHDRBS3* inhibits clonal development in PC3 cells. Shown were representative images of two repeat experiments (left) and quantification data (right). Error bars represent the mean ± SD (*n* = 3). **c**–**e** Knocking down *ESRP1* or/and *KHDRBS3* inhibits proliferation (**c**), migration and invasion (**d**), and sphere formation (**e**) in PC3 cells. Cell proliferation (viability) was determined by MTT assays (**c**). Data represent the mean ± SD from a representative experiment with four technical repeats and the experiment was replicated two times with similar results. Migration and invasion were determined by counting cells of five random 10× fields (**d**) (mean ± SD; *n* = 5). Spheres were enumerated 9 days after plating (4000 cells/well) (**e**) (mean ± SD; *n* = 3). **f** RNA-seq analysis of PC3 cells depleted of ESRP1 and KHDRBS3, individually or in combination, by specific siRNAs. Shown are the relative fold changes (upper) and absolute number (bottom) of the identified DSEs (differentially spliced evets) in indicated contexts. siES + KH denotes double knockdown by siRNAs against ESRP1 and KHDRBS3. All *P*-values were calculated using two-tailed unpaired Student’s *t*-test. Source data are provided as a Source Data File.

To functionally demonstrate the role of SRGs, we knocked down ESRP1 and KHDRBS3 in PC3 cells, as both genes were amplified and overexpressed in pri-PCa and CRPC-Ad (Figs. 4a, b and 5, and Supplementary Data 4) and predictive of worse outcome in PCa patients (Fig. 6a). The splicing-regulatory functions for ESRP1 have been well-documented in other cancer types^23^ and a recent genomic study has linked ESRP1 to the aggressiveness of early-onset PCa^50^. The role of KHDRBS3 in PCa is unclear. siRNA-mediated depletion of ESRP1 or KHDRBS3 (Fig. 7a) inhibited clonal capacity (Fig. 7b), viability (Fig. 7c), migration and invasion (Fig. 7d), and sphere formation (Fig. 7e) in PC3 cells. Combined knockdown of both genes demonstrated stronger inhibitory effects than individual gene knockdowns (Fig. 7b–e). To explore whether the above inhibitory effects were caused by, at least partially, by SRG downregulation-mediated splicing modulation, we performed RNA-seq analysis in PC3 cells treated with different siRNAs. The results indicated that depletion of ESRP1 and/or KHDRBS3 significantly altered the global splicing landscape with decreased MX but increased SE and IR (Fig. 7f). Knockdown of KHDRBS3 generally showed stronger effects on AS changes than ESRP1 knockdown (Fig. 7f). Collectively, our data suggests that knocking down amplified and clinically relevant SRGs alters the global AS patterns and inhibits oncogenic properties of PCa cells.

### CRPC cells are sensitive to spliceosome inhibition in vitro

To test the hypothesis that spliceosome may represent a preferential CRPC dependency, we first analyzed the mutational profiles of SRGs in seven PCa cell lines with increasing aggressiveness and found that AR^+^, relatively indolent PCa cells tended to have more SRG deletions, whereas AR^−^, aggressive cells showed more SRG amplifications. In particular, LNCaP and PC3 cells resembled pri-PCa and CRPC, respectively, with respect to SRG mutation profiles (Supplementary Fig. 13a). We retrieved two large-scale RNA interference (RNAi) screening data (Novartis Project Drive^51^ and Broad Project Achilles^52^) and performed GSEA on ranked lists of essential genes. We observed that aggressive AR^−^ PCa lines exhibited a preferential enrichment on two splicing pathway signatures (Supplementary Fig. 13b). By contrast, AR signaling and MYC signatures were enriched in AR^+^ LNCaP and 22RV1 vs. AR^−^ DU145 cells, respectively (Supplementary Fig. 13c). These analyses support the postulate that AR^−^, AI PCa cells may be particularly dependent on the spliceosome activity.

**Fig. 8:**
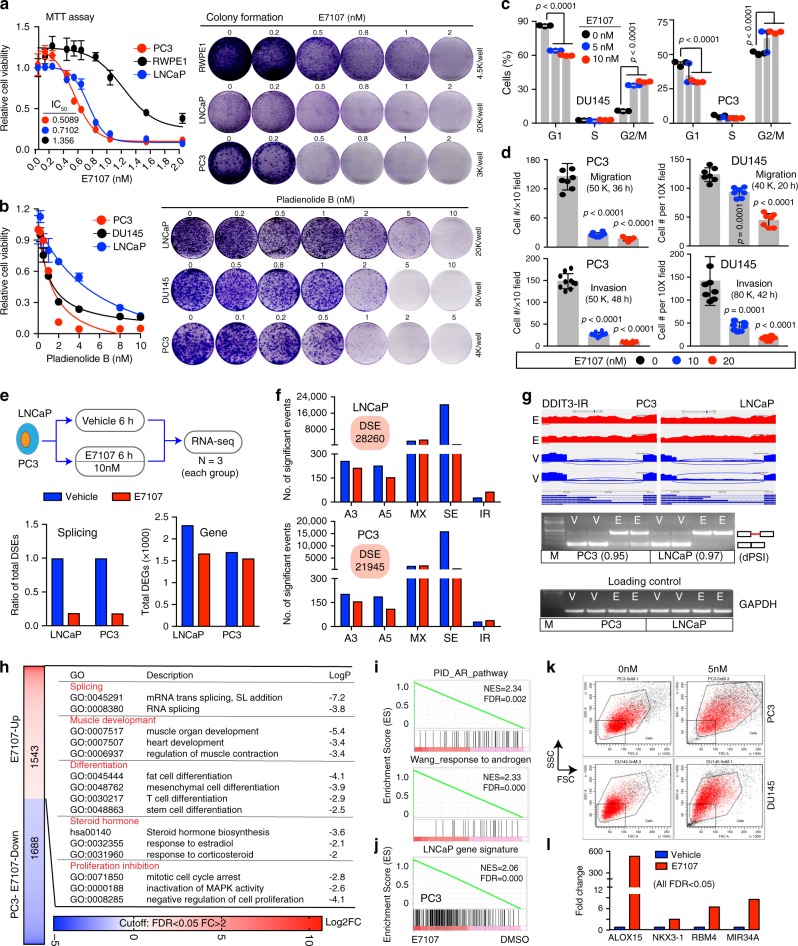
CRPC cells are sensitive to spliceosome inhibition. **a**, **b** Cell viability (MTT; left) and colony formation (right) assays in indicated cells treated with E7107 (**a**) or Pladienolide B (**b**). Data represent mean ± SD from a representative experiment with four technical repeats and the experiment was replicated three times with similar results. **c**, **d** Cell cycle (**c**) and migration and invasion assays (**d**) in E7107-treated PCa cells. For **c**, data represent mean ± SD from one representative experiment with three technical replicates and the experiment was repeated three times with comparable results. For **d**, data represent mean ± SD from cell counting of 5–10 low-magnification (10×) fields. For PC3 migration, *n* = 8, 8, 5 for 1, 10, 20 group, respectively. For DU145 migration, *n* = 7 for all groups. For invasion assays in PC3 and DU145, *n* = 10 and 8 for all groups, respectively. *P*-values were calculated using two-tailed unpaired Student’s *t*-test. **e** Effect of E7107 (10 nM) on PCa transcriptome in vitro. Shown are schematic of RNA-seq experiments (top) and total DSEs (bottom left) and DEGs (bottom right) upon E7107 treatment. **f** E7107 reshapes splicing landscape of PCa cells indicated. **g** Sashimi plot visualization and RT-PCR validation of IR in *DDIT3* gene after E7107 (10 nM, 6 h) treatment. For RT-PCR, three independent experiments were performed with two loaded in the gel (the sizes of *GAPDH* and the upper and lower bands of *DDIT3* mRNA were ~148, 459, and 192 bp, respectively). The event ΔPSI values calculated by rMATS were provided in parentheses. **h** GO analysis of genes upregulated in PC3 cells after E7107 (10 nM, 6 h). **i**, **j** GSEA showing enrichment of AR signatures (**i**) and LNCaP gene signature (defined as top 300 genes solely or overexpressed in LNCaP vs. PC3) (**j**) in E7107-treated PC3 cells. **k** Representative FACS plots of PC3 and DU145 cells treated with E7107 (5 nM; 3 days) showing increased cell size (*n* = 3). **l** Upregulation of tumor suppressors (*ALOX15*, *KNX3-1*, *RBM4*, and *MIR34A*) in PC3 cells after E7107 treatment. Data represent fold changes measured by RNA-seq. Source data are provided as a Source Data file.

We subsequently tested this postulate using spliceosome inhibitors. Several microbial products, including Pladienolide B and its derivative E7107, have been shown to bind and specifically inhibit the SF3B1 complex and manifest anti-cancer activities^8,13^. The E7107 compound represented the first-in-class spliceosome inhibitor that underwent phase I clinical trial^53^. We found that PCa cells exhibited preferential sensitivity to E7107 relative to non-tumorigenic prostate epithelial cells RWPE1, with PC3 being more sensitive than LNCaP cells (Fig. 8a and Supplementary Fig. 13d). Experiments with Pladienolide B confirmed PC3 as the most sensitive line (Fig. 8b). Although a long-term E7107 treatment (6~7 days) induced massive cell death (Fig. 8a and Supplementary Fig. 13d), shorter treatments (<3 days) generally elicited limited apoptosis but instead arrested PCa cells at the G2/M phase of the cell cycle (Fig. 8c). Treatment of PCa cells with E7107 for 20~48 h also inhibited cell migration and invasion, as measured by both Boyden chamber (Fig. 8d and Supplementary Fig. 13e) and scratch-wound (Supplementary Fig. 13f) assays. Importantly, treatment of PCa cells with 5 nM E7107 for 6 h dramatically reshaped the splicing pattern of the selected genes (Supplementary Fig. 13g), suggesting an on-target effect of the drug.

### E7107 molecularly reverses PCa cell aggressiveness

To uncover the mechanisms of action of E7107 in PCa, we treated LNCaP and PC3 cells with the drug for 6 h followed by RNA-seq analysis (Fig. 8e). No gross defects were observed in cell growth (Supplementary Fig. 14a) but, as expected, E7107 dramatically inhibited the AS globally in both cell types (Fig. 8e) with SE being the major type affected (Fig. 8f). Sashimi plot visualization of the sequencing data and RT-PCR validated splicing analysis (Fig. 8g and Supplementary Fig. 14b). GO analysis of the top 1000 genes with significant SE events inhibited by E7107 in PC3 cells revealed many GO terms associated with cancer-promoting functions, e.g., cell cycle and proliferation, DNA repair, splicing, and cancer pathways (Supplementary Fig. 14c and Supplementary Data 6), suggesting that E7107 inhibits splicing of a subset of PCa-promoting genes. At the gene expression level, E7107 reshaped the transcriptomes and exhibited a slight suppressive effect, especially in LNCaP cells, on transcription (Fig. 8e and Supplementary Data 7). qRT-PCR analysis validated DEGs identified in RNA-seq (Supplementary Fig. 14d).

We also performed GO analysis of DEGs upregulated after E7107 treatment. In AR^+^p53^+^ LNCaP cells, four main categories of related GO terms were identified (Supplementary Fig. 14e) with “Splicing” being the most significant one, consistent with a recent report^54^. AR and AR signaling were not significantly affected by E7107 in LNCaP cells (Supplementary Fig. 14f). Interestingly, p53 was activated, along with several other TS genes including *RBM4*^54^ and *MIR34A*^55^ (Supplementary Fig. 14g). Consistently, GO terms “cell cycle arrest” and “differentiation” were enriched (Supplementary Fig. 14e). We have previously shown that the LNCaP gene expression profile resembles that in pri-PCa^5^. GSEA of gene signatures specific to normal prostate tissues vs. pri-PCa revealed that the normal, but not the tumor, gene signature was significantly enriched in E7107-treated LNCaP cells (Supplementary Fig. 14h), suggesting a reversion of LNCaP transcriptome from PCa-like to normal-like. Similarly, pathway analysis in AR^−^p53^−^ PC3 cells identified both convergent (e.g., splicing, differentiation, cell cycle arrest and proliferation inhibition) and unique (i.e., steroid hormone and muscle development) GO categories, when compared with the analysis in LNCaP cells (Fig. 8h). Enrichment of “differentiation” and “steroid hormone” categories in PC3 cells prompted us to examine the androgen/AR signaling. Strikingly, transcript levels of *AR* and many typical AR targets were upregulated in PC3 cells treated with E7107, leading to a dramatic enrichment of AR pathway (Fig. 8i). Furthermore, a LNCaP gene signature was enriched in E7107-treated PC3 cells (Fig. 8j). Experimentally, E7107 treatment increased cell size in both PC3 and DU145 cells (Fig. 8k), indicating morphological differentiation. Moreover, although p53 was not activated in PC3 cells due to its genetic loss, several other TS genes were upregulated (Fig. 8l). These data, together, suggest a reversal, molecularly and phenotypically, of aggressive PCa cells (PC3) to a more indolent, LNCaP-like cell state upon spliceosome inhibition by E7107.

### Spliceosome inhibition therapeutically targets CRPC in vivo

**Fig. 9:**
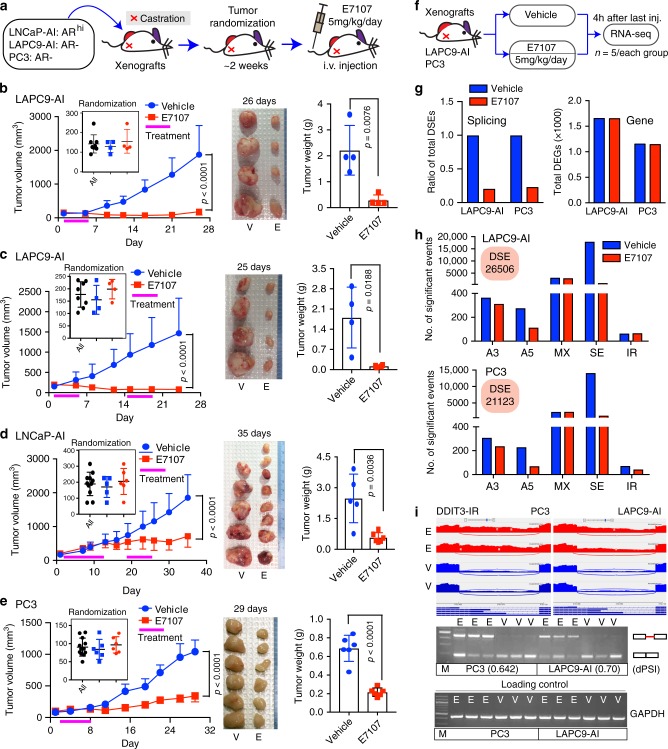
Therapeutic targeting of CRPC in vivo. **a** Schematic of in vivo E7107 treatment. **b**–**e** Inhibitory effects of E7107 on the growth of indicated Enza-resistant CRPC models in vivo. Shown are the tumor growth curves (left; insets present tumor randomizations), endpoint tumor images (middle), and tumor weight (right) of LAPC9-AI (**b**, **c**; *n* = 4 for each group), LNCaP-AI (**d**; *n* = 5 and 8 for vehicle and treatment group, respectively), and PC3 (**e**; *n* = 6 for each group) models treated with vehicle or E7107. Data represent mean ± SD and all *P*-values were determined by two-tailed unpaired Student’s *t*-test. **f**, **g** Effect of E7107 on CRPC transcriptome in vivo. Shown are schematic of RNA-seq experiment (**f**) and the ratio of total DSEs (**g**; left) and total DEGs (**g**; right) identified upon E7107 treatment in indicated CRPC models. **h** AS pattern showing that E7107 reshapes the splicing landscape of CRPC xenografts in vivo. **i** Sashimi plots and RT-PCR validation of IR in *DDIT3* gene after E7107 treatment in vivo. For RT-PCR, three independent experiments were performed with two loaded in the gel. The event ΔPSI values calculated by rMATS were provided in parentheses. E, E7107; V, vehicle; M, DNA marker. In the gel images, the sizes of *GAPDH* and the upper (with intron retention) and lower bands of *DDIT3* mRNA were 148, 459, and 192 bp, respectively. Source data are provided as a Source Data file.

We treated three distinct castration-resistant (AI) PCa xenograft models, i.e., the AR^+/hi^ LNCaP-AI^25^, AR^−/lo^ LAPC9-AI^25^ and AR^−^ PC3, with E7107 or vehicle (Fig. 9a). The LNCaP-AI and LAPC9-AI models were established by serially passaging the corresponding parent AD tumor cells in castrated immunodeficient mice^25^. The LNCaP-AI was initially responsive to Enza but quickly became Enza-resistant, whereas LAPC9-AI was refractory to Enza de novo^25^. Treatment of LAPC9-AI tumors with either one cycle (i.e., tail vein injection for 5 consecutive days) or two cycles (with 1 week of drug holiday between the 2 cycles) effectively inhibited tumor growth (Fig. 9b, c and Supplementary Fig. 15a, b, left). Similarly, treatment of mice bearing AR^+/hi^ LNCaP-AI with two cycles of E7107 (Fig. 9d and Supplementary Fig. 15c, left) and PC3 xenografts with one cycle of E7107 (Fig. 9e and Supplementary Fig. 15d, left) also inhibited tumor growth. Although a certain degree of toxicity of E7107 was observed, treated mice returned to normal body weight within a week after cessation of treatment (Supplementary Fig. 15a-d, right). The endpoint tumors frequently displayed a more differentiated morphology manifested by enlarged and polynucleated cells (Supplementary Fig. 15e).

To determine whether anti-tumor effects of E7107 are associated with spliceosome inhibition, we performed RNA-seq in LAPC9-AI and PC3 tumors 4 h after the fifth injection of E7107 (Fig. 9f). Consistent with in-vitro data (Fig. 8e), E7107 suppressed the AS globally in both AR^−/lo^ CRPC models (Fig. 9g), evidenced by decreased A3, A5, and SE (Fig. 9h). Sashimi plot visualization and RT-PCR validated our splicing analysis (Fig. 9i and Supplementary Fig. 15f). GO analysis of the top 1000 genes with downregulated SE events in LAPC9-AI model upon E7107 treatment revealed cancer-promoting GO categories including “cell cycle and proliferation,” “DNA repair,” “splicing,” and “cancer pathways” (Supplementary Fig. 15g). Analysis of expression changes after E7107 treatment revealed 3299 and 2289 DEGs, respectively, in LAPC9-AI and PC3 systems without obvious bias on transcription (Fig. 9g).

qRT-PCR analysis in tumor samples confirmed the differential expression of selected genes (Supplementary Fig. 16a). GO analysis of the genes upregulated in E7107-treated LAPC9-AI tumors revealed a broad spectrum of functional categories linked to inhibition of cell proliferation and of developmental, differentiation, inflammation, and TS pathways, among others (Supplementary Fig. 16b). As the LAPC9-AI has the AR^−/lo^ phenotype^25^, transcription of AR signaling remained unaltered (Supplementary Fig. 16c). Notably, gene signatures specific to pri-PCa and CRPC were enriched in E7107- vs. vehicle-treated LAPC9-AI tumors, respectively (Supplementary Fig. 16d), again suggesting that spliceosome inhibition by E7107 reverses the gene expression pattern of LAPC9-AI from CRPC-Ad-like (aggressive) to pri-PCa-like (indolent). We have recently shown that LAPC9-AI molecularly resembles CRPC-Ad^25^. In the PC3 model, we observed an increase in expression of genes involved in inflammation, immune cell infiltration and androgen response, among others, after E7107 treatment (Supplementary Fig. 16e). Interestingly, despite the upregulated category of “androgen response,” AR signaling and many targets remained inactivated (Supplementary Fig. 16f). Compared with in-vitro data showing that E7107 strongly boosted the AR signaling (Fig. 8i), this discrepancy could potentially be explained by an in vivo environment lacking androgen in castrated hosts such that the “E7107 reprogramed” AR^+^ PC3 cells may not survive. Treated endpoint PC3 tumors tended to be less aggressive in terms of molecular signatures (Supplementary Fig. 16g-i). For instance, E7107 inhibited pathways associated with cancer metastasis and stemness (Supplementary Fig. 16g), decreased the expression of a PC3-cell signature (Supplementary Fig. 16h) and reverted the gene expression pattern from CRPC-NE-like to CRPC-Ad-like (Supplementary Fig. 16i). We have previously demonstrated that PC3 cells molecularly resemble the CRPC-NE^5,25^.

### E7107 inhibits the progression in transgenic Hi-Myc PCa model

**Fig. 10:**
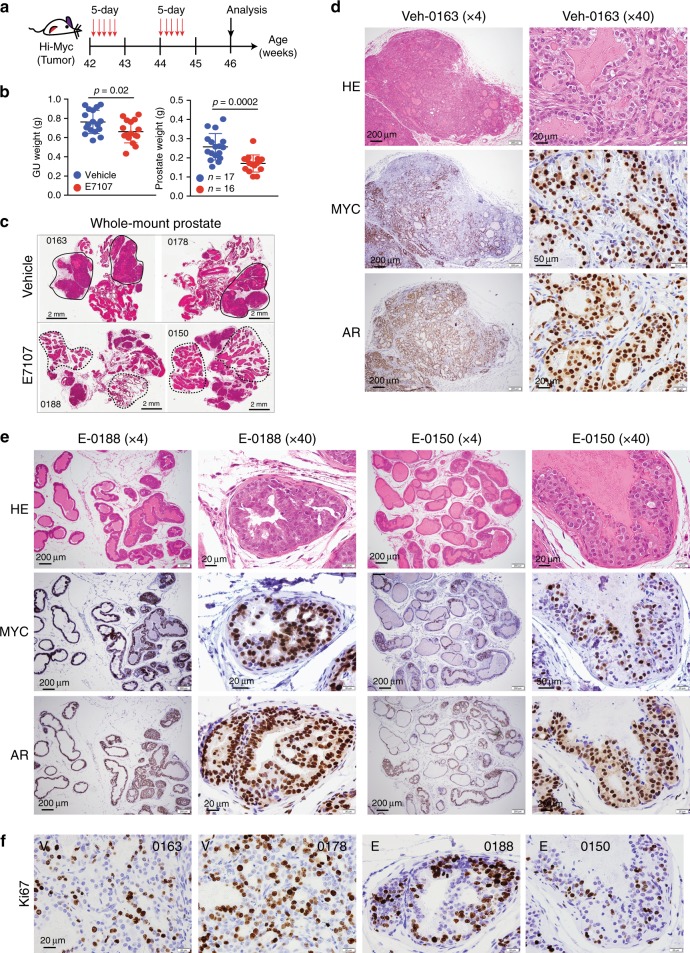
E7107 inhibits Hi-Myc PCa in vivo. **a** Schematic of in vivo E7107 treatment (5 mg/kg/day) in the Hi-Myc PCa model (see Methods). **b** Weight of the genitourinary (GU) tract (left) or the prostate (right) in Hi-Myc mice treated with vehicle or E7107. *P*-values were determined by two-tailed unpaired Student’s *t*-test. **c** Representative whole-mount Aperio Scanscope images of hematoxylin and eosin-stained prostate sections from two Hi-Myc mice (animal tags indicated) treated with vehicle (top) or E7107, respectively. Solid circled areas mark Hi-Myc tumors in the vehicle group (top), whereas dashed circled regions highlight prominent benign and hyperplastic glands in the E7107-treated group (bottom). **d**, **e** Histological analysis of the vehicle (**d**) and E7107-treated (**e**) Hi-Myc tumors collected at 46 weeks. Shown are representative HE and MYC, and AR IHC images at low (×4) and high (×40) magnifications. Five tumors were analyzed for each group. Scale bars are also indicated. **f** Representative images of Ki67 staining in the control (0163 and 0178) and E7107-treated (0188 and 0150) Hi-Myc tumors. Original magnifications, ×40. Scale bar for all panels, 20 μm. Source data are provided as a Source Data file.

As Myc overexpression and function represent a critical early oncogenic driver of PCa, many SRGs are co-amplified with the *MYC* gene and Myc-driven lymphomas are susceptible to spliceosome interference^10^, we treated the Myc-driven murine PCa (Hi-Myc tumors) with E7107 (Fig. 10a) and observed significant inhibition by E7107 of Hi-Myc tumors (Fig. 10b). Histological examination of whole-mount images revealed large areas of Ads in vehicle-treated prostates that were frequently fused (Fig. 10c; top, solid circles). In contrast, most E7107-treated Hi-Myc prostates contained reduced tumor areas and prominent benign and hyperplasic glands (Fig. 10c, bottom, dashed circles and Fig. 10d, e). Immunohistochemistry (IHC) analysis revealed that the benign/hyperplastic glands in E7107-treated animals expressed AR and MYC to similar levels in the vehicle-treated tumors (Fig. 10d, e). Compared with the vehicle-treated Hi-Myc tumors, the benign/hyperplastic glands in E7107-treated prostates showed heterogeneous and generally reduced Ki-67^+^ cells (Fig. 10f).

## Discussion

In this study, by establishing a comprehensive annotated splicing map in the entire spectrum of PCa development and progression, we have made several significant findings (see Supplementary Discussion). First, global splicing dysregulation augments along increasing aggressiveness of PCa and alterations in SAGs minimally change their bulk mRNA expression, indicating aberrant splicing as an independent mechanism (vs. gene expression regulation) driving tumorigenesis^56^. Second, we identify IR as a hallmark of stemness associated with PCa aggressiveness. Interestingly, IR in PCa does not cause NMD-mediated mRNA degradation but instead increases expression of IR-bearing genes involved in stemness and cancer-promoting functions, adding another layer of complexity underlying, and justifies further exploration on the role of IR in, PCa etiology and progression. Third, AR regulates a splicing program, but not IR specifically, distinct from its transcriptional regulation, unveiling an unrecognized role of AR axis in driving PCa progression. Fourth, our data indicates that CNV-mediated deregulation of SRGs is the main mechanism underpinning splicing abnormalities in PCa, unlike hematological cancers that are mainly associated with point mutations in core spliceosome genes^8^. Importantly, most of these SRGs are oncogenic and associated with adverse clinical outcomes. Fifth, the distorted splicing landscape likely contributes to treatment failure and progression in PCa; on the other hand, CRPC displays a preferential dependency on aberrant spliceosome activity and a vulnerability towards spliceosome interference. Sixth, E7107, the spliceosome modulator, effectively inhibits the growth of both Myc-driven murine PCa and preclinical CRPC models in vivo. Last, E7107 promotes differentiation and reprograms PCa cells from an aggressive (AI) to an indolent (AD) state, potentially pointing to a rational strategy of administering splicing inhibitors first for a short term (to avoid toxicity and also to reprogram aggressive PCa cells) followed by Enza treatment for CRPC. Future characterizations of the origins and consequences of aberrant splicing in aggressive PCa could enhance our understanding of disease pathogenesis and aid innovative drug development.

## Methods

### RNA-seq datasets, AS mapping, and bioinformatics

The information on RNA-seq datasets used in this study was detailed in Supplementary Data 2. In general, the mapping of raw RNA-seq reads to human reference genome sequence (GRCh38.p7) and quantification of fragments in each known gene from GENCODE (version 25) were performed using STAR2 v.2.5.2^57^. For gene expression at the bulk RNA level, DESeq2 v.1.18.1^58^ was employed as a statistical procedure to call DEGs for comparisons. For quantification of gene expression at transcript or isoform level, Salmon v0.7.2^22^ was used to quantify isoform expression followed by DEseq2 calling of DEIs in different groups. For example, to reveal the pri-PCa-specific transcriptome, a pair comparison of pri-PCa vs. normal prostate tissues from TCGA cohort was made. Similarly, we compared CRPC (SU2C cohort) vs. TCGA cohort to decode a transcriptome unique to CRPC-Ad. A divergent clonal evolution model has been proposed for the emergence of CRPC-NE from CRPC-Ad^4^. This cohort (named Trento/Cornell/Broad 2016) contained RNA-seq data for both clinical CRPC-NE and CRPC-Ad samples, which were thus used for a pair comparison to generate a CRPC-NE-specific transcriptome. Normalization for differences in sequencing depth of libraries was included when performing abovementioned pair comparisons. Alternatively, to increase the flexible utility of these large clinical PCa datasets without re-running of mapping steps, gene expression was calculated as RPKM (reads per kilo base per million mapped reads) values and the expression variability is quantified for each gene in all cohorts as a *Z*-score relative to the mean expression in TCGA normal prostate samples. RPKM was used in Fig. 2g and Supplementary Fig. 6c.

In this study, we used two AS detecting pipelines, rMATS v4.0^14^ and SUPPA v2.2.0^15^, to assess the differential splicing landscape embedded in RNA-seq data. A widely accepted cutoff of ΔPSI > 0.1 and FDR < 0.1 (for rMATS) or *p* < 0.05 (for SUPPA, which does not provide FDR values) was employed to establish DSEs. We also conducted rMATS-based mapping using an FDR < 0.05, which generated similar results to those when using FDR < 0.1 for rMATS (Supplementary Fig. 1a-c). Of note, these two AS mapping pipelines were different in algorithms, thus preventing a direct cross-comparison of their results. We mainly used them for the purpose of solidifying the overall AS landscape identified in PCa development and progression. Both pipelines utilize the annotated genomic information and rMATS can generate some de novo events, whereas SUPPA by nature detects more significant splicing events (*p* < 0.05). Due to the technicality associated with rMATS (i.e., the numbering of each splicing events is different between comparisons, which precludes direct cross-comparisons of DSEs), we used results of SUPPA for splicing-related GSEA (e.g., Supplementary Fig. 1f-g). Moreover, rMATS required a same length of reads from input libraries, but the reads of TCGA RNA-seq^16^ and CRPC RNA-seq^19^ were about 48 bp and 100–125 bp, respectively. Therefore, to illustrate the AS landscape of CRPC (vs. pri-PCa) by rMATS, we split each paired-end long read in CRPC RNA-seq library into two short paired-end reads with a 48 bp retention of the sequence ends and used them for downstream analysis. SUPPA used fastq files as input and have no preference in read length.

To define manageable lists of DEGs and DEIs, unless otherwise specified, we generally used a stringent statistical threshold of ≥2 FC and FDR of <0.05 plus a baseMean (readcounts) >10 (to remove lowly expressed genes). GO analysis was performed on the genes showing either differential expression (at bulk or isoform level) or aberrant splicing events using Metascape (http://metascape.org). To obtain more reliable results, we mainly used the fewer DEG lists or the top ranked genes (detailed gene number specified in text or figure legends) as input for GO analysis. Terms with *p* < 0.01, minimum count 3, and enrichment factor >1.5 (enrichment factor is the ratio between observed count and the count expected by chance) were considered significant. Significantly enriched terms with similar descriptions and functions were further grouped into distinct biological categories (to better reflect the biology of a context) and top categories were schematically projected on the network of enriched terms. GSEA was carried out by using the curated gene sets (C2) of the Molecular Signature Database (MSigDB) v.4.0^59^. The list of the entire detectable genes with Log2 ratios derived from each comparison was used for pre-rank GSEA, and we followed the standard procedure described by GSEA user guide (http://www.broadinstitute.org/gsea/doc/GSEAUserGuideFrame.html). For splicing GSEA, the list of detected splicing events and ΔPSI values were used. The FDR for GSEA is the estimated probability that a gene set with a given NES (normalized enrichment score) represents a false positive finding and an FDR < 0.25 is considered to be statistically significant.

### Deciphering the splicing code of IR

The differentially spliced introns (i.e., significant IR including Up and Down events) and constitutively spliced introns (*n* = 300, defined as the central events located in a list with a ΔPSI = 0) were used to decipher the splicing code of IR. The sequences of these introns were collected by R package Rsamtools and homemade R scripts (http://bioconductor.org/packages/release/bioc/html/Rsamtools.html) according to their genomic coordinates annotated in the rMATS result tables, followed by calculation of intron length and GC content (% of G and C nucleotides) for each intron. For splice site strength analysis, we used a maximum entropy model (MaxEntScan)^60^ to quantify the 9-base boundaries of 5′ splice sites (3 bases upstream of the exon and 6 bases in the intron) and the 21-base boundaries of 3′ splice sites (1 base downstream of the exon and 20 bases in the intron). The Wilcoxon ranked test was employed to perform statistics.

### RNA-binding proteins motif analysis

Regions spliced alternatively are frequently enriched for *cis*-regulatory elements that could act as splicing enhancers or inhibitors^34^. To identify the potential RBP that may preferentially regulate IR (e.g., in cancer vs. normal tissues), we performed motif analysis of 95 human RBPs with known binding sites. These RBPs, including many well-characterized splicing factors, were collected from the merged information of a RBPmap database^35^ and a recent publication^34^. The sequences of introns with significant IR and introns spliced out constitutively (*n* = 300 with ΔPSI = 0; used as control) were retrieved and used as input for this analysis. We first scanned each sequence for the presence of a motif (appeared at least once) within a group (binding frequency) and the number of motif appearance (how many times the same motif appeared within a sequence) for each RBP. We then calculated an RBP-binding score for each factor based on the multiplication of binding frequency of a given factor across all sequences within a group and the average count of motif appearance within one sequence. The top 20 ranked genes with a binding frequency above 70% were chosen for further analysis and shown in Supplementary Data 8.

### Splicing events associated with AR activities

To decode the AS events regulated by AR signaling in clinical specimens, we first established an AR activity score based on the *Z*-scores calculated from the expression of 20 experimentally validated AR targets (KLK3, KLK2, TMPRSS2, ELL2, CENPN, GNMT, MAF, NNMT, MED28, EAF2, MPHOSPH9, PTGER4, HERC3, ZBTB10, ACSL3, FKBP5, C1orf116, NKX3-1, ABCC4, and PMEPA1)^16^. These 20 genes upregulated in LNCaP cells upon stimulation by synthetic androgen R1881 were used as the gene signature of androgen-induced genes, and an AR activity score was defined by the quantification (*Z*-score) of the composite expression of this 20-gene signature in each clinical specimen^16^. To further associate the AR activities to underlying splicing disruption in patient samples, we fractionated the TCGA and CRPC-Ad cohorts into high and low AR activity groups according to the *Z*-scores (≥7 or ≤ −7), followed by splicing analyses (Fig. 3a, b).

### Calling of differentially expressed SRGs

For Oncomine concept analysis, the list of 274 SRGs was input as a custom concept. Of these, 264 genes were detected by Oncomine (Supplementary Data 1). The comparisons, between tumors (T) to normal (N) and between metastasis to primary tumors, utilized 16 and 13 datasets, respectively, and the actual Oncomine datasets were listed in Supplementary Fig. 12a. By combining the Oncomine (*p* < 0.05) results with DEGs identified in RNA-seq analysis (FC ≥ 1.5 and FDR < 0.1, an acceptable cutoff to capture all possible DEGs) (Supplementary Fig. 12b), we defined a final list of 186 genes as differentially expressed SRGs (Fig. 5).

### Survival analysis

Kaplan–Meier analysis was then performed to associate the gene expression to the patient overall survival (OS) status (Fig. 6a). We surveyed all 186 SRGs in the indicated 7 clinical datasets containing patient survival information (Supplementary Data 5), of which the Sboner dataset was assessed via PrognoScan (http://www.abren.net/PrognoScan/). Notably, Oncomine mainly contained microarray-based data generated earlier, and each dataset normally detected only a subset of 186 genes. Therefore, we downloaded and utilized the available information from each dataset for patient OS analysis.

For each gene, a patient cohort was stratified into two groups with relatively high and low gene expression, and the best cutoff chosen based on the value that yields the lowest *P*-value computationally^61^. Specifically, the algorithm for creating a predictor involves a numeric score and a threshold to classify the subject to a binary (high/low) risk group variable based on the score. It is noteworthy that the Nakagawa dataset only detected two SRGs and was thus excluded from downstream analysis. In addition, the results obtained from the Taylor cohort^47^ was opposite to those from the other five datasets, which consistently displayed similar results. This was mainly due to the issues with clinical information associated with Taylor cohort: few patients died and all deaths were not related to PCa.

For gene signature analysis (Fig. 6c), the numeric score was calculated based on a linear combination of expression values (*Z*-score) of all detected genes in the signature. The Setlur and Glinsky cohorts, which were relatively large and contained more SRGs, were used for signature analysis. The unfavorable and favorable gene signatures were defined as a group of 13 genes (*SRSF1*, *KHDRBS3*, *ESRP1*, *HNRNPH1*, *U2SURP*, *LSM5*, *TIA1*, *CHERP*, *HNRNPR*, *HNRNPH2*, *HNRNPH3*, *KHDRBS1*, and *HNRNPAB*) with each showing consistent unfavorable prognosis in ≥3 datasets and a group of 13 genes (*MFAP1*, *SF3A2*, *GPATCH1*, *XAB2*, *CELF2*, *SF3A1*, *SAP18*, *SRP54*, *PPIL2*, *SF1*, *MATR3*, *ELAVL4*, and *CDK10*) with each showing consistent favorable prognosis in ≥2 datasets, respectively.

### SRG signatures associated with Gleason grade and splicing dysregulation

To further associate clinically prognostic SRG signatures to underlying splicing disruption in patient samples, we focused on the unfavorable gene signature, for it predicted worse outcome and the majority of prognostic SRGs were unfavorable genes. Based on a linear combination of expression values (*Z*-score) of 13 genes in the unfavorable signature, we fractionated the TCGA cohort according to the median value of signature scores into high and low groups. Gleason score and disease recurrence status were compared between these two groups (Fig. 6d, e). Furthermore, top and bottom 20 RNA-seq samples ranked by signature scores were selected for rMATS splicing analysis (Fig. 6f) aiming to establish the link between clinically “worse” tumors and the extent of splicing dysregulation.

### Genomic alterations of SRGs impact gene expression

Conceivably, CNVs may lead to loss or gain of gene expression. We first analyzed the top altered SRGs individually in the TCGA (pri-PCa) and SU2C (mCRPC) cohorts. In TCGA, all the top 10 deleted genes displayed significantly reduced levels of expression, whereas seven out of the top ten amplified genes showed a trend of increase in expression (although not statistically significant) likely due to the small number of samples harboring gene amplifications in heterogenous pri-PCa cohort (Supplementary Fig. 11a). In CRPC, 3 out of top 5 deleted genes and 1 out of top 17 amplified genes exhibited significant changes in bulk mRNA expression (Supplementary Fig. 11b). Nevertheless, the statistically nonsignificant ones did show a trend in that deletion reduced, whereas amplification increased, the gene expression. Again, the statistics was confounded by the small sample size and the heterogeneity of gene expression within the cohort.

### Cell lines and xenograft assays

All cell lines were freshly obtained from American Type Cell Culture and short-term passaged in laboratory. PCa cells were cultured in RPMI-1640 (Life Technologies, Carlsbad, CA) plus 7% FBS, and nontransformed prostate epithelial cells, RWPE1, in PrEGM (Prostate Epithelial Cell Growth Medium; Lonza, Walkersville, MD). Human xenograft prostate tumors, LAPC9 (bone metastasis; AR^+^ and PSA^+^), LAPC4 (lymph node metastasis; AR^+^ and PSA^+^), LNCaP (lymph node metastasis; AR^+^ and PSA^+^), DU145 (brain metastasis; AR^-^ and PSA^−^), and PC3 (bone metastasis; AR^-^ and PSA^−^) were maintained in NOD/SCID or NOD/SCID-IL2Rγ^−/−^ (NSG) male mice. These cell and xenograft lines have been routinely used in our laboratory^25,62^, regularly authenticated by our institutional CCSG Cell Line Characterization Core using short tandem repeat analysis and checked to be free of mycoplasma contamination using the Agilent (Santa Clara, CA) MycoSensor QPCR Assay Kit (catalog number 302107). Recently, we have established a series of transplantable xenograft models that recapitulate the distinct AR protein expression patterns seen in clinical CRPC samples^25^. Briefly, AD (i.e., androgen-sensitive) xenograft tumors, LNCaP, LAPC4, and LAPC9 were routinely maintained in intact 8–10-week-old male immunodeficient NOD/SCID or NSG mice. To establish the AI (i.e., androgen-insensitive or castration-resistant) lines, parental AD tumor cells were purified out, mixed with Matrigel, injected subcutaneously and serially passaged in surgically castrated immunodeficient male mice^25^. The LAPC9 AD and AI xenograft lines were maintained and passaged in NOD/SCID mice, whereas LNCaP AD and AI xenograft lines, and PC3 tumors were passaged in NSG mice. The immunodeficient mice were produced mostly from our own breeding colonies with occasional purchases from the Jackson Laboratories and maintained in standard conditions according to the Institutional Guidelines. Briefly, mice are housed in individually ventilated microisolator caging systems with HEPA filtered air in the room. Cages, bedding, and food are sterilized via autoclaving. Water is processed via reverse osmosis and delivered to cages via an automatic watering system. Autoclaved water bottles containing water acidified with hydrochloric acid to a pH of 2.5–3.5 are also used on some cages. Cages are changed once weekly. All cage changing and experimental manipulations are performed aseptically in biosafety cabinets or laminar flow workstations by personnel using gloved hands and/or forceps dipped in disinfectant. All animal experiments were approved by our Institutional Animal Care and Use Committee in our Laboratory Animal Shared Resource (LASR).

### E7107 drug treatment

For all in-vitro experiments, E7107 was dissolved in dimethyl sulfoxide. For in-vivo administration, E7107 was dissolved in vehicle (10% ethanol and 5% Tween-80 in sterile PBS) and administered via intravenous tail vein injection at 5 mg/kg/day (d). Xenograft-bearing mice were treated for 5 consecutive days (i.e., one cycle) or 10 days (7 days of ‘drug holiday’ between the 2 cycles). Previously, a dose of 4 mg/kg/d was used for treating blood diseases^63^. Given that PCa is a solid tumor, a dose of 5 mg/kg/d was recommended by the company (H3 Biomedicine) based on pilot studies. For drug-efficacy studies in xenograft models, randomization was done when tumor volume reached 150~200 mm^3^ using a calculation of 1/2 (length × width^2^). Animal body weight and tumor growth were measured twice weekly during the experiments. At the end of experiments, tumors were collected and tumor incidence, weight, and gross images were recorded. No blinding was done in the in vivo drug studies or in data analysis. For RNA-seq analysis in the CRPC xenograft models, tumor cells were first implanted in castrated male mice and then subjected to randomization when tumor size reached 150~200 mm^3^, followed by one cycle of E7107 treatment. Mice were killed for tumor collection exactly 4 h after receiving the last injection of five consecutive injections of E7107. For E7107 treatment of FVB Hi-Myc tumors^64^, male mice at 42 weeks (when Ads fully developed) were randomized into two groups (*n* = 16 and 17, respectively). Animals were tail vein injected with one cycle of E7107 (at 5 mg/kg/d) or vehicle (as in the xenograft treatment) for 5 consecutive days followed by 1 week of drug holiday and another cycle of 5-day treatment at 44 weeks (Fig. 10a). All animals were terminated at week 46 and the genitourinary track and prostate were isolated and weighed. Whole-mount prostate was subjected to HE and IHC (MYC, AR, and Ki67) staining and Aperio Scanscope analysis^25^.

### RNA isolation, deep RNA-seq, and real-time qPCR

PCa cell cultures and xenografts were treated with either vehicle or E7107 at indicated doses, then followed by total RNA extraction using a RNeasy mini kit (Qiagen, Hilden, Germany) with inclusion of a DNase I treatment step. For deep RNA-seq, cDNA libraries were constructed using the TruSeq Stranded Total RNA Preparation Kit (catalog number RS-122-2301Illumina, San Diego, CA, http://www.illumina.com), which contained Ribo-Zero^TM^ Gold to deplete rRNA. We amplified our libraries for <15 PCR cycles (as suggested by the manufacturer) to minimize amplification-induced noise. Purified libraries were quantified using a Kapa library quantification kit (KAPA Biosystems, Wilmington, MA). HiSEquation 2500 (Illumina) was used to perform 2 × 76 bp sequencing for cell cultures, and NovaSeq (Illumina) was utilized later for a 2 × 101 bp paired-end sequencing of xenograft samples. Three and five biological replicates per group were included for cell culture and tumor RNA-seq experiments, respectively. On average, we obtained approximately 83.1 M and 100 M pairs of reads for cell cultures and xenografts, respectively, indicating the high depth of sequencing. For real-time quantitative RT-PCR (qPCR), the first-strand cDNA synthesis was achieved by reverse transcription of RNA using random hexamers and SuperScript III Reverse Transcriptase (Invitrogen, Grand Island, NY), followed by qPCR using the iQ™ SYBR^®^ Green supermix (BioRad, Hercules, CA) on a 7900HT Fast Real-Time PCR System (ABI, Applied Biosystems, Foster City, CA). The primers used in this study are listed in Supplementary Data 9. Housekeeping gene *GAPDH* or *β-actin* was used as internal control for gene expression normalization.

### Pharmacological and genetic modulation of AR signaling

AD LNCaP cells were cultured in regular FBS-containing medium. In this study, we employed several complementary approaches to modulate the cellular AR signaling activity (Fig. 3f). First, LNCaP cells were grown for 3 days in phenol-red free medium supplemented with CDSS to mimic an androgen-deprived environment. Second, AR antagonist Enza (10 μM; Selleckchem) was added into the regular culture medium and LNCaP cells were cultured for 3–4 days to mimic the clinical setting of antiandrogen treatment. Third, siRNAs (SMARTpool, L-003400-00-0005; Dharmacon) against scramble (siNC) and AR mRNA (siAR) were employed to knock down endogenous AR. Cells were transfected with 20 nM siRNA oligonucleotides or non-targeting controls using Lipofectamine RNAi MAX (Invitrogen). Knockdown efficiency was determined by qPCR at 48–72 h post transfection. Finally, androgen stimulation assay was performed to acutely boost AR signaling. To this end, LNCaP cells were first primed with CDSS for 2~3 days and then treated with 10 nM DHT for 8 h, a time point when the transcription of PSA, an AR target, reached a plateau.

### siRNAs, MTT, colony formation, and sphere assays

The siRNAs against scramble (siNC, D-001810-10-05; Dharmacon), ESRP1 (SMARTpool, L-020672-01-0005; Dharmacon), KHDRBS3 (SMARTpool, L-012748-01-0005; Dharmacon), SYT7-SE1 (5′-GGGAUAUUGGCAAAGUCAU-3′), and SYT7-SE3 (5′-GCUUCUCAAGGCGUCCUCU-3′) were employed to knock down endogenous SRGs. For individual gene knockdown, cells were transfected with 20 nM siRNA oligonucleotides using Lipofectamine RNAi MAX (Invitrogen). To double knock down ESRP1 and KHDRBS3 together, 10 nM siRNA targeting each was used. Knockdown efficiency was determined by qPCR at 48–72 h post transfection. For cell proliferation (viability) assays, LNCaP (2500 cells/well), PC3 (1000 cells/well), DU145 (1500 cells/well), and RWPE1 (1200 cells/well) were seeded in 96-well plates. MTT was added at a concentration of 0.5 mg/ml for 3 h at 37 °C. The medium was then removed and 0.2 ml/well of acidic isopropyl alcohol (0.04 M HCl in absolute isopropyl alcohol) was added. The absorbance of the converted dye was measured at 570 nm using a Synergy II spectrophotometer (Biotek). For colony formation assays, we generally plated PCa cells at a low density (i.e., 3000–5000 cells/well) in a 6-well dish and let cells grow for 6–7 days before visualization of the culture by crystal violet staining. For inhibitor studies, we usually plated the cells in normal medium on day 1, and then added the inhibitors at varying concentrations on day 2. For sphere-formation assays^25,27^, we generally plated cells at 3000–5000 cells/well in serum-free prostate epithelial basal medium supplemented with 4 μg/mL insulin, B27 (Invitrogen), and 20 ng/mL epidermal growth factor and basic fibroblast growth factor in ultra-low attachment plates. Floating spheres that arose in 1–2 weeks were counted.

### Wound-healing, migration, and invasion assays

For wound-healing assays, PCa cells in six-well culture dishes were allowed to grow to 80~90% confluence, and a sterilized tip was utilized to introduce a scratch “wound” with the same width on the bottom of the dishes. We generally made two scratch wounds per well as technical replicates, and wound closure under multiple microscopic views per well was recorded. Images were captured at 0 and 20–48 h after the wounding depending on cell types. Data shown were representative of three independent repeats. Moreover, cell migration and invasion assays were performed using Boyden chambers (CellBiolabs, San Diego, CA) according to manufacturer’s instructions. Briefly, PCa cells were loaded into the chambers and cultured in media with or without varying concentrations of E7107 for 1–2 days, and results were visualized by PROTOCOL™ Hema 3 staining kit (Fisher Scientific, Pittsburgh, PA). Images of the membranes were captured by Olympus IX71. Data were quantified based on the cell number counting of at least five 10× images.

### Splicing reporter and luciferase assays

A quantitative splicing reporter system^42^, in which a 132-nucleotide chimeric β-globin/immunoglobulin intron was inserted into the firefly luciferase gene, was obtained from Addgene (plasmids #62857 and #62858). Using the same plasmid backbone, the 143-nucleotide intron 3 of *PSA* gene was inserted to test whether AR modulates splicing of AR targets. Cells were plated in 12-well plates and co-transfected, using Lipofectamine 2000, with 250 ng firefly luc reporter plasmid and 20 ng *Renilla* plasmid (phRL-CMV). After transfection, LNCaP cells were grown in different conditions of inhibiting or promoting AR signaling (Supplementary Fig. 7g, i) for 15~24 h, followed by luc activity measurement using Dual-Luciferase® Assay Kit (Cat. # E1980, Promega).

### Histology and immunohistochemistry

For IHC, slides were deparaffinized in xylene and hydrated in gradient alcohols to water. Endogenous peroxidase activity was blocked with 3% H_2_O_2_ for 10 min followed by antigen retrieval in 10 mM Citrate Buffer (pH 6.0). After blocking with Biocare Blocking Reagent (Biocare), slides were incubated with primary antibodies (c-MYC (Ab32072, Abcam, 1 : 1000 dilution), Ki67 (NCL-Ki67p, Leica Biosystems, 1 : 500 dilution), AR (SC-7305, Santa Cruz, 1 : 200 dilution); optimal dilutions were previously determined by antibody titration experiments performed by RPCCC histology core), followed by incubation with secondary antibodies and DAB (BioGenex Laboratories, Inc.) development. IHC images were captured using Olympus IX71 microscope. For quantification of cell numbers, at least 5~6 random high-magnification (×20) images were captured.

### Statistical analysis

Statistical analyses were performed using GraphPad Prism software or using R. In general, Student’s *t*-test, paired or unpaired two-tailed *t*-test, and *χ*^2^-test were used to calculate the statistical significance between comparisons depending on the data type. *P* < 0.05 is considered statistically significant.

### Reporting summary

Further information on research design is available in the Nature Research Reporting Summary linked to this article.

## Supplementary information

Supplementary Information

Description of Additional Supplementary Files

Supplementary Data 1

Supplementary Data 2

Supplementary Data 3

Supplementary Data 4

Supplementary Data 5

Supplementary Data 6

Supplementary Data 7

Supplementary Data 8

Supplementary Data 9

Reporting Summary

## Data Availability

The RNA-seq data have been deposited in GEO database under accession code GSE114052 [https://www.ncbi.nlm.nih.gov/geo/query/acc.cgi?acc=GSE114052], GSE128515 [https://www.ncbi.nlm.nih.gov/geo/query/acc.cgi?acc=GSE128515], and GSE139962 [https://www.ncbi.nlm.nih.gov/geo/query/acc.cgi?acc=GSE139962]. The accession codes for all the publicly available datasets used in this study were provided in the Supplementary Data 2. The source data for Figs. 2a–d, h; 3h; 3a–e; 8a–d, g, k; 9b–e, i; 10b; Supplementary Figs. 3a–d; 5a–c; 7h, i; 3d–g; 14a, b, d; 15a–f; and 16a have been provided as a Source Data file. All other data supporting the findings of this study are available within the article and its Supplementary Information files and from the corresponding authors upon request. A reporting summary for this article is available as a Supplementary Information file.

## Source Data

Source Data
