# Supplementary material for: Intron retention is a hallmark and spliceosome represents a therapeutic vulnerability in aggressive prostate cancer
Source: Nat Commun. 2020 Apr 29;11:2089. doi: 10.1038/s41467-020-15815-7 (PMC7190674; doi:10.1038/s41467-020-15815-7)
Supplement: Supplementary file 1 — Supplementary Information [file 41467_2020_15815_MOESM1_ESM.pdf]

Supplementary Information

Intron retention is a hallmark and spliceosome represents a therapeutic vulnerability in aggressive prostate cancer

Dingxiao Zhang et al.

Summary of Supplementary Materials

Supplementary Note 1
Supplementary References
Supplementary Discussion

Supplementary Figures

Related to Fig. 1
Supplementary Fig. 1. Alterations in AS patterns accompany PCa development, progression and therapy resistance.
Supplementary Fig. 2. Global splicing dysregulation impacts genes associated with cancer pathways.
Supplementary Fig. 3. Splicing alterations generate isoform switches during PCa progression.
Supplementary Fig. 4. Clinically relevant isoforms modulate PCa aggressiveness.

Related to Fig. 2
Supplementary Fig. 5. IR upregulation is a consistent hallmark of PCa development and progression and associated with stemness and aggressiveness.
Supplementary Fig. 6. IR impacts PCa biology.

Related to Fig. 3
Supplementary Fig. 7. AR regulates PCa-associated AS globally, but not IR specifically.

Related to Fig. 4
Supplementary Fig. 8. Prominent SRG deletions in pri-PCa and correlation of SRG amplifications with Gleason grade.
Supplementary Fig. 9. Prominent amplifications of SRGs in CRPC.
Supplementary Fig. 10. Genomic landscape of SRG alterations in PCa.

Related to Fig. 5
Supplementary Fig. 11. Copy number variations (CNVs) in SRGs correlate with gene expression.
Supplementary Fig. 12. Pervasive SRG dysregulation during PCa development and progression.

Related to Fig. 8
Supplementary Fig. 13. Aggressive PCa cells are susceptible to the spliceosome inhibitor E7107.
Supplementary Fig. 14. The E7107 compound inhibits PCa cells via targeting spliceosome.

Related to Fig. 9
Supplementary Fig. 15. E7107 inhibits CRPC in vivo.
Supplementary Fig. 16. E7107 impacts transcriptome of CRPC cells in vivo.

Supplementary Data
Supplementary Data 1. The mutational landscape of 274 SRGs in PCa
Supplementary Data 2. Summary of RNA-seq datasets used in this study for splicing analyses
Supplementary Data 3. Summary of statistically significant differentially spliced events (DSEs)
Supplementary Data 4. Summary of SRG dysregulation
Supplementary Data 5. Summary of the prognostic association of differentially expressed SRGs
Supplementary Data 6. DSEs identified in PCa cells and xenografts treated with E7107
Supplementary Data 7. DEGs identified in PCa cells and xenografts treated with E7107
Supplementary Data 8. RBP motif analysis of IR
Supplementary Data 9. List of primers used in this study

Supplementary Note 1:

Global splicing dysregulation impacts PCa biology

Our systematic splicing mapping analysis revealed an association of increasing DSEs with the degree of PCa malignancy and progression (Fig. 1). We next explored the potential impact of AS dysregulation on PCa biology (Supplementary Fig. 2 and 3). By overlapping the splicing-affected genes (SAGs) and differentially expressed genes (DEGs), we observed only 9~20% of 'overlapped' genes (Supplementary Fig. 2a), suggesting that the majority of AS events minimally changed the bulk gene expression but may functionally tune transcriptomes[1,2]. Gene ontology (GO) analysis (http://metascape.org) indicated that the SAGs were enriched for many cancer-associated functional categories with both convergence (e.g., splicing, cell cycle and proliferation, cytoskeleton) and specificity identified at each PCa stage (Supplementary Fig. 2b-d). For instance, GO terms linked to 'muscle and ion transport', 'lipid metabolism', and 'cell polarity' were pri-PCa specific (Supplementary Fig. 2b) whereas GO terms 'DNA damage', 'immunity', and 'nuclear pore' were enriched in CRPC (Supplementary Fig. 2c), consistent with recent reports[3]. Interestingly, and as expected, GO terms 'SCs and development' and 'neuron and cell projection' were greatly enriched in CRPC-NE (Supplementary Fig. 2d), in line with its stem-like and neural-like properties.

We further evaluated the potential functional consequences of AS dysregulation on PCa transcriptome by identifying transcript-level expression profiles using an isoform-specific alignment algorithm[4]. As shown in Supplementary Fig. 3a, PCa at different stages exhibited distinct splice isoform signatures. For instance, the widely studied ARv7 was only slightly upregulated in pri-PCa but, together with several other AR variants, was dramatically overexpressed in CRPC-Ad (but not in CRPC-NE due to loss of AR expression in NE tumors) (Supplementary Fig. 3b). CD44, a cancer stem cell (CSC) marker, plays versatile roles in metastasis with CD44-standard (CD44s; CD44-201) suppressing and CD44 variants (CD44v) promoting cancer cell colonization[5]. Consistently, we observed a shift from no change in pri-PCa (vs. N) to a specific dysregulation of CD44 isoforms in mCRPC, with CD44s being downregulated in both CRPC-Ad and CRPC-NE and CD44v dramatically upregulated in CRPC-Ad (Supplementary Fig. 3c). The splicing program driving CRPC-NE emergence is scantly explored. Recently, an SE event leading to unique upregulation of a MEAF6 isoform containing exon 6 (i.e., MEAF6-204), but not the bulk mRNA, was reported in CRPC-NE[6]. We observed similar results (Supplementary Fig. 3d), thus validating our approaches.

In addition to these well-annotated genes in impacting PCa biology and modulating PCa phenotypes, many other differentially expressed isoforms (DEIs) may also play important roles in regulating stemness and tumor progression, although the majority of them lacked functional characterization in cancers. In pri-PCa (vs. N), a protein-coding isoform of ADAM2 (ENST00000265708.8) was significantly upregulated (FC=16.86). Despite a lack of direct study of ADAM2 in PCa, information on ADAM family generally

supported a positive role of ADAM proteins in promoting cell adhesion, migration and invasion[7]. We also observed 4 distinct protein-coding isoforms of SERPINA5 (ENST00000556775.5, ENST00000554866.5, ENST00000329597.11, ENST00000554276.1) markedly down-regulated in pri-PCa. Loss of SERPINA5 expression has been reported in advanced-stage serous ovarian tumor[8]. In CRPC (vs. pri-PCa), the level of a transcript (ENST00000430799.7) encoding the long isoform of ARID1A protein (ARID1A-L) was dramatically increased (FC>23602). ARID1A belongs to the ATP-dependent chromatin remodeling BAF complex and the ARID1A-L is critical for Ewing sarcoma (ES) maintenance and for oncogenic transformation[9]. Of interest, in CRPC-NE (vs. CRPC-Ad), multiple DLK1 splice variants (ENST00000341267.8, ENST00000331224.10, ENST00000556051.1; Supplementary Fig. 3e) were found significantly upregulated. These isoforms have been reported to be expressed at early mouse embryogenesis[10] and DLK1$^+$ cells in human prostate have been shown to localize, preferentially, to the basal cell layer close to the urethra and exhibit primitive SC properties[11], suggesting a functional link of DLK isoforms with stemness. Experimentally, by using SYT7 as an example (Supplementary Fig. 4), we demonstrated that knocking down clinically relevant isoforms of SYT7 in both PC3 and DU145 cells modulated PCa cell biology. It should be noted that many of studies in this project focused on PC3 cells because this model resembles CRPC both transcriptionally[12] and genomically (Supplementary Fig. 13a; below).

Collectively, these analyses indicate that splicing abnormalities impact many genes enriched in cancer-related pathways, and isoform switching of key genes may contribute to PCa aggressiveness and progression.

Positive correlation of IR with stemness

The aggressive PCa subtypes (Fig. 1g-1j), prostatic basal/stem cells (Fig. 1k) and several other SC systems (Supplementary Fig. 5a-c) all displayed a higher level of IR. To further reveal the potential role of IR in stemness, we focused on IR-affected genes in CRPC-Ad (vs. pri-PCa), as more IR events were identified in this comparison. We excluded the CRPC-NE from comparison because both CRPC-Ad and CRPC-NE are CRPC subtypes. We have shown that basal/stem-specific AS profile is enriched in CRPC-Ad (Supplementary Fig. 1f). Overlapping of CRPC- and basal/stem-IR genes revealed 53 shared genes (Supplementary Fig. 5d), among which HMGN2 is highly expressed in ESCs and plays a key role in the establishment and maintenance of cell identity[13]. Overexpression of NRBP2 has been shown to increase the stemness of hepatocellular carcinoma cells via AKT signaling[14]. Comparison of IR genes in CRPC-Ad vs. hESC (vs. differentiated Fibroblasts) demonstrated that 32.8% of CRPC-IR genes also displayed IR events in hESC, directly suggesting a functional link of IR with stemness (Supplementary Fig. 5e). CENPT, encoding a centromere protein, is one of these IR-affected genes, and a defect in CENPT causes a syndrome of severe growth failure[15]. KLF4, recently reported to be overexpressed in murine prostate SCs, regulates their homeostasis and also controls the self-renewal of tumor-initiating cells[16]. Similarly,

overlapping of IR genes in CRPC-Ad and $CD4^+$ T cells indicated that nearly 50% of the CRPC-IR genes also harbored IR in stem-like resting $CD4^+$ T cells (Supplementary Fig. 5f). Among them was ING4, which was previously proposed as a tumor suppressor in PCa with ability to inhibit stemness and promote differentiation of basal epithelial cells into luminal cells[17]. IR-affected genes in different PCa stages show limited overlapping (Supplementary Fig. 5g), suggesting context specificity. This is expected as the cancer transcriptome changes dramatically along with progression. Among the shared 8 genes (Supplementary Fig. 5g), CCDC28B is associated with Bardet-Biedl syndrome (BBS) and participates in the development of cilia. As a developmentally regulated gene, DRG2 is known to regulate cell growth and differentiation of stem cells (information extracted from NCBI-Gene). An IR event of AP1G2 was also found in human $CD34^+$ progenitor cells isolated from the myelodysplastic syndromes (MDS) patients[18]. NME4 is recently reported to behave as an oncogene promoting non- small cell lung cancer stemness and progression[19]. These discussions suggest that IR represents a conserved phenomenon associated with development and stemness with many IR-affected genes shared by different stem-like and cancer cell entities.

Potential functional role of IR

The average ΔPSI values for upregulated IR in different PCa stages were moderately low (ranging from 0.14 to 0.18) (Supplementary Fig. 6g), consistent with recent reports[20,21]. It is noteworthy, though, that these IR events were indeed expressed in clinical samples as visualized by RNA-seq reads mapping to intron regions (Supplementary Fig. 6g, bottom). To further establish the potential functional relevance of IR in PCa biology, we analyzed the lengths of the retained introns and observed that ~30% of retained introns were multiples of 3 in length cross all conditions (Supplementary Fig. 6h, upper), suggesting that these introns might have the potential to encode peptides without disrupting the coding capability of the parent genes. We also analyzed the features of the intron sequences by using ORF-Predictor[22] to predict whether the intron harbors a peptide-coding region (based on the presence of a start codon 'ATG' in a DNA sequence minimal of >75nt in length). The results indicated that about 50~80% of IRs could potentially encode micro-peptides (Supplementary Fig. 6h, lower). IR has been reported to serve as a source of neoepitopes in melanoma[23].

Supplementary References

1 Sebestyen, E. *et al.* Large-scale analysis of genome and transcriptome alterations in multiple tumors unveils novel cancer-relevant splicing networks. *Genome research* **26**, 732-744, doi:10.1101/gr.199935.115 (2016).

2 Sveen, A., Kilpinen, S., Ruusulehto, A., Lothe, R. A. & Skotheim, R. I. Aberrant RNA splicing in cancer; expression changes and driver mutations of splicing factor genes. *Oncogene* **35**, 2413-2427, doi:10.1038/onc.2015.318 (2016).

3 Rodriguez-Bravo, V. *et al.* Nuclear Pores Promote Lethal Prostate Cancer by Increasing POM121-Driven E2F1, MYC, and AR Nuclear Import. *Cell* **174**, 1200-1215 e1220, doi:10.1016/j.cell.2018.07.015 (2018).

4 Patro, R., Duggal, G., Love, M. I., Irizarry, R. A. & Kingsford, C. Salmon provides fast and bias-aware quantification of transcript expression. *Nature methods* **14**, 417-419, doi:10.1038/nmeth.4197 (2017).

5 Yae, T. *et al.* Alternative splicing of CD44 mRNA by ESRP1 enhances lung colonization of metastatic cancer cell. *Nature communications* **3**, 883, doi:10.1038/ncomms1892 (2012).

6 Lee, A. R. *et al.* Alternative RNA splicing of the MEAF6 gene facilitates neuroendocrine prostate cancer progression. *Oncotarget* **8**, 27966-27975, doi:10.18632/oncotarget.15854 (2017).

7 Mochizuki, S. & Okada, Y. ADAMs in cancer cell proliferation and progression. *Cancer Sci* **98**, 621-628, doi:10.1111/j.1349-7006.2007.00434.x (2007).

8 Bijsmans, I. T. *et al.* Loss of SerpinA5 protein expression is associated with advanced-stage serous ovarian tumors. *Modern pathology : an official journal of the United States and Canadian Academy of Pathology, Inc* **24**, 463-470, doi:10.1038/modpathol.2010.214 (2011).

9 Selvanathan, S. P. *et al.* EWS-FLI1 modulated alternative splicing of ARID1A reveals novel oncogenic function through the BAF complex. *Nucleic Acids Res* **47**, 9619-9636, doi:10.1093/nar/gkz699 (2019).

10 Miller, A. J. & Cole, S. E. Multiple Dlk1 splice variants are expressed during early mouse embryogenesis. *Int J Dev Biol* **58**, 65-70, doi:10.1387/ijdb.130316sc (2014).

11 Moad, M. *et al.* Multipotent Basal Stem Cells, Maintained in Localized Proximal Niches, Support Directed Long-Ranging Epithelial Flows in Human Prostates. *Cell reports* **20**, 1609-1622, doi:10.1016/j.celrep.2017.07.061 (2017).

12 Zhang, D. *et al.* Stem cell and neurogenic gene-expression profiles link prostate basal cells to aggressive prostate cancer. *Nature communications* **7**, 10798, doi:10.1038/ncomms10798 (2016).

13 He, B. *et al.* Binding of HMGN proteins to cell specific enhancers stabilizes cell identity. *Nature communications* **9**, 5240, doi:10.1038/s41467-018-07687-9 (2018).

14 Zhang, L. *et al.* NRBP2 Overexpression Increases the Chemosensitivity of Hepatocellular Carcinoma Cells via Akt Signaling. *Cancer research* **76**, 7059-7071, doi:10.1158/0008-5472.CAN-16-0937 (2016).

15 Hung, C. Y. *et al.* A defect in the inner kinetochore protein CENPT causes a new syndrome of severe growth failure. *PloS one* **12**, e0189324, doi:10.1371/journal.pone.0189324 (2017).

16 Xiong, X. *et al.* KLF4, A Gene Regulating Prostate Stem Cell Homeostasis, Is a Barrier to Malignant Progression and Predictor of Good Prognosis in Prostate Cancer. *Cell reports* **25**, 3006-3020 e3007, doi:10.1016/j.celrep.2018.11.065 (2018).

17 Berger, P. L. *et al.* Transient induction of ING4 by Myc drives prostate epithelial cell differentiation and its disruption drives prostate tumorigenesis. *Cancer research* **74**, 3357-3368, doi:10.1158/0008-5472.CAN-13-3076 (2014).

18 Pellagatti, A. *et al.* Impact of spliceosome mutations on RNA splicing in myelodysplasia: dysregulated genes/pathways and clinical associations. *Blood* **132**, 1225-1240, doi:10.1182/blood-2018-04-843771 (2018).

19 Wang, W. *et al.* NME4 may enhance nonsmall cell lung cancer progression by overcoming cell cycle arrest and promoting cellular proliferation. *Mol Med Rep* **20**, 1629-1636, doi:10.3892/mmr.2019.10413 (2019).

20 Dvinge, H. & Bradley, R. K. Widespread intron retention diversifies most cancer transcriptomes. *Genome Med* **7**, 45, doi:10.1186/s13073-015-0168-9 (2015).

21 Kahles, A. *et al.* Comprehensive Analysis of Alternative Splicing Across Tumors from 8,705 Patients. *Cancer cell* **34**, 211-224 e216, doi:10.1016/j.ccell.2018.07.001 (2018).

22 Min, X. J., Butler, G., Storms, R. & Tsang, A. OrfPredictor: predicting protein-coding regions in EST-derived sequences. *Nucleic Acids Res* **33**, W677-W680, doi:10.1093/nar/gki394 (2005).
23 Smart, A. C. *et al.* Intron retention is a source of neoepitopes in cancer. *Nat Biotechnol* **36**, 1056-1058, doi:10.1038/nbt.4239 (2018).
24 Paschalis, A. *et al.* Alternative splicing in prostate cancer. *Nat Rev Clin Oncol* **15**, 663-675, doi:10.1038/s41571-018-0085-0 (2018).
25 Fei, T. *et al.* Genome-wide CRISPR screen identifies HNRNPL as a prostate cancer dependency regulating RNA splicing. *Proceedings of the National Academy of Sciences of the United States of America* **114**, E5207-E5215, doi:10.1073/pnas.1617467114 (2017).
26 Takayama, K. I. *et al.* Dysregulation of spliceosome gene expression in advanced prostate cancer by RNA-binding protein PSF. *Proceedings of the National Academy of Sciences of the United States of America* **114**, 10461-10466, doi:10.1073/pnas.1706076114 (2017).
27 Wang, B. D. *et al.* Alternative splicing promotes tumour aggressiveness and drug resistance in African American prostate cancer. *Nature communications* **8**, 15921, doi:10.1038/ncomms15921 (2017).
28 Ryan, M. *et al.* TCGASpliceSeq a compendium of alternative mRNA splicing in cancer. *Nucleic Acids Res* **44**, D1018-1022, doi:10.1093/nar/gkv1288 (2016).
29 Jung, H. *et al.* Intron retention is a widespread mechanism of tumor-suppressor inactivation. *Nature genetics* **47**, 1242-1248, doi:10.1038/ng.3414 (2015).
30 Boutz, P. L., Bhutkar, A. & Sharp, P. A. Detained introns are a novel, widespread class of post-transcriptionally spliced introns. *Genes & development* **29**, 63-80, doi:10.1101/gad.247361.114 (2015).
31 Simon, J. M. *et al.* Variation in chromatin accessibility in human kidney cancer links H3K36 methyltransferase loss with widespread RNA processing defects. *Genome research* **24**, 241-250, doi:10.1101/gr.158253.113 (2014).
32 Lee, S. C. & Abdel-Wahab, O. Therapeutic targeting of splicing in cancer. *Nat Med* **22**, 976-986, doi:10.1038/nm.4165 (2016).
33 Climente-Gonzalez, H., Porta-Pardo, E., Godzik, A. & Eyras, E. The Functional Impact of Alternative Splicing in Cancer. *Cell reports* **20**, 2215-2226, doi:10.1016/j.celrep.2017.08.012 (2017).
34 Kumar, A. *et al.* Exome sequencing identifies a spectrum of mutation frequencies in advanced and lethal prostate cancers. *Proceedings of the National Academy of Sciences of the United States of America* **108**, 17087-17092, doi:10.1073/pnas.1108745108 (2011).
35 Eskens, F. A. *et al.* Phase I pharmacokinetic and pharmacodynamic study of the first-in-class spliceosome inhibitor E7107 in patients with advanced solid tumors. *Clin Cancer Res* **19**, 6296-6304, doi:10.1158/1078-0432.CCR-13-0485 (2013).

Supplementary Discussion:

Studies of AR splice variants, ARv7 in particular, have implicated splicing dysregulation in PCa resistance to ADT/Enza[24]. Recently, splicing factor HNRNPL was identified as a dependency for LNCaP cells[25] and SFPQ (i.e., PSF) was reported to promote AR splicing and CRPC cell survival[26]. An examination of race-specific AS changes in PCa in African American (AA) vs. European American (EA) men discovered an AA-enriched PIK3CD isoform that promotes tumor aggressiveness and drug resistance[27]. Globally, splicing dysregulation in pri-PCa (vs. N) has been observed[21,28]. While these studies implicate splicing dysregulation in PCa pathogenesis, the global AS landscape unraveling the dynamic evolution of PCa has not been reported and the impact of aberrant AS alterations on PCa progression, therapy resistance, and patient outcome remains undefined.

Here, we provide a comprehensive annotated splicing map in PCa using clinical and experimental RNA-seq data covering the entire spectrum of PCa development and progression. Strikingly, we have observed increasing splicing dysregulation (i.e., DSEs) in association with PCa progression, therapy resistance, and lineage plasticity. Aberrantly spliced genes specific to different PCa stages are both convergently and uniquely enriched in diverse GO terms and pathways linked to oncogenic processes, which establishes aberrant splicing as a distinct mechanism (vs. gene expression regulation) driving PCa progression and therapy resistance. We further identify IR as a hallmark of stemness and aggressiveness during PCa progression. Recently, widespread IR, associated with somatic single-nucleotide variations in six cancer types (excluding PCa), has been observed to be more enriched in TS genes leading to their loss of expression[29]. Surprisingly, we did not observe a similar trend in PCa. Rather, our data reveals that IR generally enhances gene expression and thus likely functions in PCa biology, suggesting different roles of IR in distinct cancer types. Particularly, we find that IR in PCa impacts genes involved in stemness and cancer-promoting functions (Supplementary Fig. 5 and 6), and that AR regulates a splicing program, but not IR specifically, distinct from its transcriptional regulation, suggesting IR as a PCa-regulating mechanism independent of AR axis. In fact, we have observed a generally negative association of AR activity with IR level in multiple clinical datasets (Fig. 2 and 3). Together, our results establish IR as a common mechanism of cellular stemness, as supported by studies in mouse ESCs[30]. The IR prevalent in PCa is not associated strongly with *cis*-genomic features but seems to be regulated by *trans*-regulatory mechanisms involving the combinatorial effects of multiple SRGs. In support, candidate RBPs modulate not only the IR but also other splicing types as well (Fig. 2f). Alternatively, besides altered spliceosome activity, IR might also be modulated by other molecular alterations. For example, loss-of-function mutations in SETD2 (a H3K36 methyltransferase) and subsequent loss of H3K36 trimethylation at target exons are associated with increased IR in renal cancers[31]. Our work expands the view of molecular complexity underlying, and justifies further exploration on the role of IR in, PCa etiology and progression.

There are many ways by which RNA splicing can be dysregulated in cancer. Previously, recurrent point mutations in core spliceosome genes (e.g., SF3B1, U2AF1, SRSF2, ZRSR2) have been reported to drive splicing dysregulation in hematological cancers[32]. Our genomic analyses of SRGs reveal CNVs as the main driver of AS alterations in PCa, which alter the expression of affected SRGs and illustrate cancer type-specific differences in mechanisms of splicing dysregulation. Remarkably, the majority of the top altered SRGs are located in regions containing either TS genes or oncogenes (Supplementary Fig. 10a), and have not been highlighted in previous large-scale DNA sequencing studies. This raises an interesting question of whether these alterations are just passenger mutations or causally contribute to PCa pathogenesis. Our experimental data supports the latter, as knocking down two amplified, clinically relevant SRGs twists splicing landscape and inhibits PCa cell behavior (Fig. 7). The involvement of these genes in other types of cancer has been reported[2,5,24]. Splicing dysregulation has been recently proposed as a 'driver' of transformation independently of oncogenic processes[33]. Therefore, these mutated SRGs may bear some of the 'driver' properties, and it would be interesting, in future studies, to dissect whether deletion or amplification of CNV-associated SRGs with or without collateral alterations in RB1 or MYC loci, or vice versa, could change cancer phenotypes.

Another potential mechanism that may cause splicing abnormality is the mutations in splice sites[32]. However, mutations in splice sites constitute the minority of all somatic mutations (as low as ~0.6%) in PCa[34]; consequently, we reason that deregulation of SRGs is the main mechanism underpinning splicing abnormalities. In support, the majority of SRGs are mis-expressed in various stages of PCa, consistent with studies showing that altered expression of SRGs, even in the absence of mutations, promotes oncogenesis[2]. Of clinical significance, our study has identified many SRGs that can be linked, individually or in combination, to clinical features of advanced PCa, indicating a biomarker value. Almost all of these identified prognostic SRGs and DSEs have not previously been implicated in PCa, warranting further investigation. Notably, the unfavorable SRG signature that we developed herein correlates with poor survival, predicts PCa progression, associates with twisted splicing landscape, and establishes splicing misregulation as a promoter of PCa aggressiveness.

Multiple lines of evidence reveal a preferential dependency of aggressive PCa and CRPC on aberrant spliceosome activity. First, the number of DSEs increases exponentially along the spectrum of cancer progression, linking the severity of splicing dysregulation to PCa aggressiveness. Second, amplifications of SRGs are predominantly observed, and CNVs of SRGs mainly impact global splicing, in CRPC. Third, more SRGs are dysregulated in CRPC, highlighting a potentially critical role of SRG misexpression in driving CRPC evolution. Fourth, the majority of altered SRGs are predictive of worse patient outcome and the unfavorable SRG signature associates with high tumor grade and more prominent disruption in the splicing landscape. Fifth, chemical castration and Enza, both of which target AR signaling, reshape the splicing landscape in PCa cells, and the distorted splicing landscape likely contributes to subsequent treatment failure and disease progression (Fig. 3), as documented in other

cancer types[32]. Finally, of clinical significance, E7107, the spliceosome modulator, effectively inhibits the growth of both Myc-driven autochthonous murine PCa as well as preclinical CRPC models *in vivo* (Fig. 8-10). A phase-I study of E7107 in patients with advanced solid tumors (not including PCa) was terminated due to side effects[35] and we also observed certain toxicities of E7107 in animals (this study), suggesting the need to define intricate treatment window and doses for E7107. Our results may point to a rational strategy of administering E7107, or other splicing inhibitors, as we show that E7107 promotes PCa cell differentiation and reprograms PCa cells from an androgen-insensitive to an androgen-sensitive state. We thus envision a potential treatment regimen in which CRPC is first subject to a short-term splicing inhibition (to avoid toxicity and also to reprogram aggressive PCa cells) followed by Enza treatment. Ongoing studies are exploring the value of this sequential treatment protocol. Overall, our findings suggest that there may be a therapeutic window for spliceosome modulators in the treatment of CRPC. Future studies that aim to determine the origins and consequences of aberrant splicing in aggressive PCa could enhance our understanding of disease pathogenesis and aid innovative drug development.

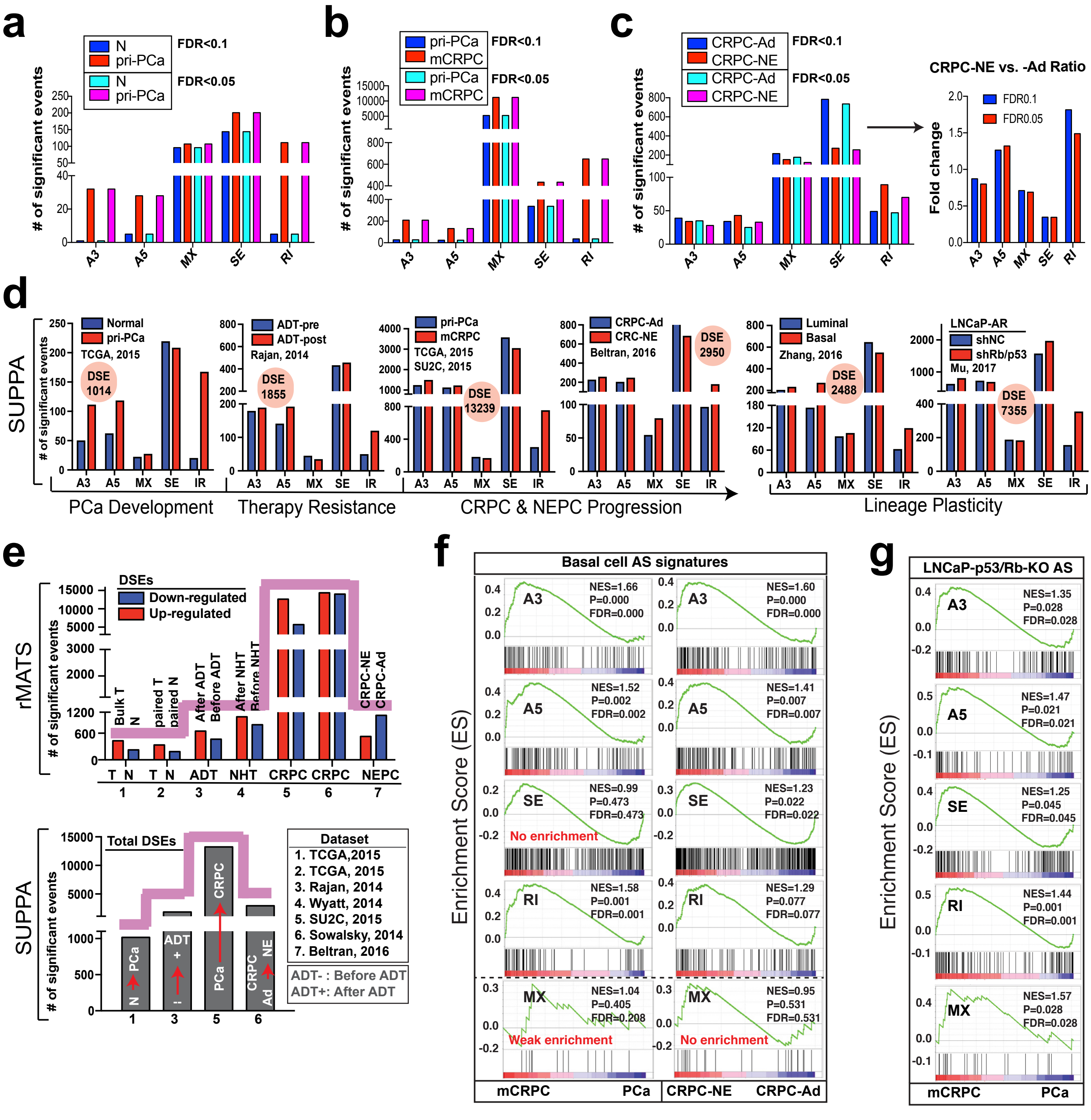

Supplementary Figure 1

Supplementary Figure 1. Alterations in AS patterns accompany PCa development, progression and therapy resistance.

(**a-c**) rMATS-based AS mapping using ΔPSI>0.1 and FDR< 0.1 vs. FDR<0.05 generates virtually identical AS patterns and changes across disease progression. (**a**) TCGA pri-PCa (vs. N) dataset. (**b**) SU2C-mCRPC (vs. pri-PCa) dataset. (**c**) CRPC-NE (vs. CRPC-Ad) dataset. The number (**a-c**) and ratio (**c**, right) of differentially spliced events (DSEs) identified in the indicated contexts were plotted. See also rMATS results using ΔPSI>0.1 and FDR< 0.1 in Fig. 1c-l.

(**d**) The AS landscape during PCa development and progression. Shown is information for pairwise comparisons of RNA-seq datasets and the number of DSEs identified by SUPPA.

(**e**) Comparison of the numbers of identified DSEs in the indicated datasets. Upper: the number of significantly up- and down- regulated DSEs detected by rMATS. Lower: the total number of DSEs detected by SUPPA.

(**f** and **g**) GSEA showing enrichment of splicing signatures specific to basal cells (**f**) and LNCaP cells depleted of p53 and Rb1 (**g**) in aggressive stages of PCa indicated in specified comparisons. The FDR for GSEA is the estimated probability that a gene set with a given NES (normalized enrichment score) represents a false positive finding, and an FDR < 0.25 is considered to be statistically significant.

A3, alternative 3'-splice sites; A5, alternative 5'-splice sites; MX, mutually exclusive exons; SE, exon skipping; IR, intron retention.

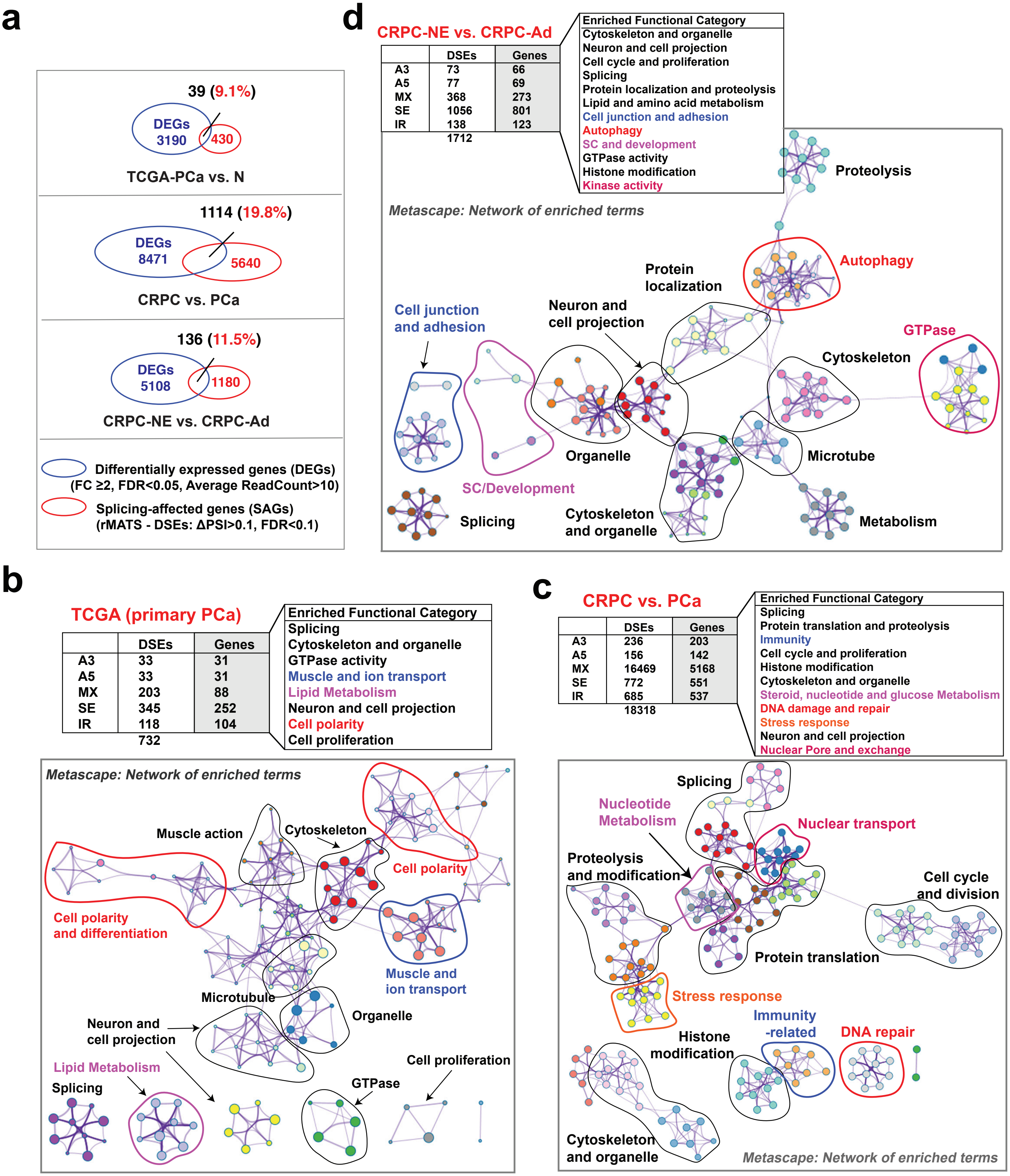

CRPC-NE vs. CRPC-Ad

	DSEs	Genes
A3	73	66
A5	77	69
MX	368	273
SE	1056	801
IR	138	123
	1712	

TCGA (primary PCa)

	DSEs	Genes
A3	33	31
A5	33	31
MX	203	88
SE	345	252
IR	118	104
	732	

CRPC vs. PCa

	DSEs	Genes
A3	236	203
A5	156	142
MX	16469	5168
SE	772	551
IR	685	537
	18318	

Supplementary Figure 2

Supplementary Figure 2. Global splicing dysregulation impacts genes associated with cancer pathways.

(**a**) Overlap between differentially expressed genes (DEGs) and genes showing significant SAGs (i.e., splicing-affected genes) identified in the indicated pair comparisons. The number in parentheses denotes the percentage of overlapped genes proportional to all aberrantly spliced genes. Note increased overlap in DSEs and DEGs in CRPC vs. pri-PCa (19.8%) compared to pri-PCa vs. N (9.1%).

(**b-d**) GO (Gene Ontogeny) analysis of aberrantly spliced genes (i.e., splicing-affected genes or SAGs) reveals an impact of global splicing alterations on PCa biology by regulating genes associated with cancer pathways. Genes showing DSEs in pri-PCa (vs. normal tissues) (**b**), CRPC (vs. pri-PCa) (**c**), and CRPC-NE (vs. CRPC-Ad) (**d**) were used as input for Metascape analysis. Due to a large number of MX events detected in CRPC and a limit of maximal 3,000 genes that could be used for Metascape, we combined genes with A3, A5, SE and IR first and then used genes with top ranked MX to make up a list of 3,000 genes. Terms with p-value <0.01, minimum count 3, and enrichment factor >1.5 (the ratio between observed count and the count expected by chance) were considered significant. The terms with similar descriptions and functions were grouped into different functional categories, and top categories were schematically projected on the network of enriched terms. Categories specific to each PCa stage were colored. Results indicate that cancer-stage-specific SAGs are enriched in GO terms that are functionally connected into well-linked interaction networks, suggesting that splicing dysregulation has a broad impact on PCa biology.

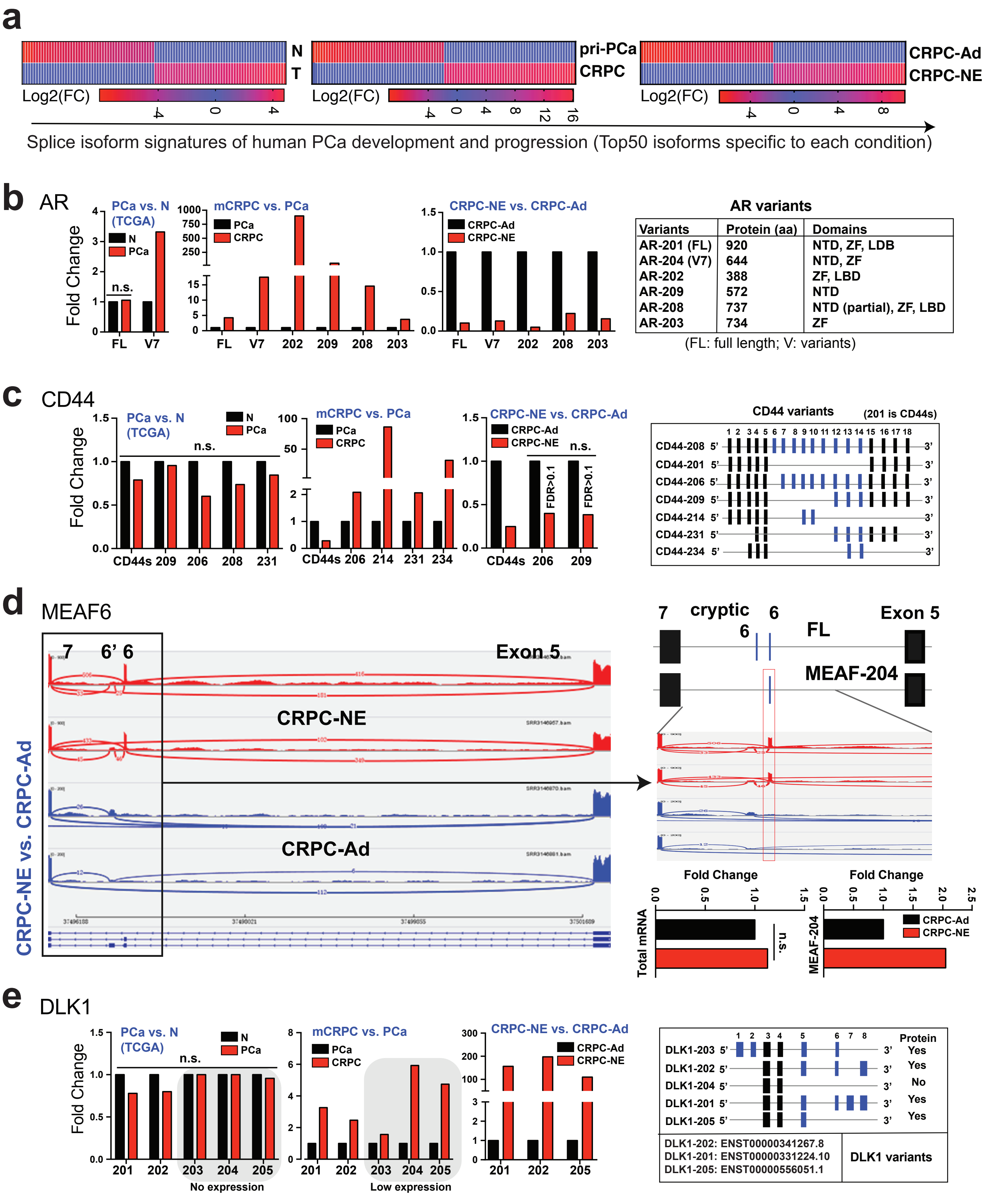

Supplementary Figure 3

Supplementary Figure 3. Splicing alterations generate isoform switches during PCa progression.

(**a**) PCa at different stages exhibit distinct splice isoform signatures. Shown are splicing isoform heatmaps of PCa progression generated using the top 50 differentially expressed isoforms identified in the indicated comparisons.

(**b**) Significant changes in AR splicing accompanies PCa progression and therapy resistance. Shown are the expression patterns and levels of human AR full-length (FL) and protein-coding variants (V, and 202, 203 etc) during different stages of PCa. Note that there was only a slight increase in AR-V7 in pri-PCa (vs. N) but significantly elevated levels of AR-V7 and most other AR variants were observed in mCRPC (vs. pri-PCa). Also note that, as expected, both AR-FL and AR variants were significantly reduced in CRPC-NE (vs. CRPC-Ad). NTD: N-terminal domain; LBD, ligand-binding domain; ZF, zinc finger domain.

(**c**) Significant changes in CD44 splicing accompanies PCa progression. Shown are the expression patterns and levels of human CD44 and its variants (CD44-201 is the CD44s and others are CD44 splice variants) during PCa progression. Note that there was no change in CD44 splice variants in pri-PCa (vs. N) but significantly elevated levels of CD44 splicing variants were observed in mCRPC (vs. pri-PCa).

(**d**) An example of isoform switch during progression from CRPC-Ad to CRPC-NE. Shown is the isoform switch of MEAF6 gene in CRPC-NE vs. CRPC-Ad samples as a result of exon 6 retention, without changing the total gene expression level. There was a 2-fold increase in the MEAF6 isoform (i.e., MEAF-204) in CRPC-NE (right bottom).

(**e**) Significant increase in DLK1 splice variants in CRPC-NE. Shown are the expression patterns and levels of DLK1 (i.e., DLK1-203) and its splicing isoforms in the three comparisons. Note significantly increased DLK1 isoforms in CRPC-NE (vs. CRPC-Ad).

P values were calculated using two-tailed unpaired Student's *t*-test (n.s, not significant).

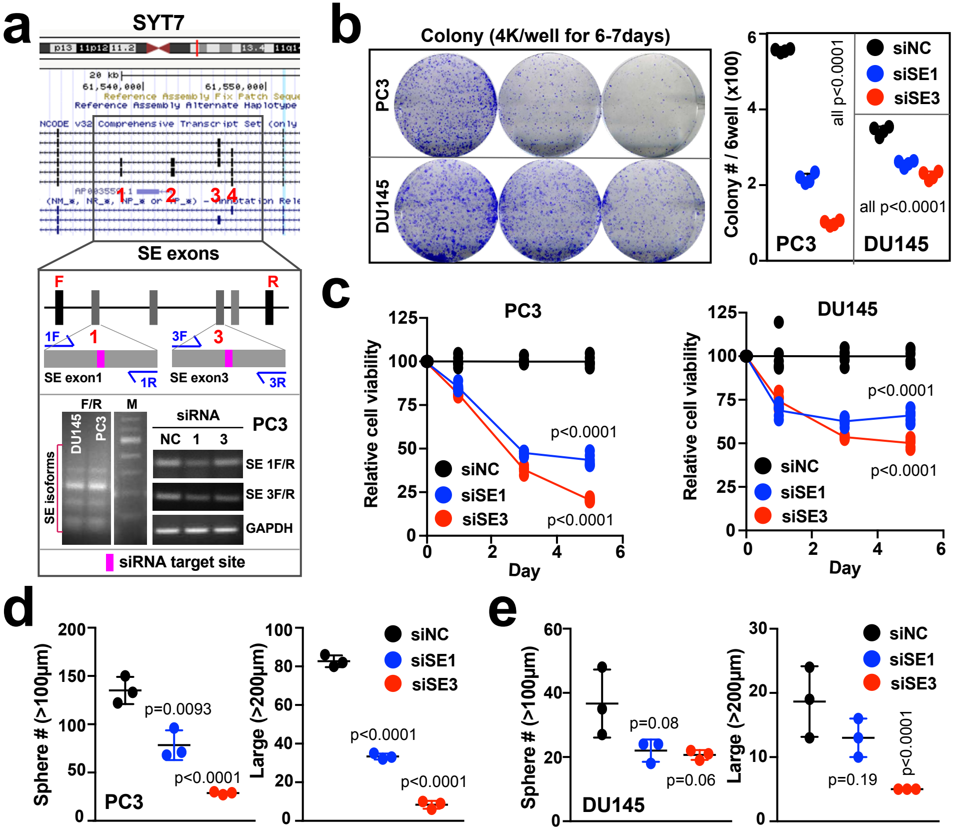

Supplementary Figure 4

Supplementary Figure 4. Clinically relevant isoforms modulate PCa aggressiveness.

(**a**) Schematic illustrating the SE events in SYT7 transcripts detected in clinical CRPC-Ad (vs. pri-PCa) samples (upper) and experimental design (bottom) employing siRNAs to specifically deplete alternative exon-retaining isoforms for biological interrogation. The 4 exons impacted by SE (SE exons) are indicated as 1-4 (boxed). Both DU145 and PC3 express multiple isoforms of SYT7 and siRNAs specifically downregulated the isoforms harboring the indicated exons revealed by semi-quantitative RT-PCR. The RT-PCR experiments were repeated two times independently with similar results. F and R were primers located within the flanking exons of the 4 alternative exons to detect isoforms with variable exons. 1F/1R and 3F/3R were primer pairs used to monitor the expression of indicated exon 1 and 3, respectively. For the gel images in PC3 cells treated with siRNAs (lower right), the sizes of 1F/R, 3F/R and GAPDH were about 112, 192 and 148 bp, respectively.

(**b**) Knocking down SE-bearing SYT7 isoforms inhibits clonal growth in PC3 and DU145 cells. Shown are representative images of two repeat experiments (left) and quantification data (right). Error bars represent the mean ± S.D (n=3).

(**c**-**e**) Knocking down SE-bearing SYT7 isoforms inhibits proliferation (**c**) and sphere formation (**d**, **e**) in PC3 and DU145 cells. Cell proliferation (viability) was determined by MTT assays (**c**). Data represent the mean ± S.D from a representative experiment with 4 technical repeats, and the experiment was replicated 2 times with similar results. Spheres were enumerated 9 days after plating (3500 cells/well) (**d** and **e**) (mean ± S.D; n=3).

siES1 or SE3 denotes knockdown by siRNAs against alternative exon 1 or 3.

All P values were calculated using two-tailed unpaired Student's *t*-test.

Source data are provided as a Source Data File.

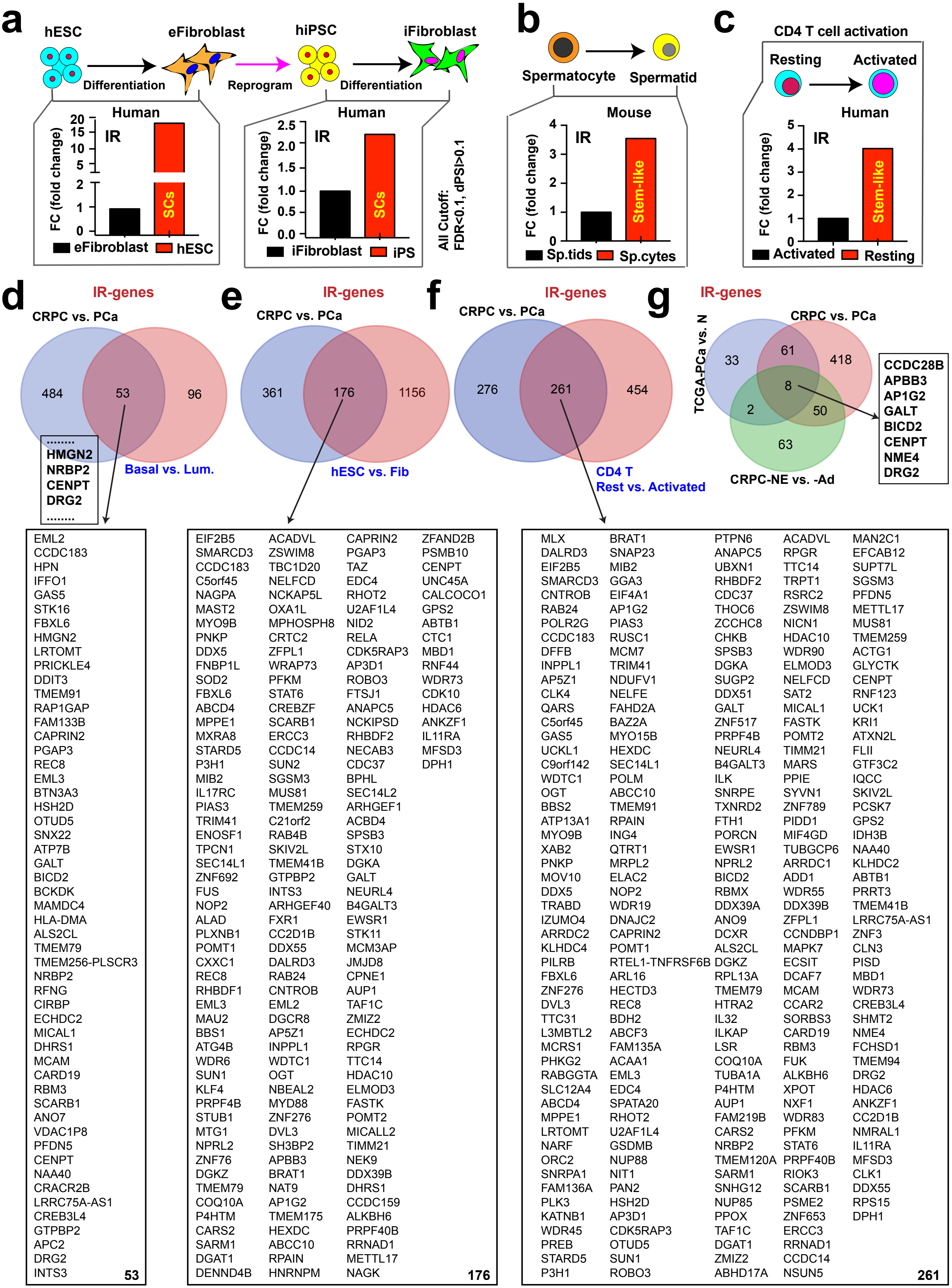

Supplementary Figure 5

Supplementary Figure 5. IR upregulation is a consistent hallmark of PCa development and progression and associated with stemness and aggressiveness.

(**a-c**) IR is a hallmark of undifferentiated stem/progenitor cells. Shown are bar graphs comparing the level of IR in human ESC or iPS cells relative to the respective fibroblasts (eFibroblasts, ESC-derived fibroblasts; iFibroblasts, iPS cell derived fibroblasts) (GSE73211, **a**), in spermatocytes vs. spermatids (GSE95138, **b**), and in resting vs. activated CD4 T cells (SRP058500, **c**).

(**d-f**) Overlapping of IR-affected genes identified in CRPC-Ad (vs. pri-PCa) and the above-mentioned contexts associates IR with stemness. Shown are Venn Diagrams presenting the overlapped IR genes identified in CRPC-Ad vs. human prostatic basal/stem cells (**d**), human ESC before and after differentiated into Fibroblast (**e**), and stem-like resting human CD4 T cells after activation (**f**). The actual overlapped genes are presented in respective boxes (below).

(**g**) PCa stage-specific repertoire of IR-affected genes. Shown is Venn Diagram highlighting the overlapping relationship of IR-parental genes identified in three large clinical datasets. The 8 IR genes commonly shared among the 3 comparisons are indicated on the right.

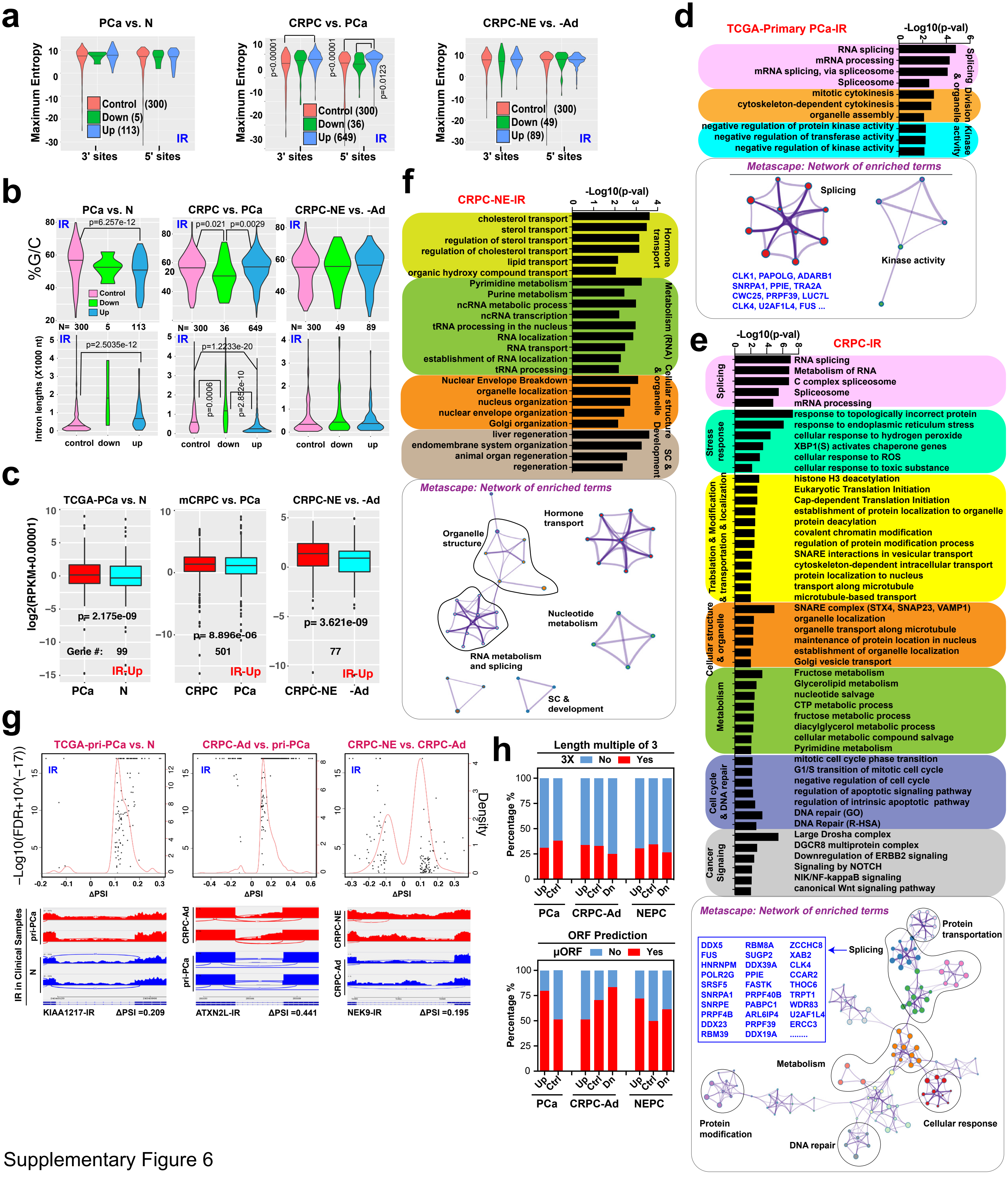

Supplementary Figure 6

Supplementary Figure 6. IR impacts PCa biology.

(**a** and **b**) IR code. Shown are splice site strength (**a**) and GC content and intron length (**b**) analyses of the introns spliced either aberrantly (Up and Down) or constitutively (Control). Asterisks indicate *p<0.05 and **p<0.001 using one-sided Mann-Whitney U tests after Bonferroni correction.

(**c**) Genes with IR tend to be expressed at higher levels. The log2(RPKM+0.00001) values were used to compare the expression of genes (number indicated) with upregulated IR events during PCa development and progression. Genes exhibiting both up- and down-regulated IR events were removed. In the plots, the center lines represent median values, box edges are 75th and 25th percentiles, and whiskers denote the maximum and minimum values, respectively. Significance was calculated using two-tailed paired Student's *t*-test.

(**d-f**) IR-impacted genes are associated with stages of PCa development and progression. Shown are GO analyses of the genes with upregulated IR, revealing the impact of global IR increase on PCa biology. Genes showing increased IR in pri-PCa (vs. normal tissues) (**d**), CRPC (vs. pri-PCa) (**e**), and CRPC-NE (vs. CRPC-Ad) (**f**) were used as input for Metascape analysis. Top enriched GO terms were displayed and grouped into functional categories. Several major categories were highlighted on the network of enriched terms. P-values were calculated based on the cumulative hypergeometric distribution.

(**g**) Density plots (upper) showing the distribution of FDR and ΔPSI of differentially expressed IR events observed in the indicated clinical RNA-seq datasets. Examples of IR events (with mapped reads from two samples of each group) in human clinical PCa tissues were illustrated (bottom).

(**h**) Analysis of coding potential of the retained introns. Shown are the percentage of retained introns that are divisible by 3 (upper) and the percentage of retained introns that possibly harbor a peptide-coding region based on the presence of a start codon 'ATG' in a DNA sequence minimal of >75nt in length (bottom).

a

Sample	Reads (M)	% Mapped
LNCAP-AD-1	98.629	96.391
LNCAP-AD-2	81.831	96.223
LNCAP-AI-1	84.592	96.393
LNCAP-AI-2	75.856	94.253
LNCAP-Enza-1	81.151	95.932
LNCAP-Enza-2	75.791	96.532
LNCAP-AI+DHT-1	76.713	95.308
LNCAP-AI+DHT-2	80.500	96.649
LNCAP-siNC-1	86.838	96.183
LNCAP-siNC-2	77.323	96.320
LNCAP-siAR-1	81.506	95.853
LNCAP-siAR-2	96.392	96.057

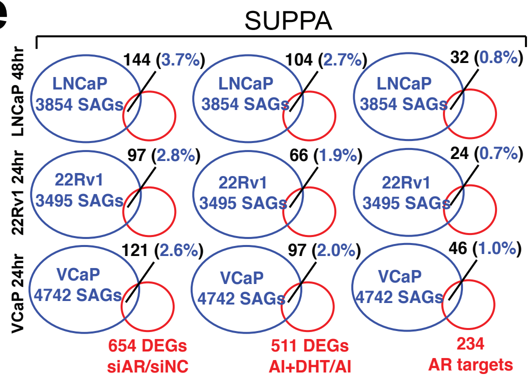

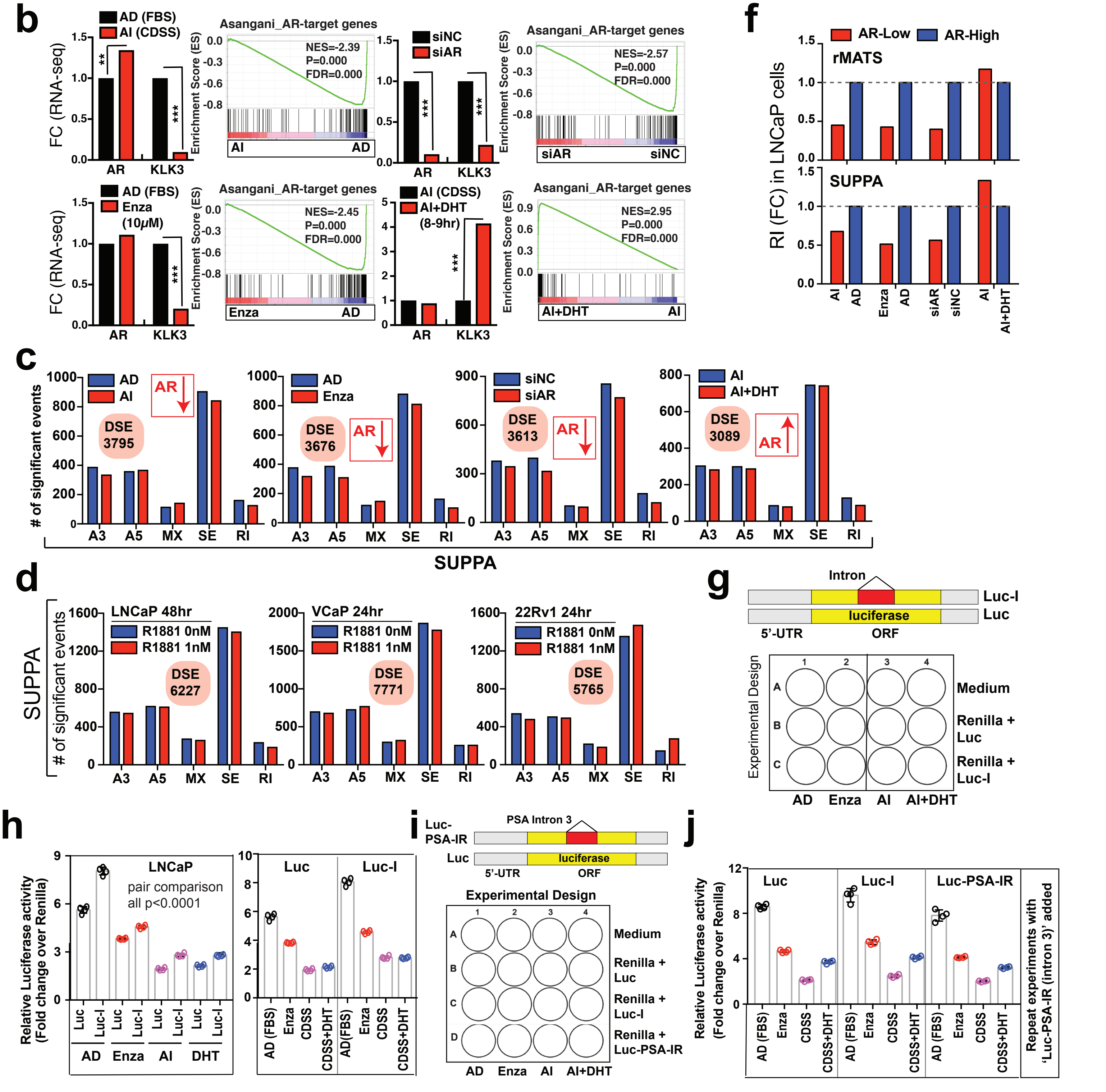

Supplementary Figure 7

Supplementary Figure 7. AR regulates PCa-associated AS globally, but not IR specifically.

(**a**) Statistics of RNA-seq data showing high quality of our data, as evidenced by both high sequencing depth (83.1 million reads per sample) and mapping rate (averaging 96%).

(**b**) Expression of AR and KLK3 and GSEA of an AR-target gene signature showing proper manipulations of endogenous AR signaling in LNCaP cells. AD, androgen-dependent; AI, androgen-independent; Enza, enzalutamide; DHT, dihydrotestosterone. FDR values were calculated by DESeq2 v.1.18.1.

(**c**) DSEs associated with high or low AR activity in LNCaP cells detected by SUPPA. Note that rMATS (Fig. 3i) and SUPPA generated similar patterns of splicing changes.

(**d**) Bar graphs showing SUPPA results of the DSEs associated with low or high AR activity in three androgen-sensitive cell lines (LNCaP, VCaP and 22Rv1) following 24 h or 48 h of R1881 treatment (GSE71797).

(**e**) Overlap between SAGs identified in three AR^+ cell lines (as in d) and three sets of AR-regulated genes in indicated contexts. The number in parentheses denotes the percentage of overlapped genes proportioned to all SAGs. The circle was not drawn to scale.

(**f**) Changes of the ratio of IR events detected by rMATS and SUPPA in indicated pairwise comparisons.

(**g**-**j**) AR signaling does not alter IR as assessed by splicing reporter assays. Shown are schematic diagram of the β-globin intron-containing (Luc-I, **g**), PSA intron 3-containing (**i**) and intronless (Luc) luciferase reporters, experimental design (**g** and **i**), and results of the dual luciferase assays (**h** and **j**). An IR event of PSA intron 3 has been reported (see Text). Results indicate that the pattern of luciferase activities across indicated contexts was not affected by insertion of introns from either non-AR target gene (e.g., β-globin) or AR target gene (e.g., PSA), suggesting that modulation of AR activity does not regulate IR specifically. ORF, open reading frame. Results (mean ± SD) were representative data of 3 independent experiments with 4 technical repeats for each experiment. The p values were calculated using two-tailed unpaired Student's *t*-test test.

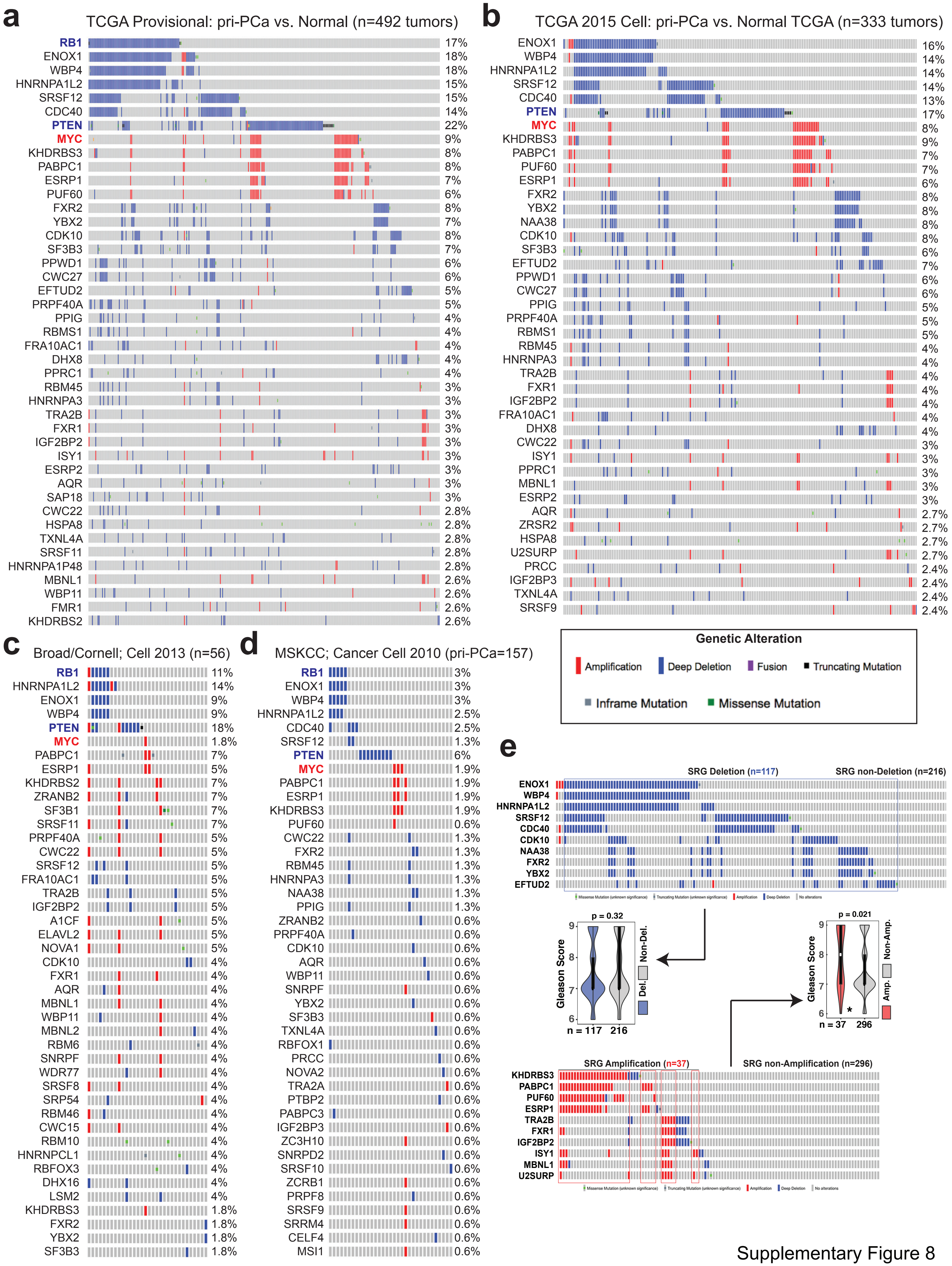

Supplementary Figure 8

Supplementary Figure 8. Prominent SRG deletions in pri-PCa and correlation of SRG amplifications with Gleason grade.

(**a-d**) Shown are the top 40 mutated SRGs in 4 indicated pri-PCa cohorts. The frequently deleted tumor suppressor (TS) genes, PTEN and RB1, and amplified oncogene, MYC, were included as references.

(**e**) Group analysis of top altered SRGs showing that deletion of SRGs (upper) did not, whereas amplification of SRGs (bottom) did, associate with increased Gleason grade. TCGA-PCa cohort (n=333) was divided based on the genomic features (i.e., deletion or amplification as indicated) of SRGs. In the plots, the center white dots represent median values, box edges are 75th and 25th percentiles, and whiskers denote the maximum and minimum values, respectively. The p value was calculated using two-tailed unpaired Student's *t*-test. $*p < 0.05$.

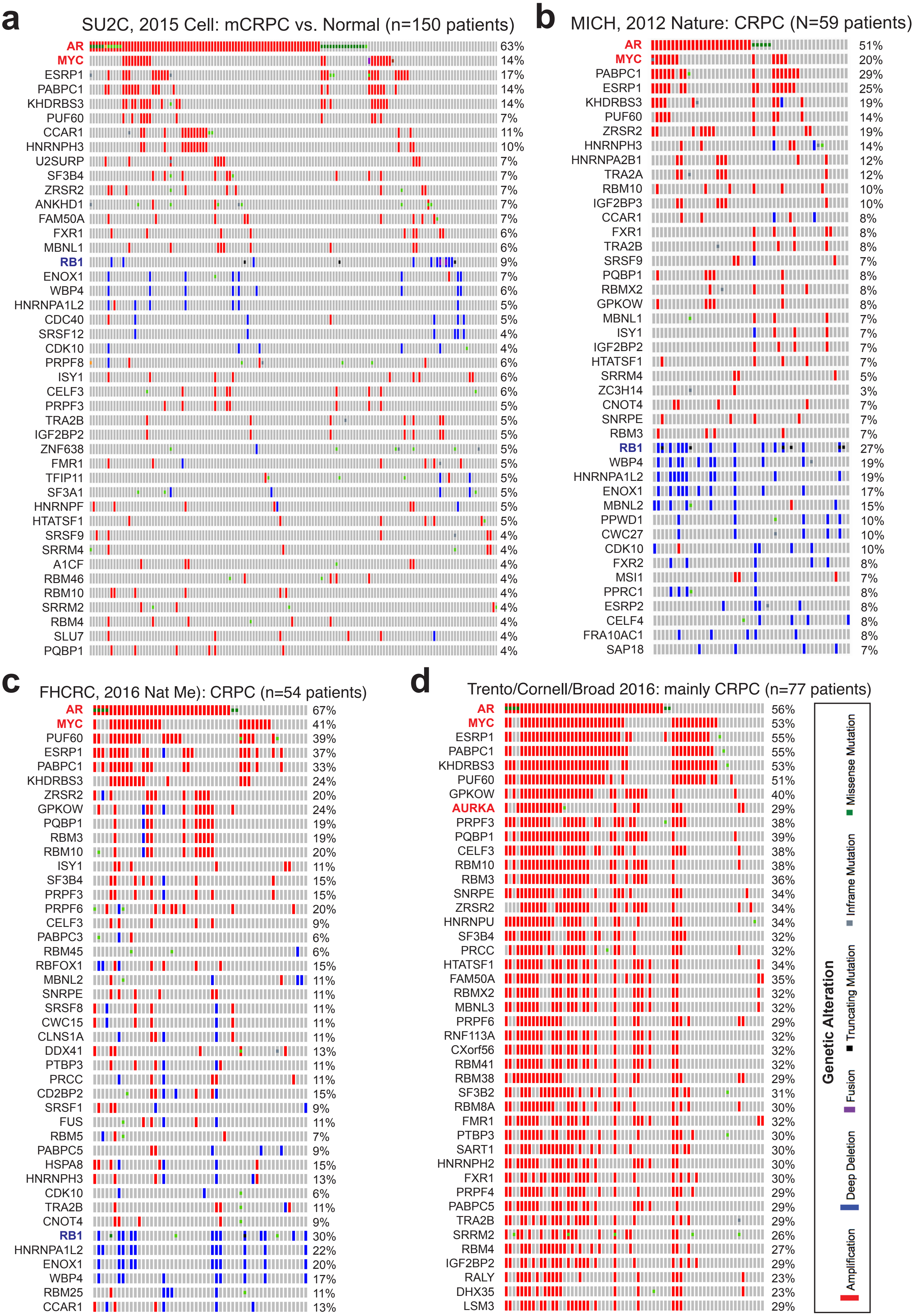

Supplementary Figure 9

Supplementary Figure 9. Prominent amplifications of SRGs in CRPC.

(**a-d**) Shown are the top 40 mutated SRGs in 4 indicated CRPC cohorts. The frequently deleted tumor suppressor RB1 and amplified oncogenes (AR, MYC and AURKA; highlighted in red) were included as references.

a

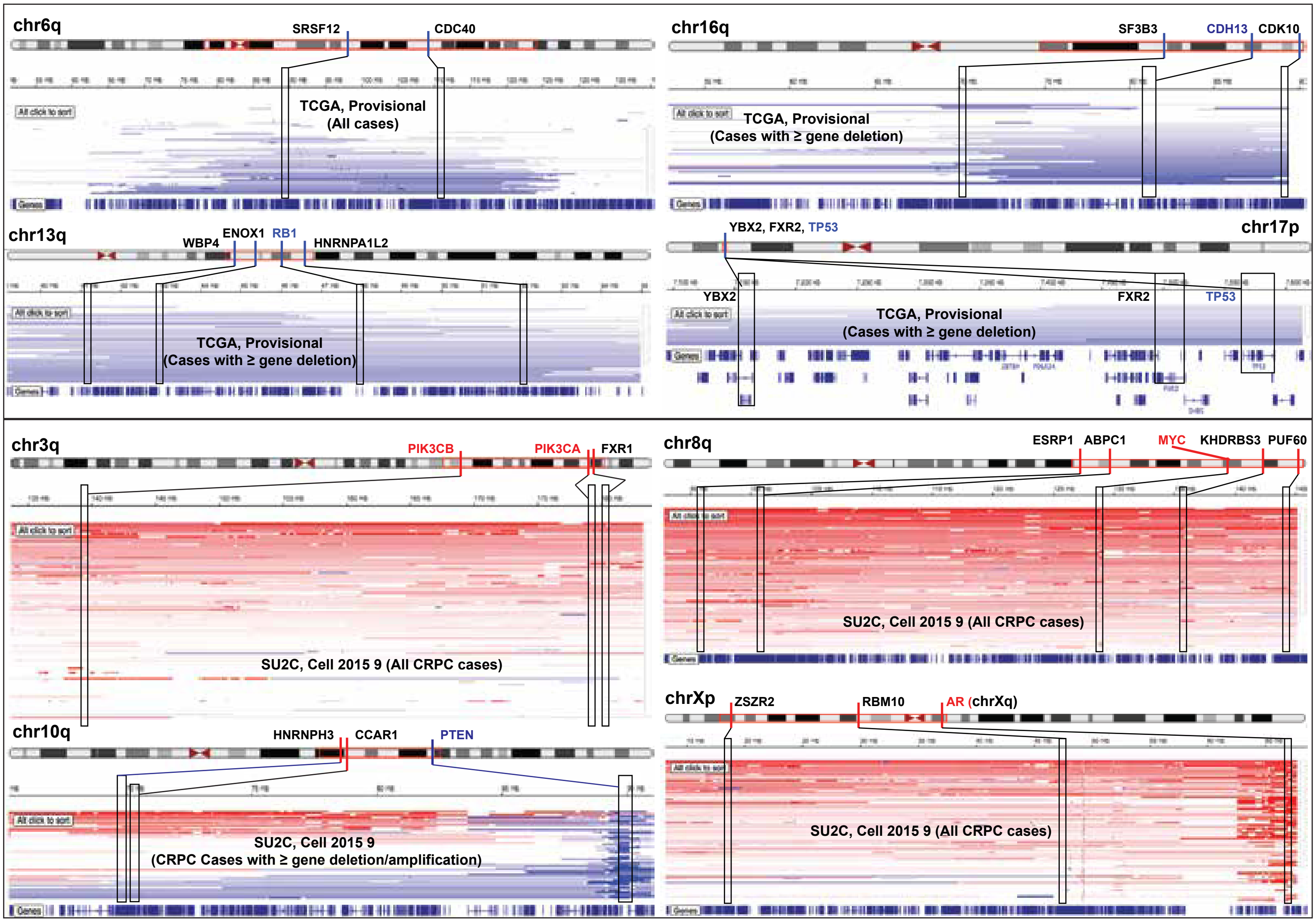

b

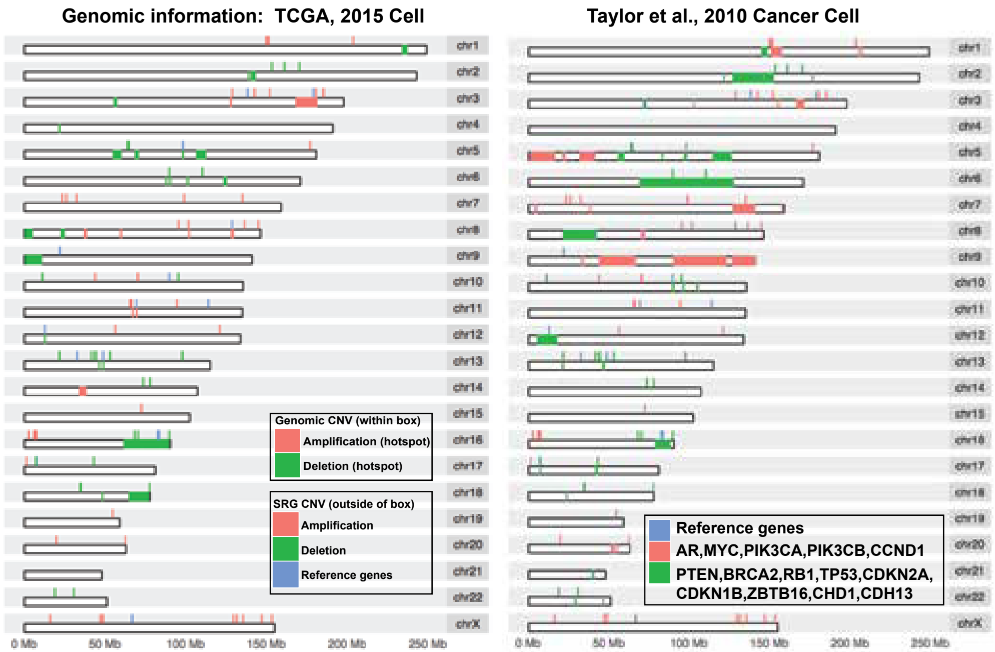

Genes with mutation rate (mainly ampl. or del.) >5%

chr1 SF3B4	chr5 DDX41	chr10 CCAR1
chr1 PRPF3	chr6 SRSF12	chr10 PTEN
chr1 CELF3	chr6 CDC40	chr10 FRA10AC1
chr1 SNRPE	chr7 IGF2BP3	chr11 SART1
chr2 PRPF40A	chr7 TRA2A	chr11 SF3B2
chr2 RBMS1	chr7 HNRNPA2B1	chr11 RBM4
chr2 PPIG	chr7 LSM5	chr11 CCND1
chr3 ISY1	chr7 BUD31	chr11 CWC15
chr3 PIK3CB	chr7 CNOT4	chr11 ZBTB16
chr3 U2SURP	chr8 ESRP1	chr12 CDKN1B
chr3 MBNL1	chr8 PABPC1	chr12 ZC3H10
chr3 PIK3CA	chr8 MYC	chr12 SRSF9
chr3 FXR1	chr8 KHDRBS3	chr13 SAP18
chr3 IGF2BP2	chr8 PUF60	chr13 BRCA2
chr3 TRA2B	chr9 CDKN2A	chr13 WBP4
chr5 CWC27	chr10 CELF2	chr13 ENOX1
chr5 PPWD1	chr10 HNRNPF	chr13 RB1
chr5 CHD1	chr10 HNRNPH3	chr13 HNRNPA1L2
chr13 MBNL2	chr19 LENG1	
chr14 RBM25	chr20 CRNKL1	
chr14 SNW1	chr20 PRPF6	
chr15 CELF6	chr22 ESS2	
chr16 SRRM2	chr22 SF3A1	
chr16 RBFOX1	chrX ZRSR2	
chr16 ESRP2	chrX RBM10	
chr16 SF3B3	chrX RBM3	
chr16 CDH13	chrX PQBP1	
chr16 CDK10	chrX GPKOW	
chr17 PRPF8	chrX AR	
chr17 YBX2	chrX RBMX2	
chr17 FXR2	chrX MBNL3	
chr17 TP53	chrX HTATSF1	
chr17 NAA38	chrX FMR1	
chr17 EFTUD2	chrX FAM50A	
chr18 CELF4		
chr18 TXNL4A		

Reference genes

chr3: PIK3CB, PIK3CA
chr5: CHD1
chr8: MYC
chr9: CDKN2A
chr10: PTEN
chr11: CCND1, ZBTB16
chr12: CDKN1B
chr13: BRCA2, RB1
chr16: CDH13
chr17: TP53
chrX: AR

Gene position on chr: Left to right

Supplementary Figure 10

Supplementary Figure 10. Genomic landscape of SRG alterations in PCa.

(**a**) Examples of the proximal chromosomal location of frequently deleted and amplified SRGs with well-known alteration hotspots encompassing tumor suppressors (e.g., CDH13, RB1, PTEN, TP53) or oncogenes (e.g., MYC, PIK3A, PIK3CB, AR), respectively.

(**b**) Chromosomal distribution of SRGs mutated at a frequency of >5% (in either one of the eight cohorts examined) showing that, except for the genes located in the hotspots, the majority of SRGs are mutated focally.

a

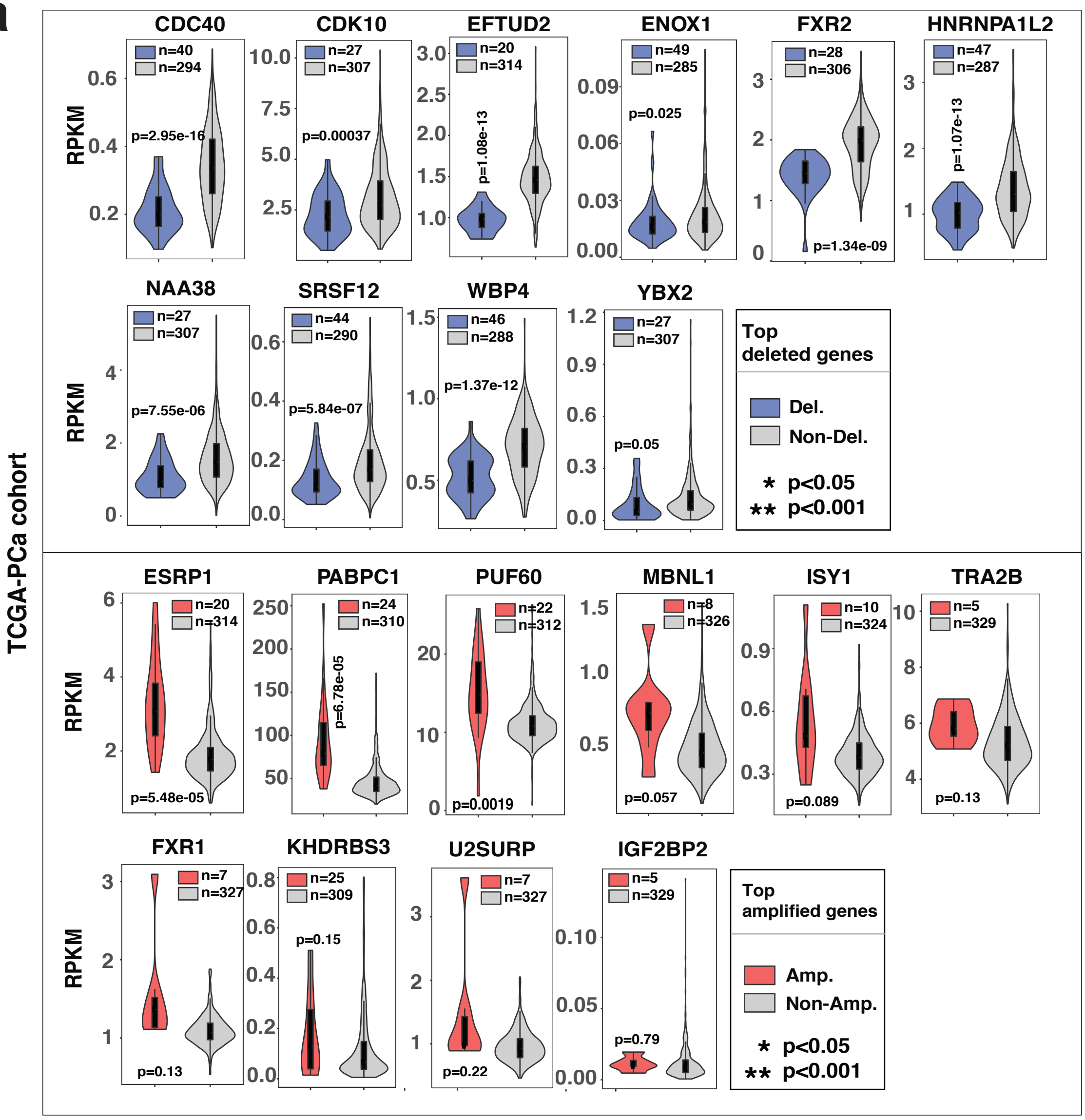

b

mCRPC cohort

CNVs affect gene expression

Gene	RNA-seq #	p-value	Type
ENOX1	3	0.003849453	DEL
WBP4	3	0.012860914	DEL
CDC40	8	0.018254283	DEL
HNRNPA1L2	5	0.258079555	DEL
SRSF12	9	0.640682261	DEL
PABPC1	15	0.043903076	AMP
FAM50A	10	0.06312572	AMP
PRPF3	8	0.087943654	AMP
FXR1	7	0.098960544	AMP
ESRP1	12	0.108094757	AMP
SF3B4	8	0.117212903	AMP
KHDRBS3	10	0.119235144	AMP
ZRSR2	7	0.18317957	AMP
PUF60	7	0.200696445	AMP
IGF2BP2	4	0.210212995	AMP
U2SURP	8	0.215973172	AMP
MBNL1	3	0.354095527	AMP
TRA2B	4	0.560800179	AMP
CCAR1	3	0.578379982	AMP
CELF3	8	0.68482243	AMP
ISY1	6	0.706287595	AMP
HNRNPH3	3	0.832754025	AMP

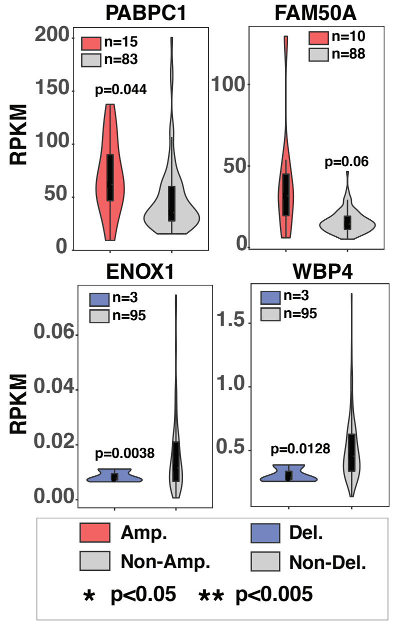

Supplementary Figure 11

Supplementary Figure 11. Copy number variations (CNVs) in SRGs correlate with gene expression.

Shown are the top deleted and amplified SRGs in TCGA (**a**) and CRPC (**b**) cohorts, which were selected based on their CNV status. Samples with or without SRG alterations individually were compared for gene expression. Note that fewer deletions and more amplifications were found for SRGs in CRPC cohort. Although not all amplified genes were overexpressed significantly in many cases due to a small sample size, the trend of increased expression was observed. In the plots, the center white dots represent median values, box edges are 75th and 25th percentiles, and whiskers denote the maximum and minimum values, respectively. The significance was calculated by two-tailed Student's *t*-test.

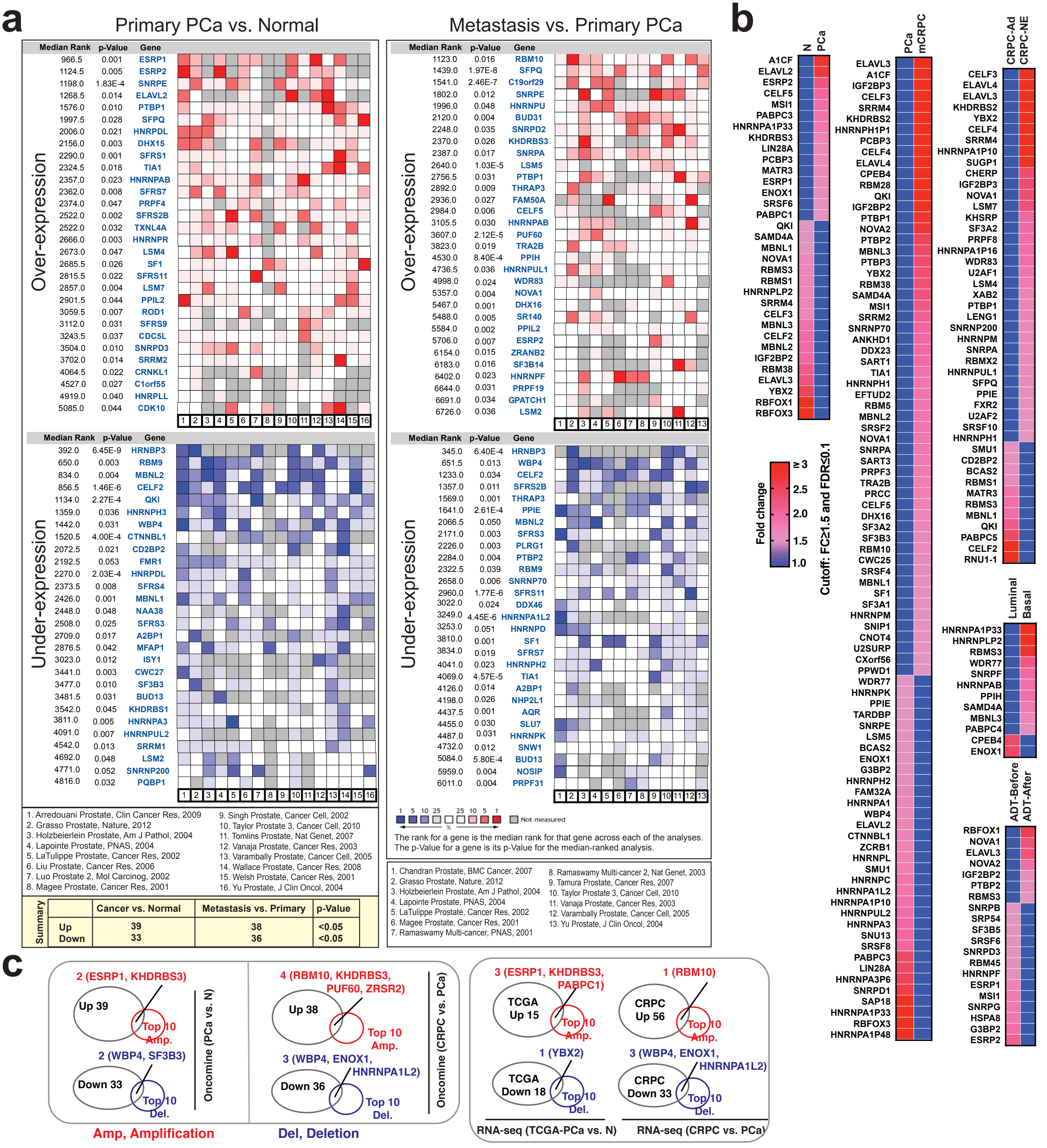

Supplementary Figure 12

Supplementary Figure 12. Pervasive SRG dysregulation during PCa development and progression.

(**a**) Oncomine concept analysis of the 274 SRGs showing up- or down-regulation in the expression of many SRGs in pri-PCa (vs. normal prostates) and metastasis (vs. pri-PCa), respectively. The medium-rank of <2500, <4000, and >4000 for a gene denotes high, moderate and low levels of expression, respectively. The rank for a gene is the median rank for that gene across each of the analyses. The p-value for a gene is its p-value for the median-ranked analysis.

(**b**) Comparative RNA-seq analyses showing differentially expressed SRGs across datasets that represent different stages of PCa development, therapy resistance, progression, and plasticity.

(**c**) Overlap of dysregulated SRGs identified by Oncomine (left) and RNA-seq (right) with top 10 deleted and top 10 amplified SRGs showing that copy-number variations (CNVs) often predict an over-expression or a down-expression at the population level, respectively.

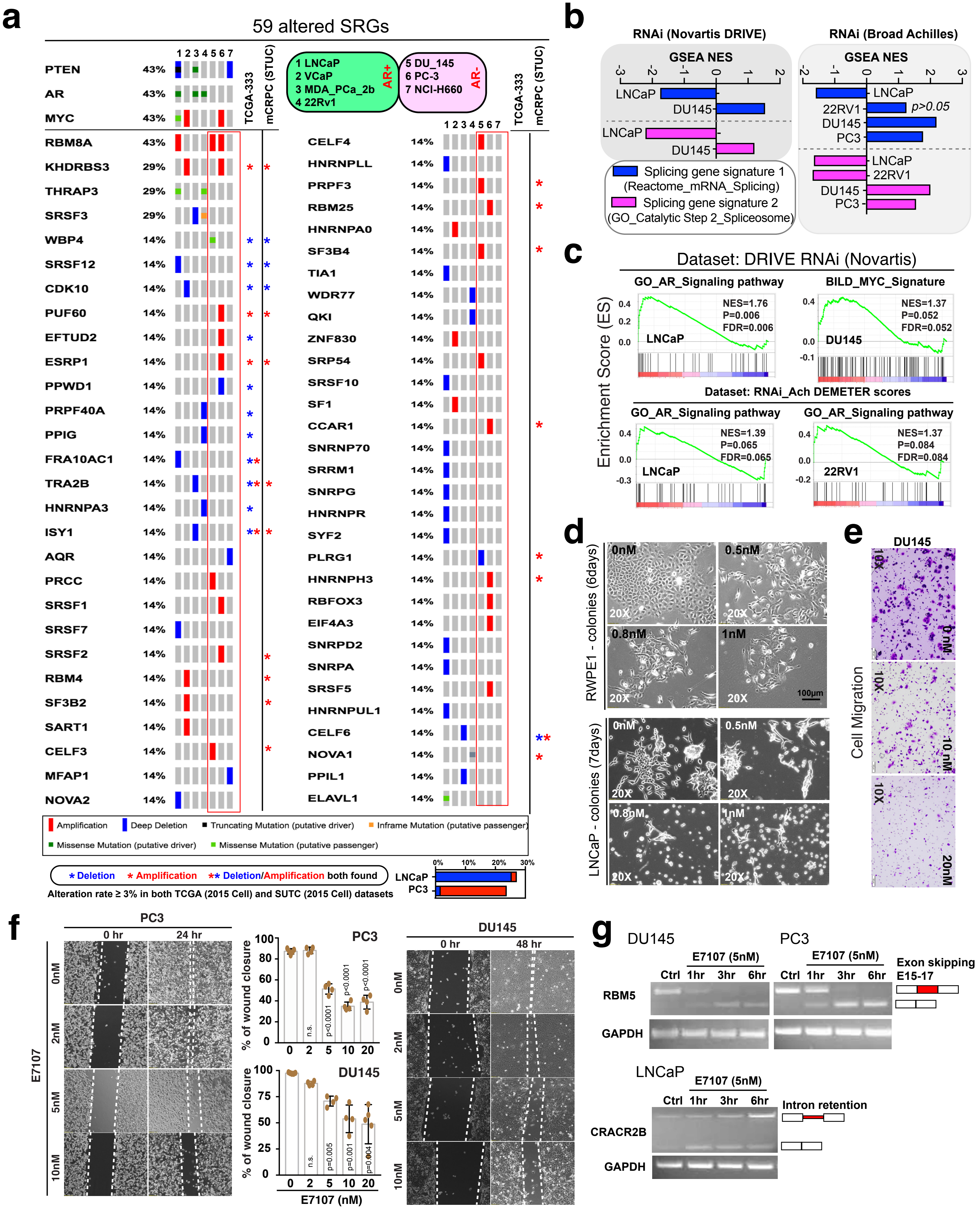

Supplementary Figure 13

Supplementary Figure 13. Aggressive PCa cells are susceptible to the spliceosome inhibitor E7107.

(**a**) Mutational landscape of the 59 SRGs in the indicated PCa cell lines. The results demonstrate that (differentiated) AR^+ LNCaP cells possess more gene deletions whereas (undifferentiated) AR^- PC3 cells have more gene amplifications resembling pri-PCa and CRPC, respectively.

(**b**) Summary of GSEA of two splicing gene signatures on ranked lists of essential genes identified in the indicated cell lines by two large-scale RNAi screening projects. The NES (normalized enrichment score) suggests that biologically aggressive PCa cells more preferentially rely on spliceosome activities.

(**c**) Representative GSEA of an AR and a MYC gene signature showing biological dependency of AR^+ LNCaP and 22Rv1 lines on AR signaling, and AR^- and more aggressive DU145 line on MYC signaling. The FDR for GSEA is the estimated probability that a gene set with a given NES represents a false positive finding, and an FDR < 0.25 is considered statistically significant.

(**d**) Images of benign prostatic epithelia RWPE1 and PCa LNCaP cells growing in medium containing varying concentrations of E7107 for 6-7 days in vitro.

(**e**) Representative images (original magnifications: 10x) of trans-well migration assays in DU145 cells treated with varying concentrations of E7107.

(**f**) E7107 inhibits PCa cell migration as analyzed by wound-healing assays. Results shown were representative of 2-3 independent experiments, with each experiment containing 3-5 technical repeats (mean ± S.D). For PC3, n=5 for the 0 nM group and n=4 for all other groups. For DU145, n=5 for 0 and 20 nM groups and n=4 for all other groups. P values were calculated using two-tailed unpaired Student's *t*-test. No significant cell death was observed in cells treated with E7107 for 24 or 48 h.

(**g**) Time-course study of the effect of E7107 on RNA splicing. Shown are representative RT-PCR analyses of SE in RBM5 and IR in CRACR2B genes in PCa cells exposed to either DMSO (Ctrl) or E7107 (5 nM). For SE in RBM5 in DU145 and PC3 cells (top), the upper (with SE) and lower bands were approximately 240 and 160 bp, respectively. For IR in CRACR2B in LNCaP cells (bottom), the sizes of the upper (with IR) and lower bands were about 386 and 103 bp, respectively. GAPDH was 148 bp.

The experiment was repeated two times independently with similar results obtained (**d**, **e**, **g**).

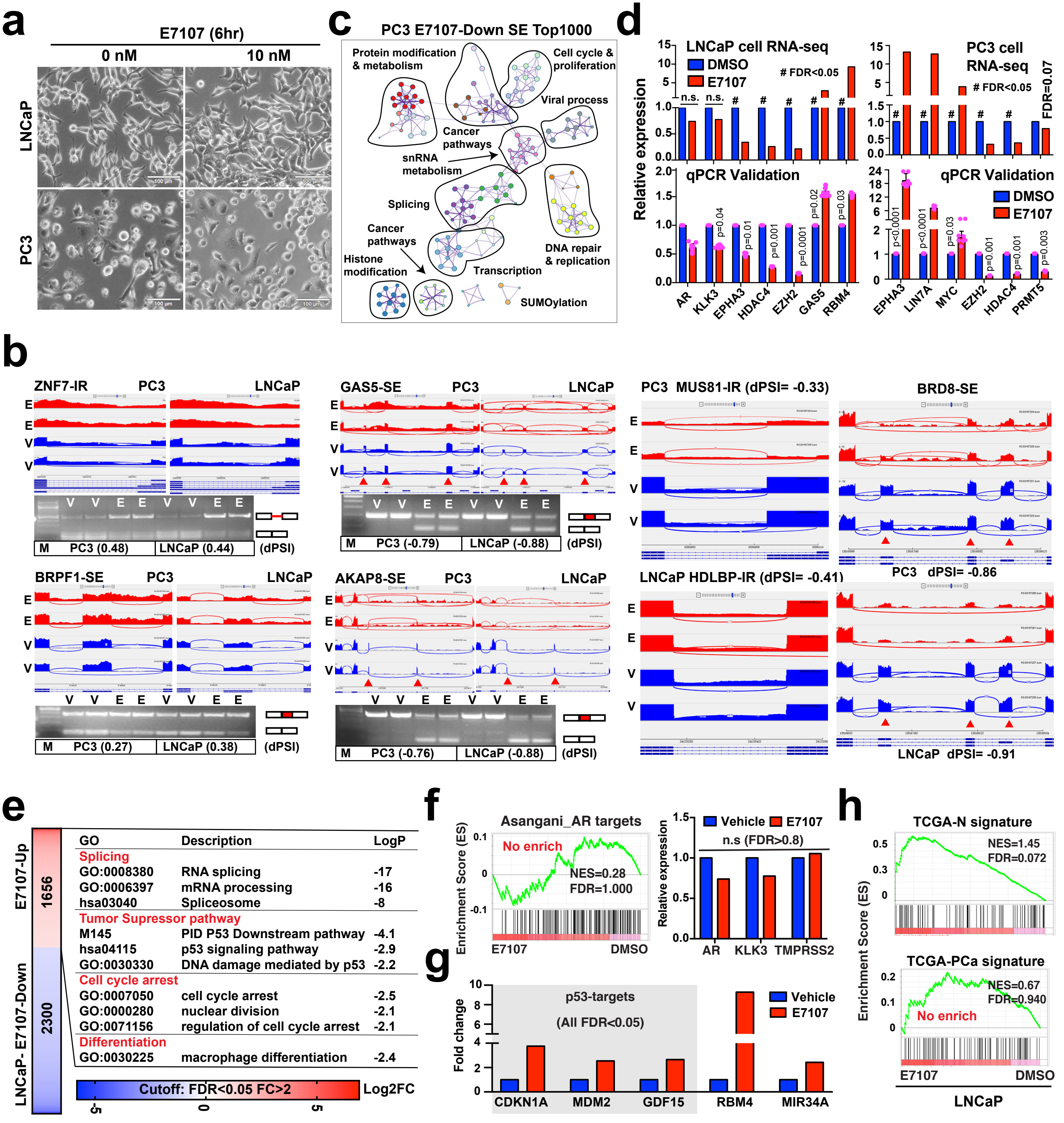

Supplementary Figure 14

Supplementary Figure 14. The E7107 compound inhibits PCa cells via targeting spliceosome.

(**a**) No obvious cytotoxicity was observed in PCa cells treated with E7107 (10 nM) for 6 hr in vitro. The experiment was repeated two times independently with similar results.

(**b**) E7107 modulates RNA splicing. Shown are Sashimi plots of the indicated AS events (with mapped reads from two replicates of each group) and RT-PCR validation in indicated PCa lines treated with E7107 (10 nM, 6 hr). The event ΔPSI values calculated by rMATS were provided in parentheses. The red triangle denotes aberrantly spliced region. E, E7107; V, vehicle. The experiment was repeated two times independently with similar results. For RT-PCR gel images (below), the sizes for ZFN7-IR were ~511 and 124 bp, GAS5-SE 172 and 88 bp, BRPF1-SE 376 and 91 bp, and AKAP8-SE 443 and 162 bp, respectively.

(**c**) GO analysis of top 1000 genes exhibiting down-regulated SE events after E7107 treatment in PC3 cells. E7107 dramatically inhibited the SE, the most abundant splicing type. These PC3-specific SE events abolished by E7101 were chosen for GO analysis to reveal the impact of E7107-induced splicing alterations on PCa biology.

(**d**) qRT-PCR validation of expression of the indicated genes showing consistency with RNA-seq data. The upper panels show the fold change of gene expression calculated by DESeq of RNA-seq data. In bottom panels, gene expression in DMSO group was set as onefold and the relative quantification of gene expression was analyzed by normalization to internal *GAPDH* mRNA level. The error bars represent the mean±S.D (n=3). P values were calculated using two-tailed unpaired Student's *t*-test.

(**e**) GO analysis of genes upregulated at bulk RNA levels in LNCaP cells after E7107 (10 nM, 6h) treatment in vitro. Significantly enriched terms with similar descriptions and functions were grouped into specific biological categories to better reflect the biology of the context.

(**f**) AR signaling was not affected by E7107. Shown are GSEA of an AR gene signature (left) and the expression of AR and AR target genes (right).

(**g**) Upregulation of p53 targets and tumor suppressors (RBM4 and MIR34A) in LNCaP cells after E7107 treatment.

(**h**) GSEA showing enrichment of TCGA-normal specific, but not tumor specific, gene signature in E7107 treated LNCaP cells, indicating a reversal of tumor aggressiveness. An FDR<0.25 is considered to be statistically significant for GSEA.

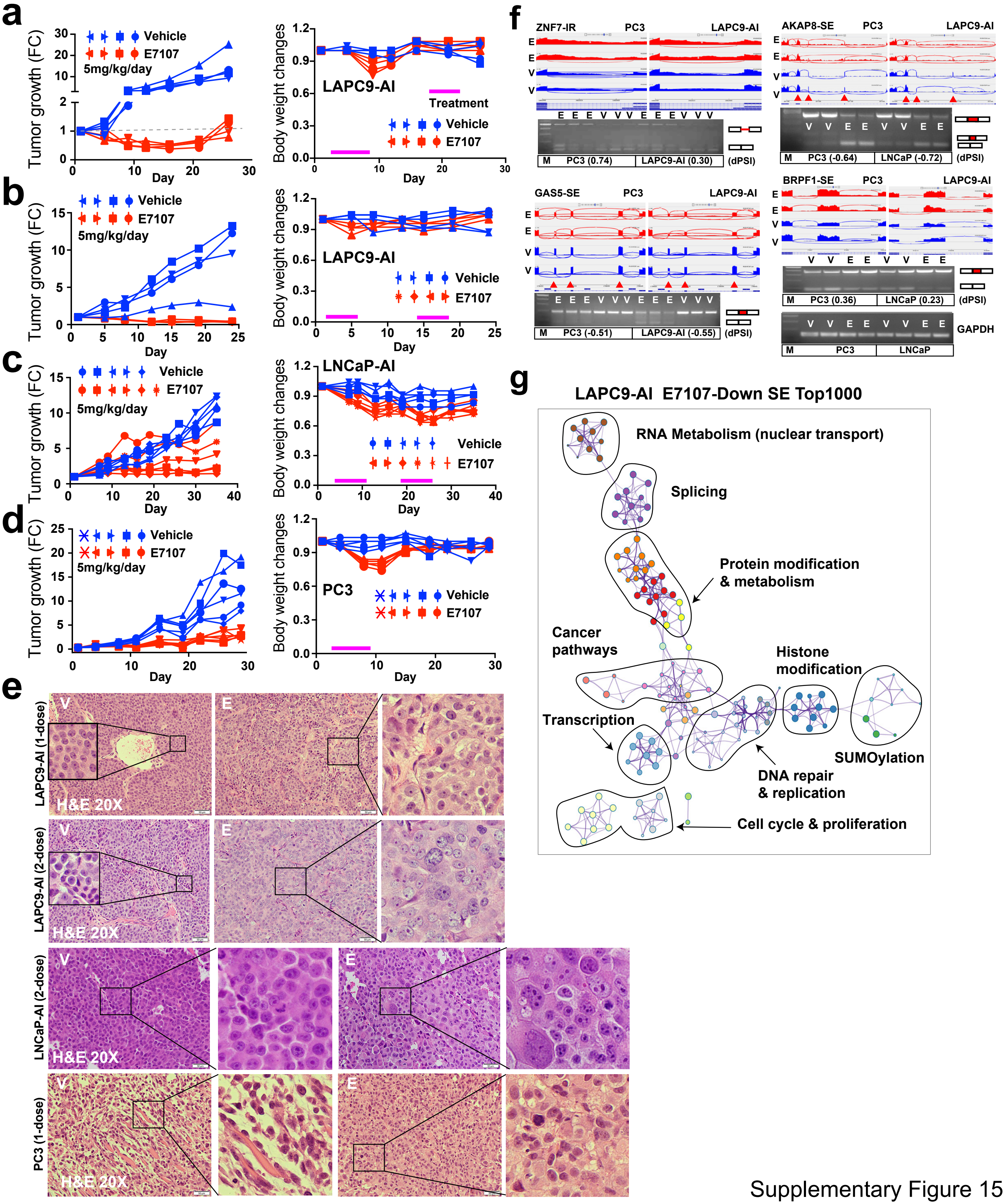

Supplementary Figure 15

Supplementary Figure 15. E7107 inhibits CRPC in vivo.

(**a-d**) In vivo effects of E7107 treatment on tumor growth (left) and body weight of tumor-bearing mice (right) in the indicated CRPC models. The pink lines in the right panels indicate the treatment regimens (see Fig. 9b-e). For **a** and **b**, n=4 for each group. For **c**, n=5 and 6 for vehicle and treatment group, respectively. For **d**, n=5 for each group.

(**e**) E7107 treatment promoted more differentiated morphologies in the CRPC models. Shown are hematoxylin and eosin (H&E) staining in endpoint tumors as above. All tumors (n indicated above) were examined. Boxed regions are enlarged. V, vehicle; E, E7107.

(**f**) E7107 treatment reshapes the AS landscape in treated CRPC models. Shown are Sashimi plots of the indicated AS events (with mapped reads from two replicates of each group) and RT-PCR validation in indicated models treated with E7107. The RNAs derived from all tumors (n indicated above) were used for RT-PCR with 3 (left panel) and 2 (right panel) samples from each group loaded in the gel. The event ΔPSI values calculated by rMATS were provided in parentheses. The red triangles denote aberrantly spliced region. E, E7107; V, vehicle. the sizes for ZFN7-IR were approximately 511 and 124 bp, AKAP8-SE 443 and 162 bp, GAS5-SE 172 and 88 bp, and BRPF1-SE 376 and 91 bp, respectively.

(**g**) GO analysis of the top 1000 genes exhibiting down-regulated SE events after E7107 treatment in LAPC9-AI tumors. E7107 dramatically inhibited the SE; hence, these LAPC9-AI specific SE events abolished by E7107 were chosen for GO analysis to reveal the impact of E7107-induced splicing alteration on CRPC biology.

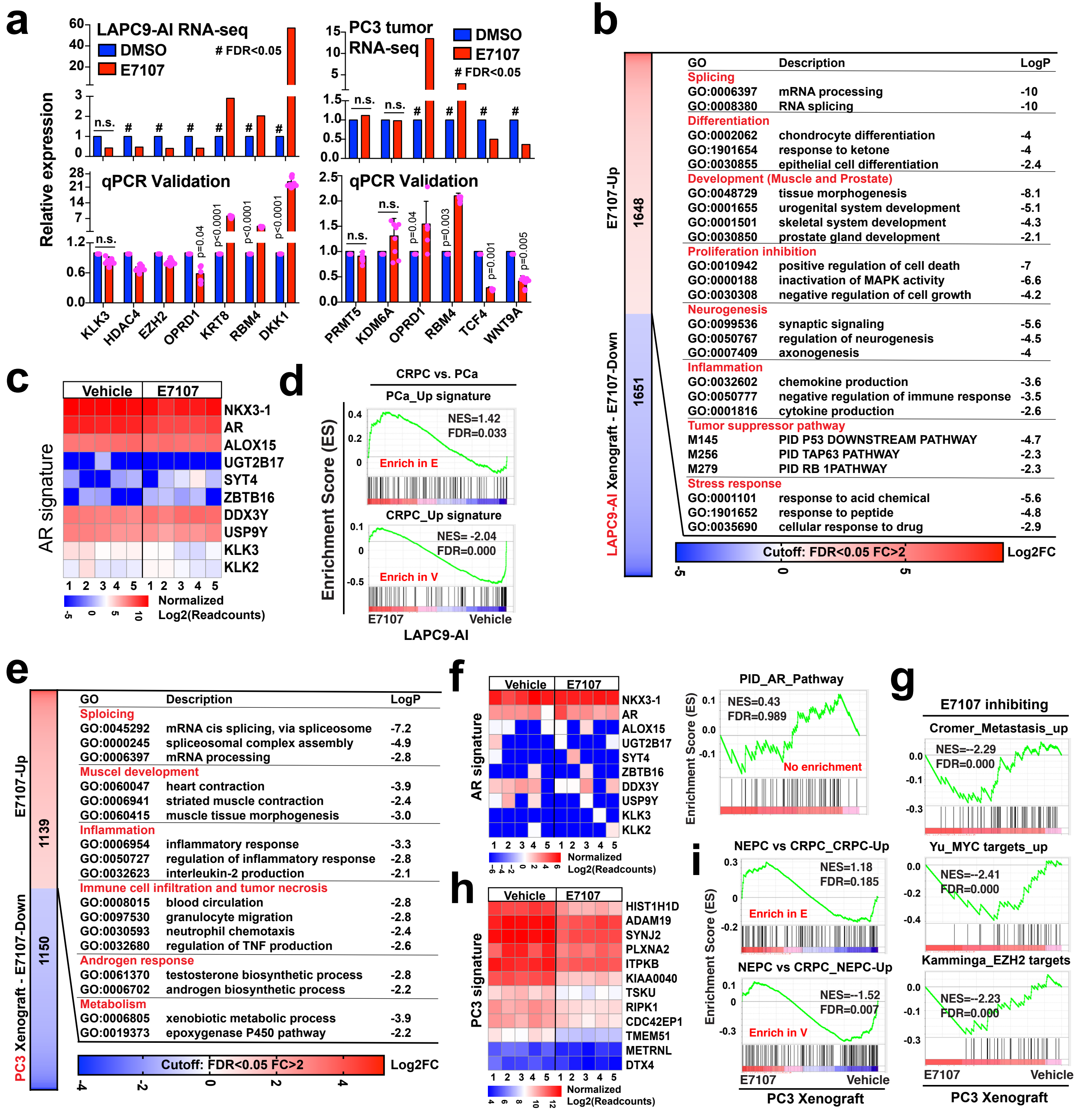

Supplementary Figure 16

Supplementary Figure 16. E7107 reverses the aggressiveness of PCa cells at the transcriptome level.

(**a**) qRT-PCR analysis of the indicated genes showing consistency with RNA-seq. The upper panels show the fold change of gene expression calculated by DESeq of RNA-seq data. In bottom panels, gene expression in DMSO group was set as baseline (i.e., 1) and the relative levels of gene expression was analyzed by normalization to internal *GAPDH* mRNA level. Error bars represent the mean±S.D (n=3). P values were determined using two-tailed unpaired Student's *t*-test. n.s, not significant.
(**b**) GO analysis of the genes upregulated at bulk RNA levels in LAPC9-AI tumors after E7107 treatment.
(**c**) Heatmap of AR signature showing that AR signaling was not affected by E7107 in LAPC9-AI tumors.
(**d**) GSEA showing enrichment of pri-PCa-specific and CRPC-specific (defined by comparing CRPC vs. PCa) gene signatures in E7107- and vehicle-treated LAPC9-AI tumors, respectively. As LAPC9-AI molecularly resembles CRPC-Ad, this result suggests that E7107 treatment reverses tumor aggressiveness at the molecular level.
(**e**) GO analysis of genes upregulated in PC3 tumors after E7107 treatment.
(**f**) Heatmap of AR signature (left) and GSEA of AR pathway (right) showing that AR signaling was not affected by E7107 (Note PC3 cells were negative for AR and NKX3-1 proteins but expressed these genes at mRNA levels).
(**g**) GSEA showing negative enrichment of metastasis and stemness-related signatures in E7107-treated relative to vehicle-treated PC3 tumors (suggesting that inhibition of spliceosome activity abolishes the aggressive properties of CRPC in vivo).
(**h**) Heatmap showing reduced expression of a PC3 gene signature (defined as top genes exclusively expressed or over-expressed in PC3 compared with LNCaP) in E7107-treated PC3 tumors.
(**i**) GSEA showing enrichment of CRPC-Ad specific and CRPC-NE specific (defined by comparing CRPC-NE vs. -Ad) gene signatures in E7107- vs. vehicle-treated PC3 tumors. As PC3 tumors molecularly resemble clinical CRPC-NE, this result suggests that E7107 treatment reverses PCa aggressiveness.
