# Supplementary material for: Intron retention is a hallmark and spliceosome represents a therapeutic vulnerability in aggressive prostate cancer
Source: Nat Commun. 2020 Apr 29;11:2089. doi: 10.1038/s41467-020-15815-7 (PMC7190674; doi:10.1038/s41467-020-15815-7)
Supplement: Supplementary file 2 — Description of Additional Supplementary Files [file 41467_2020_15815_MOESM2_ESM.pdf]

Description of Additional Supplementary Files

File Name: Supplementary Data 1
Description: The mutational landscape of 274 SRGs in PCa

File Name: Supplementary Data 2
Description: Summary of RNA-seq datasets used in this study for splicing analyses

File Name: Supplementary Data 3
Description: Summary of statistically significant differentially spliced events (DSEs)

File Name: Supplementary Data 4
Description: Summary of SRG dysregulation

File Name: Supplementary Data 5
Description: Summary of the prognostic association of differentially expressed SRGs

File Name: Supplementary Data 6
Description: DSEs identified in PCa cells and xenografts treated with E7107

File Name: Supplementary Data 7
Description: DEGs identified in PCa cells and xenografts treated with E7107

File Name: Supplementary Data 8
Description: RBP motif analysis of IR

File Name: Supplementary Data 9
Description: List of primers used in this study
