# Supplementary material for: Intron retention is a hallmark and spliceosome represents a therapeutic vulnerability in aggressive prostate cancer
Source: Nat Commun. 2020 Apr 29;11:2089. doi: 10.1038/s41467-020-15815-7 (PMC7190674; doi:10.1038/s41467-020-15815-7)
Supplement: Supplementary file 11 — Supplementary Data 9 [file 41467_2020_15815_MOESM11_ESM.docx]

**Supplementary Data 9. Primers used in this study.**

Related to Fig. 3h; 7a; 8g; 9i and Supplementary Fig. 4a, 7i; 13g; 14b, d; 15f, 16a

| **qPCR Primers** | **Sequence (Forward 5’-3’)** | **Sequence (Reverse 5’-3’)** |  |  | |  |  |  |  |
| --- | --- | --- | --- | --- | --- | --- | --- | --- | --- |
| GAPDH | ACTTTGGTATCGTGGAAGGACT | GCCTTGGCAGCGCCAGTAG | | | |  | |  | |
| ACTB | CGTGGACATCCGCAAAGAC | GGAAGGTGGACAGCGAGGC | | | |  | |  | |
| KLK3 | GGGAGGGTCTTCCTTTGGCA | ATCTGAGGGTTGTCTGGAGGA | | | |  | |  | |
| AR | GAGAAGCCTTAGAATGGGTGG | TGGCTTATGGGATAGGACAAC | | | |  | |  |  |
| EPHA3 | ATGTTTCCAGACACGGTACC | CCATCTTCCTGAGTAGAACTGTGAGG | | | |  | |  | |
| HDAC4 | AATCTGAACCACTGCATTTCCA | GGTGGTTATAGGAGGTCGACACT | | | |  | |  | |
| EZH2 | TGCACATCCTGACTTCTGTGAGC | AGTCACTGGTCACCGAACACTC | | | |  | |  | |
| GAS5 | GCAGTAAGCTGCATGCTTGC | TTGCTTGGGTAAGGACATGA | | | |  | |  | |
| RBM4 | ACAAGCTTCATGGGGTGAAC | GACCGGACCATACTCCTCAA | | | |  | |  | |
| LIN7A | ATGCGAGAGATATAAATGGGGGAAT | AAACGATAACTGTTAATGGCTGTCC | | | |  | |  | |
| MYC | CGGTTTTCGGGGCTTTATCTAAC | AGTTTCGTGGATGCGGCAAG | | | |  | |  | |
| PRMT5 | CCATAACGGTACGTGAAGGC | TTGCCCACCTTGATGTAAGG | | | |  | |  | |
| OPRD1 | TTCGGCATCGTCCGGTACAC | CTTGGCACTCTGGAAAGGCA | | |  | |  | |  |
| KRT8 | GAGGTCAAGGCACAGTACGAG | CTTGGCGTTGGCATCCTTA | | |  | |  | |  |
| DKK1 | TTCTTCTAGGCTTCACACTTGTC | AACAAAGCTCTTACACTCCAGGT | | |  | |  | |  |
| KDM6A | CTATCTCGTAAGGCTGCTGG | TTTGTCAGATATATACAAAACATG | | |  | |  | |  |
| TCF4 | TCTCCATAGTTCCTGGACGG | GCGATGTTTTCACCTCCTGT | | |  | |  | |  |
| WNT9A | CAAGTATGAGACGGCACTCAA | GCAGAAGCTAGGCGAGTCATC | | |  | |  | |  |
| ESRP1 | TAGTACTGCTGCCTCATGTAAC | GATCTTAAGTGCCAGTTATCAC | | |  | |  | |  |
| KHDRBS3 | ATGAAGCTGGGACAGAAAGT | CATGTCCCATCCTGGCATA | | |  | |  | |  |
| **Splicing Primers** | **Sequence (Forward 5’-3’)** | **Sequence (Reverse 5’-3’)** |  |  | |  |  |  |  |
| RBM5-SE | CGGCTGTAGTGTCCCAGAGT | TTGCGAGTTGGGGTCATAAT | | | |  | |  | |
| CRACR2B-IR | GCTCTGTACGAGGAGACGGA | GCAGCTCCAGCTCCAGAC | | | |  | |  | |
| DDIT3-IR | CCAGGAGGTGAAACATAGG | GTATCTTCATACATCACCACA | | | |  | |  | |
| ZNF7-IR | TGCCCACCCACCACTGAG | CAGCATCACTTCCCTGTAGAG | | | |  | |  | |
| BRPF1-SE | CAAGGATGGGCAGACAGATAG | GTTTCCACCGCTGAAGCC | | | |  | |  | |
| GAS5-SE | GCAGTAAGCTGCATGCTTGC | TTGCTTGGGTAAGGACATGA | | | |  | |  | |
| AKAP8-SE | CTCGAACGGCTGGAAGCG | CTGTGGTCGCCTAGTAAACGG | | | |  | |  | |
| MUS81-RI | CACAGCAGACATTAAGGAGTCA | CTTATTCTTGATGGCTCCTGC | | | |  | |  | |
| ATP7B-SE | CTCACCAAGGGTCACAACG | GATACCCAGCAACGAGCC | | | |  | |  | |
| ATXN2L-IR | GCTACCACGCCTACTGGAAG | TGTTGGTGAAGCCGTGGG | | | |  | |  | |
| SYT7-SE  SYT7-SE1  SYT7-SE3 | TGGTGAGGGAGTTGACGAGG  CCTGAGCGTTCTGGTTCTGC  GGCCAGGTGGCTCTGCAC | ATTCCTTGGAGACGGTGGG  GAAGGCCGGATGGTGGTGC  CTGTCGGGCGCCAAAGTG | | | |  | |  | |
| UBE3A-SE | AATGTCGTCAGACTGAGGTTC | GATCACCCTGATGTCACCGA | | | |  | |  | |
| MXRA8-IR | CCCCTTTGACTTTCCCGACT | ACTGGCGCGCGGCCACAAC | | | |  | |  | |
| **Cloning** | **Sequence (Forward 5’-3’)** | **Sequence (Reverse 5’-3’)** | | | |  | |  | |
| CMV-LUC2P/PSA-intron/ARE fragment 1 | CTAGTTAAGCTTGGCAATCC | CCAGGCGTACCTGGTAGCCCT | | | |  | |  | |
| CMV-LUC2P/PSA-intron/ARE fragment 2 (PSA intron region) | CTACCAGGTACGCCTGGGCC | TGGGGCTACCTACGGGCCAGG | | | |  | |  | |
| CMV-LUC2P/PSA-intron/ARE fragment 3 (PSA intron region) | CCCGTAGGTAGCCCCAGCCG | CGCAAACGGATCCTTATCGA | | | |  | |  | |
| Sequencing for LUC2P/PSA-intron/ARE insert | AGGCTAAGGTGGTGGACTTG |  | | | |  | |  | |
| **Taqman qPCR** | **Supplier** | **Assay ID** | | | |  | |  | |
| GAPDH | Thermo Fisher Scientific | Hs00266705_g1 | | | |  | |  | |
| ESRP1 | Thermo Fisher Scientific | Hs00214472_m1 | | | |  | |  | |
| KHDRBS3 | Thermo Fisher Scientific | Hs00198065_m1 | | | |  | |  | |
