# Supplementary material for: Intron retention is a hallmark and spliceosome represents a therapeutic vulnerability in aggressive prostate cancer
Source: Nat Commun. 2020 Apr 29;11:2089. doi: 10.1038/s41467-020-15815-7 (PMC7190674; doi:10.1038/s41467-020-15815-7)
Supplement: Supplementary file 12 — Reporting Summary [file 41467_2020_15815_MOESM12_ESM.pdf]

nature research

Corresponding author(s): Dingxiao Zhang, Song Liu, Dean G. Tang

Last updated by author(s): Mar 2, 2020

Reporting Summary

Nature Research wishes to improve the reproducibility of the work that we publish. This form provides structure for consistency and transparency in reporting. For further information on Nature Research policies, see Authors & Referees and the Editorial Policy Checklist.

Statistics

For all statistical analyses, confirm that the following items are present in the figure legend, table legend, main text, or Methods section.

n/a	Confirmed	
☐	☒	The exact sample size (*n*) for each experimental group/condition, given as a discrete number and unit of measurement
☐	☒	A statement on whether measurements were taken from distinct samples or whether the same sample was measured repeatedly
☐	☒	The statistical test(s) used AND whether they are one- or two-sided<br>*Only common tests should be described solely by name; describe more complex techniques in the Methods section.*
☐	☒	A description of all covariates tested
☐	☒	A description of any assumptions or corrections, such as tests of normality and adjustment for multiple comparisons
☐	☒	A full description of the statistical parameters including central tendency (e.g. means) or other basic estimates (e.g. regression coefficient) AND variation (e.g. standard deviation) or associated estimates of uncertainty (e.g. confidence intervals)
☐	☒	For null hypothesis testing, the test statistic (e.g. *F*, *t*, *r*) with confidence intervals, effect sizes, degrees of freedom and *P* value noted<br>*Give P values as exact values whenever suitable.*
☒	☐	For Bayesian analysis, information on the choice of priors and Markov chain Monte Carlo settings
☒	☐	For hierarchical and complex designs, identification of the appropriate level for tests and full reporting of outcomes
☒	☐	Estimates of effect sizes (e.g. Cohen's *d*, Pearson's *r*), indicating how they were calculated

Our web collection on statistics for biologists contains articles on many of the points above.

Software and code

Policy information about availability of computer code

Data collection	rMATS v.4.0 and SUPPA v.2.2.0 were used for splicing mapping.
Data analysis	All visualization and associated statistical analysis were performed using R and GraphPad Prism 7. Bioinformatic analysis was conducted using the following tools and versions: GENCODE v.25, STAR2 v.2.5.2; DESeq2 v.1.18.1; Salmon v.0.7.2; rMATS v.4.0; SUPPA v.2.2.0; GSEA; GO, Metascape; Rsamtools for sequence extraction; RBPmap web service for RBP binding motif analysis; MaxEntScan web service for splice site strength analysis; Kaplan-Meier program and PrognoScan for survival analysis; Oncomine for gene expression analysis; IGV for RNA-seq data visualization. BD FACSDiva v8.0.1 software was used for flow cytometry analysis.

For manuscripts utilizing custom algorithms or software that are central to the research but not yet described in published literature, software must be made available to editors/reviewers. We strongly encourage code deposition in a community repository (e.g. GitHub). See the Nature Research guidelines for submitting code & software for further information.

Data

Policy information about availability of data

All manuscripts must include a data availability statement. This statement should provide the following information, where applicable:

- Accession codes, unique identifiers, or web links for publicly available datasets
- A list of figures that have associated raw data
- A description of any restrictions on data availability

All data is available in the main text and/or the supplementary materials. RNA-seq data have been deposited in GEO database under accession code GSE114052, GSE128515 and GSE139962.

Field-specific reporting

Please select the one below that is the best fit for your research. If you are not sure, read the appropriate sections before making your selection.

☒ Life sciences ☐ Behavioural & social sciences ☐ Ecological, evolutionary & environmental sciences

For a reference copy of the document with all sections, see nature.com/documents/nr-reporting-summary-flat.pdf

Life sciences study design

All studies must disclose on these points even when the disclosure is negative.

Sample size	No statistical methods were used to predetermine sample size for experimentation. For bioinformatics, we used all available RNA-seq data associated with a study or cohort unless specified in the text or supplementary information. For RNA-seq experiments of cell cultures and xenograft tumors, we used three and five biological replicates, respectively. For in vivo therapeutic experiments, we initially implanted 10-15 tumors in castrated NOD/SCID or NSG mice individually per experiment and utilized ones that pass randomization. For in vitro cell-based assays, three technical replicates were included for each experiment, and the experiment was repeated independently at least twice for each assay. For all qPCR analysis, three technical replicates were included for each sample, and data shown for qPCR analysis was from one experiment that was representative of more than equal to two independent experiments. The splicing RT-PCR was performed on at least two to five biological samples.
Data exclusions	No data was excluded from any presented data, unless specified in text or supplementary information. For example, some data was excluded for further bioinformatic analysis due to limitations in either data quality or associated clinical information.
Replication	Using the data quality parameters described above, all experiments shown in the manuscript were reliably reproducible.
Randomization	CRPC xenografts were first implanted in castrated male mice and then subjected to randomization when tumor size reached 150~200 mm3 using a calculation of 1/2 (length × width2). Mice were then randomly assigned to vehicle and treatment groups.
Blinding	Investigators were blind to group allocation. For animal study, randomization was done. For bioinformatic analysis, group classification was based on relevant results.

Reporting for specific materials, systems and methods

We require information from authors about some types of materials, experimental systems and methods used in many studies. Here, indicate whether each material, system or method listed is relevant to your study. If you are not sure if a list item applies to your research, read the appropriate section before selecting a response.

Materials & experimental systems

n/a	Involved in the study	
☐	☒	Antibodies
☐	☒	Eukaryotic cell lines
☒	☐	Palaeontology
☐	☒	Animals and other organisms
☒	☐	Human research participants
☒	☐	Clinical data

Methods

n/a	Involved in the study	
☒	☐	ChIP-seq
☐	☒	Flow cytometry
☒	☐	MRI-based neuroimaging

Antibodies

Antibodies used	AR Mouse mAb Santa Cruz SC-7305 441<br>c-MYC Rabbit mAb Abcam Ab32072 Y69<br>Ki67 Rabbit pAb Leica Biosystems NCL-Ki67p Clone MM1
Validation	All these antibodies are routinely used in our laboratory for years and have been validated for their specificity (which is evidenced in our recent publications: Li Q., et al., Nat Commun 2018 PMID: 30190514; and Li Q., et al., Nat Commun 2019 PMID: 31792211). The IHC staining results were also provided in this manuscript.

Eukaryotic cell lines

Policy information about cell lines

Cell line source(s)	Cell line (LNCaP, PC3, RWPE1, DU145) were commercially obtained from American Type Cell Culture (ATCC). LAPC9 xenograft line was initially provided by Dr. Robert Reiter (UCLA).

Authentication	All cell cultures were regularly authenticated by our institutional CCSG Cell Line Characterization Core using short tandem repeat analysis and checked to be free of mycoplasma contamination using the Agilent (Santa Clara, CA) MycoSensor QPCR Assay Kit (catalogue number 302107).
Mycoplasma contamination	No mycoplasma contamination detected.
Commonly misidentified lines (See ICLAC register)	No commonly misidentified cells were used.

Animals and other organisms

Policy information about studies involving animals; ARRIVE guidelines recommended for reporting animal research

Laboratory animals	Male immunodeficient mice with age around 8-10 weeks, NOD/SCID (non-obese diabetic/severe combined immunodeficiency) and NOD/SCID-IL2Rγ−/− (NSG) were were used in this study and they were originally obtained from the Jackson Laboratory. We maintained a breeding colony in standard conditions in our animal facilities. Male FVB-C-MYC mice aged from 2weeks to 9 month are involved in this study.
Wild animals	No wild animals were used in this study.
Field-collected samples	No Field-collected samples were used in this study.
Ethics oversight	The Laboratory Animal Shared Resource (LASR) at Roswell Park Comprehensive Cancer Center.

Note that full information on the approval of the study protocol must also be provided in the manuscript.

Flow Cytometry

Plots

Confirm that:

- [ ] The axis labels state the marker and fluorochrome used (e.g. CD4-FITC).
- [x] The axis scales are clearly visible. Include numbers along axes only for bottom left plot of group (a 'group' is an analysis of identical markers).
- [x] All plots are contour plots with outliers or pseudocolor plots.
- [ ] A numerical value for number of cells or percentage (with statistics) is provided.

Methodology

Sample preparation	Cell cultures were treated with E7107 at varying concentrations for 2-3 days.
Instrument	BD FACSAria
Software	Diva v8.0.1
Cell population abundance	We only used FACS to gauge the relative size of cells treated with or without the drug, so cell abundance was high.
Gating strategy	Propidium iodide (PI) was added before FACS analysis to separate viable from dead cells. All live cells were used for analysis.

- [ ] Tick this box to confirm that a figure exemplifying the gating strategy is provided in the Supplementary Information.
